# Investigating the implementation of infection prevention and control practices in neonatal care across country income levels: a systematic review

**DOI:** 10.1186/s13756-025-01516-7

**Published:** 2025-02-07

**Authors:** Emanuela Nyantakyi, Julia Baenziger, Laura Caci, Kathrin Blum, Aline Wolfensberger, Angela Dramowski, Bianca Albers, Marta Castro, Marie-Therese Schultes, Lauren Clack

**Affiliations:** 1 Medical Faculty, Institute for Implementation Science in Health Care, University of Zurich, Zurich, 8006 Switzerland; 2 Department of Infectious Diseases and Hospital Epidemiology, University Hospital Zurich, Zurich, 8091 Switzerland; 3 Department of Paediatrics and Child Health, Faculty of Medicine and Health Sciences, Stellenbosch University, Cape Town, 8000 South Africa; 4 Centre of Clinical Nursing Science, University Hospital Zurich, Zurich, 8091 Switzerland

**Keywords:** Infection Prevention, Neonate, Implementation Science Systematic Literature Review, Infection, Healthcare Associated, Neonatal Unit

## Abstract

**Background:**

Despite the proven effectiveness of infection prevention and control (IPC) practices in reducing healthcare-associated infections and related costs, their implementation poses a challenge in neonatal care settings across high-income (HICs) and low- and middle-income countries (LMICs). While existing research has predominantly focused on assessing the clinical effectiveness of these practices in neonatal care, aspects concerning their implementation remain underexplored. This systematic review therefore aimed to analyze implementation determinants and employed strategies for implementing IPC practices in inpatient neonatal care across country income levels.

**Methods:**

Following a targeted search in seven databases, titles and abstracts as well as full texts were screened in a dual review process to identify studies focusing on the implementation of IPC practices in inpatient neonatal care and reporting on implementation determinants and/or implementation strategies. Implementation determinants were synthesized using the updated Consolidated Framework for Implementation Research. Implementation strategies were coded according to the Expert Recommendations for Implementing Change taxonomy. A convergent integrated approach was used to narratively summarize results across qualitative and quantitative studies. *χ*^*2*^ Tests and Fisher’s Exact Tests were performed to analyze differences in implementation determinants and strategies across IPC practices and country income levels. The quality of included studies was assessed using the Mixed Methods Appraisal Tool.

**Results:**

Out of 6,426 records, a total of 156 studies were included in the systematic review. Neonatal units in LMICs and HICs showed general commonalities in reported implementation determinants, which were mainly reported at the organizational level. While educational as well as evaluative and iterative strategies were most frequently employed to support the implementation of IPC practices in both LMICs and HICs, other strategies employed showed variance across country income levels. Notably, the statistical analyses identified a significant association between country income levels and implementation determinants and strategies respectively ($\:\rho\:$<0.05).

**Conclusion:**

The results of this systematic review underscore the importance of the organizational level for the implementation of IPC practices in neonatal care irrespective of country income level. However, further research is needed to understand the underlying relationships of factors and dynamics contributing to the observed practice variances in LMICs and HICs.

**Registration:**

PROSPERO (CRD42022380379).

**Supplementary Information:**

The online version contains supplementary material available at 10.1186/s13756-025-01516-7.

## Background

Infection prevention and control (IPC) practices have been shown to effectively reduce healthcare-associated infections (HAIs) as well as healthcare expenditures [1–3]. In fact, up to 55–70% of HAIs have been estimated to be preventable through effective implementation of existing IPC practices [4–6]. Although the prevalence of HAIs in low- and middle-income countries (LMICs) has been suggested to be at least twice as high as in high-income countries (HICs) [7, 8], the implementation of IPC practices poses a universal challenge in clinical care, irrespective of country income levels and healthcare specialties. The first global report on IPC published by the World Health Organization (WHO) in 2022 explores this implementation lag and underscores the critical role of IPC, especially for the care of vulnerable patient populations such as neonates [9].

Neonatal care settings, and especially neonatal intensive care settings, have been identified as particularly susceptible to high rates of HAIs and infection outbreaks [10, 11]. This is partially attributable to the widespread use of invasive devices [12], and the immature immune system of neonates [12, 13]. Estimating the global impact of HAIs in neonatal care is methodically challenging and often remains limited to individual countries and healthcare sectors [11, 14]. At the individual level, HAIs pose a severe health and mortality risk through potential sequelae, such as sepsis or neurodevelopmental impairment [15–17]. At the collective level, HAIs exacerbate public health expenditures, mainly driven by the prolonged hospitalization of affected patients [18–21].

The neonatal care setting poses challenges to the implementation of IPC practices that distinguish it from IPC in other healthcare settings, and thus requires specialized approaches. Contextual particularities of the neonatal setting include the embeddedness of guardians and families in the care of patients. Although family-centered care describes a specific care paradigm aimed at integrating guardians and families into the care continuum of neonates [22], to a degree, their presence is already inherent to neonatal care environments. This circumstance holds a two-fold significance to the implementation of IPC practices, as consequently, the adherence to certain IPC practices, such as hand hygiene, is not only contingent on the behavior of healthcare professionals, but also guardians and families. Further, the presence of guardians and families introduces a risk of pathogen transmission, colonization and thus the development of HAIs. Other unique features of the neonatal care settings pertain to patient acuity as well as the specificity of certain IPC practices in use, such as skin-to-skin care or the administration of human milk [23, 24]. Notably, several of these setting-specific practices are classically executed and reinforced by guardians and families rather than professionals, which further adds to the contextual specificities of neonatal care settings. Moreover, the length of stay of infants in neonatal intensive care units (NICUs) tends to be longer than that of patients of other intensive care units [25]. Initiatives, such as the Vermont Oxford Network [26] which focuses on quality improvement (QI), or the newly established NeoIPC Clinical Practice Network [27] specifically dedicated to IPC in neonatal care, point to a need to practically explore these idiosyncrasies to improve our understanding of the intricacies of optimal IPC implementation in neonatal care.

A lack of synthesized studies systematically identifying and evaluating aspects related to the implementation of IPC practices in neonatal care exists [24]. Current literature emphasizes clinical outcomes for the appraisal of IPC practices, clearly depicting ‘what’ works, yet not necessarily the ‘how’ tied to their effectiveness, i.e., how IPC practices can be translated and applied into clinical practice effectively [28]. Furthermore, though the use of multicomponent bundles for IPC is recommended by the WHO [4, 29], studies often lack a clear differentiation between clinical IPC practices and implementation strategies employed to support these IPC practices, such as training, or the use of reminders [24, 30]. This adds to the already existing challenge of multicomponent bundles, as it further obscures the extent of effectiveness of single implementation strategies in contrast to the IPC practice. Additionally, under a general assumption of absolute context heterogeneity between LMICs and HICs, studies have predominantly examined these settings separately. Although evidence indicates a substantial epidemiological gap in HAIs across HICs and LMICs [7], the estimated morbidity and mortality burden of HAIs reveal a need for improvement in the field of IPC, irrespective of country income levels. Furthermore, with the globally rising prevalence of antimicrobial resistance [31] and the lingering impact of the Coronavirus disease (COVID-19) pandemic, ensuring the health and safety of patients and healthcare professionals has taken on a significance that transcends national borders. Therefore, studies which incorporate data and derive insights from healthcare settings of both HICs and LMICs could help researchers and clinicians understand whether and to which extent they can translate research findings and implementation approaches across country income levels.

To address the described research gaps, we conducted a systematic review to analyze reported implementation determinants and utilized implementation strategies to support IPC practices in neonatal care across HICs and LMICs using implementation science frameworks. Within this review, implementation determinants describe “factors believed or empirically shown to influence implementation” [32], while implementation strategies define “methods or techniques used to enhance the adoption, implementation, and sustainability of a clinical program or practice” [33].

The objectives of this systematic review were to

i. identify reported implementation determinants for IPC practices in neonatal care,
ii. identify employed implementation strategies to support these IPC practices, and
iii. evaluate whether currently reported implementation determinants and utilized implementation strategies differ across IPC practices and country income levels.

## Methods

The systematic review was conducted in accordance with the Mixed Method Systematic Reviews (MMSR) [34] and Preferred Reporting Items for Systematic Reviews and Meta-Analyses (PRISMA) guidelines 2020 [35].

### Conceptual frameworks

We used the updated Consolidated Framework for Implementation Research (CFIR) [36, 37] and the Expert Recommendations for Implementing Change (ERIC) taxonomy [38, 39] to categorize and analyze reported implementation determinants, and strategies employed to implement IPC in neonatal care settings. The CFIR supports the characterization of implementation determinants across five contextual domains hypothesized to influence implementation: (i) the practice or program being implemented (‘Innovation’), (ii) the sociopolitical and economic context (‘Outer Setting’), (iii) the organizational context (‘Inner Setting’), (iv) the individuals involved in the implementation (‘Individuals’), and (v) the strategies employed to implement practices or programs (‘Implementation Process’). The CFIR has been applied to implementation design and evaluation across a wide range of practices, programs, and disciplines [40]. Changes in the latest iteration of the CFIR include additional constructs in the ‘Inner Setting’ domain and the incorporation of a new subdomain within the ‘Individual’ domain specifying roles of the individuals involved in the process of implementation [37]. The ERIC taxonomy represents a standardized nomenclature to describe implementation strategies, comprising 73 discrete implementation strategies across nine thematic clusters [38, 39].

Unlike theories, which are primarily explanatory, frameworks mainly serve a descriptive purpose and conceptualize ‘empirical phenomena’ using clearly defined semantic units [41]. At the time of their development, the CFIR as well as the ERIC taxonomy addressed a critical need within the field of Implementation Science to improve methodological consistency and comparability across studies. Despite criticisms of both frameworks, for example concerning the delineation of constructs and strategies [42–44], their wide application underscores a conceptual universality that aligned with the aim of this systematic review.

### Search strategy and study selection

We searched the databases Cochrane Central Register of Controlled Trials (CENTRAL), Cumulative Index to Nursing and Allied Health Literature (CINAHL), Excerpta Medica database (Embase), Medical Literature Analysis and Retrieval System Online (MEDLINE), PsycINFO, Scopus and Web of Science for eligible studies in January 2023 (Table 1). Studies had to be published in Danish, English, French, German, Italian, Norwegian, Spanish, or Swedish to be included. More information regarding our search strategy and selection is detailed in a protocol. Search strategies are documented in the supplementary files [45].

**Table 1:** In- and exclusion criteria for study selection

PICOS	Inclusion	Exclusion
Population	Neonates, caregivers, and healthcare professionals in inpatient neonatal care settings (e.g., acute neonatal units, neonatal intensive care units, labor units, postnatal units, pediatric units with admission of neonates)	Non-neonatal setting; Outpatient care; At-home care
Intervention	(Generic and setting-specific) Infection prevention and control (IPC) practices/programs	Non-IPC practices/programs
Comparison	Not applicable	
Outcome(s)	Strategies for implementation of IPC practices AND/OR Determinants to implementation of IPC practices	No implementation strategies AND/OR No implementation determinants
Study design	Randomized controlled trials, quasi-experimental studies, and observational studies	Non-primary study reports; Conference abstracts, reviews, commentaries, monographs

Following de-duplication, identified entries were imported into the systematic review management software Covidence [46]. Titles and abstracts as well as full texts were screened independently by two reviewers. A piloting phase preceded both screening stages. Conflicts were addressed through bilateral discussions, and if necessary, through involvement of a third reviewer.

### Study quality assessment

The quality of included studies was assessed using the Mixed Methods Appraisal Tool (MMAT, Version 2018) [47]. The MMAT specifies quality criteria for *(i)* qualitative studies, *(ii)* quantitative RCTs, *(iii)* quantitative non-randomized studies, *(iv)* quantitative descriptive studies and *(v)* mixed-methods studies [47]. The quality assessment was initially completed by two reviewers and finalized by one reviewer. As recommended by Hong et al. [47], we did not calculate a summarized score and assessed the quality of studies in relation to the defined research objectives.

### Data extraction

One reviewer extracted the following study characteristics using the software MAXQDA 2022 [48]: study authors, country, level of care, study design, aim, primary IPC practice (area), reported implementation determinants and implementation strategies.

### Data synthesis

A convergent integrated approach [34], guided by the methodology of Sattar et al. (2021) [49], was used for data synthesis. Results of qualitative and quantitative studies were synthesized and subsequently integrated.

Following a deductive approach, extracted implementation determinants were mapped to constructs of the updated CFIR [36, 37]. Using an inductive approach, subcodes were defined within these constructs, and iteratively refined by two researchers. For example, subcodes identified within the construct ‘Capability’ include ‘Education Level’ or ‘Memory & Attention’. Further information on specific subcodes is referenced in the codebooks shared in the supplementary material. To gain a clear overview of the high volume of extracted implementation determinants, we performed a quantitative translation. Subcodes identified as barriers or facilitators were assigned a value of ‘-1’ or ‘+1’ respectively, while neutral subcodes (i.e., determinants not explicitly framed as either barriers or facilitators) were assigned a value of ‘0’. Recurring subcodes within a study were summarized and only counted once per study unless framed differently (e.g., as both a barrier and a facilitator). We subsequently calculated the net value of constructs based on the sum of barriers and facilitators. To enable comparability across constructs and to account for variability in net values, we used *z*-standardization and selected extreme value thresholds to identify frequently reported barriers and facilitators. Constructs with *z*-scores ≥ 0.85 percentile were considered ‘frequent facilitators’, whereas those with *z*-scores ≤ 0.15 percentile were categorized ‘frequent barriers’.

Implementation strategies were coded following the ERIC taxonomy [38, 39]. Repetitive strategies within a study were aggregated and normalized relative frequencies across and within ERIC clusters [39] calculated.

To evaluate the association between IPC practices and country income levels with reported implementation determinants and employed strategies, we performed *χ*^*2*^ Test or Fisher’s Exact Test with $\:\rho\:$-value approximation, depending on the characteristics of the dataset.

**Fig. 1:**
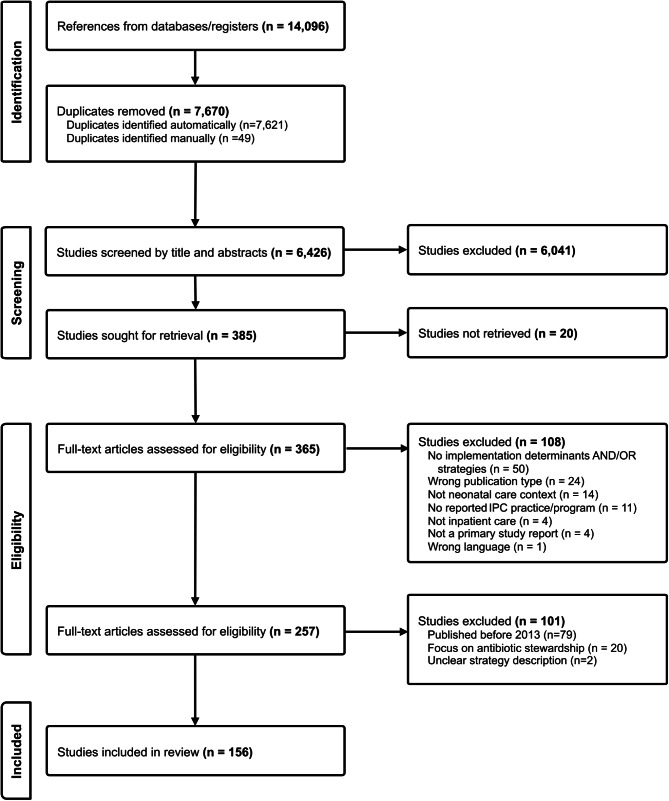
PRISMA flow diagram of study selection

**Fig. 2:**
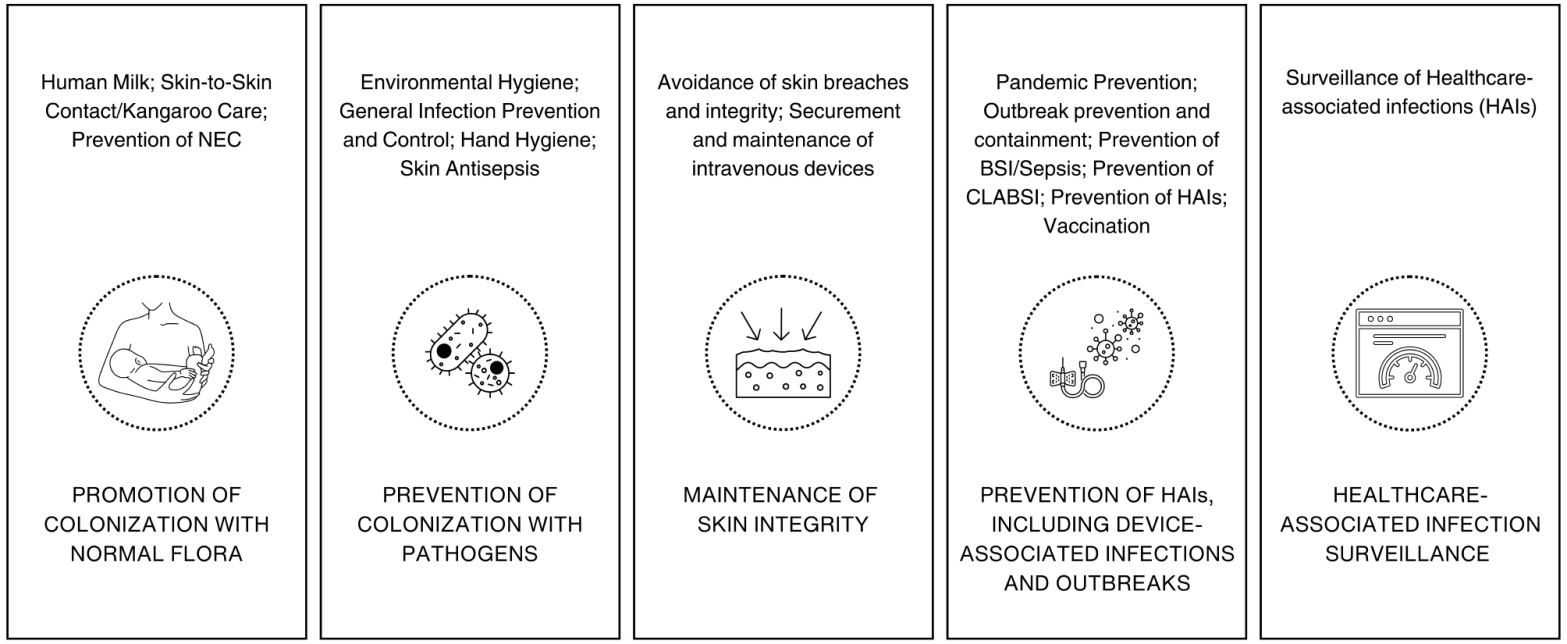
Domains of infection prevention and control practices in neonatal care. classification based on Dramowski et al. (2022) [50]. NEC: Necrotizing enterocolitis; BSI: Bloodstream infections; CLABSI: Central-line associated bloodstream infection

IPC practices were categorized by adapting the framework of Dramowski et al. (2022) [50], which presents a categorization of IPC practices in neonatal care (Fig. 2). The classification of countries into income levels was based on the categorization by the World Bank [51, 52]. MAXQDA 2022 [48], Microsoft^®^ Excel [53] and RStudio 4.2.2 [54] were used to support data synthesis.

### Modifications to codebooks

The codebooks used for implementation determinants and strategies are available in the supplementary files. Modifications made to the ERIC taxonomy included adding the codes ‘Update tools/systems for quality monitoring’ and ‘Update educational materials’. The definition of the strategy ‘Alter incentive/allowance structures’ was also extended to include non-financial incentives. In the case of the CFIR, we developed the code ‘Characteristics of Materials and Equipment’ to account for determinants referring to aspects of user experience, such as the quality or ease of use of available equipment. The CFIR domain ‘Implementation Process’ was excluded, since implementation strategies were categorized using the ERIC taxonomy [38, 39].

### Protocol deviations

We retroactively applied additional exclusion criteria after a first full-text screening iteration to further narrow down the selection of included studies (i.e., *(i)* studies published before the year 2013, *(ii)* studies focused on antibiotic stewardship, *(iii)* studies with brief and unclear strategy descriptions) (Fig. 1). Additionally, given the granularity of coding items as well as the large number of included studies, we decided to mirror an approach taken by Chaudoir et al. (2013) [55] of pursuing single coding after evaluating coding agreement of a subset. We randomly selected 20% of the included studies using RStudio 4.2.2 [54] and assessed Cohen’s κ for inter-rater reliability.

## Results

### Search results

Following duplicate removal, 6,426 records were screened for title and abstracts. A total of 385 records were screened in the full text review and a total of 156 studies were included in the systematic review (Fig. 1).

### Study characteristics

Sixty-nine studies were conducted in inpatient neonatal care settings in LMICs, and 84 studies in HICs. Three studies did not report the study country. IPC practices were most frequently centered around preventing HAIs (*n* = 58) (e.g., central line-associated and catheter-related bloodstream infections prevention) or colonization with pathogens (*n* = 43) (e.g., hand hygiene) (Fig. 2).

Eighteen studies exclusively provided information on implementation determinants, 59 studies exclusively focused on implementation strategies, and 79 studies contained information on both implementation determinants and strategies. An overview of the included studies and extracted data are provided in the supplementary material.

### Quality assessment

The systematic review included different study designs across the MMAT, with non-randomized trials being the predominant type of study conducted (Table 2). Notably, only one study was designed as a randomized controlled trial. While the included studies generally demonstrated a clear research aim and methods in alignment with the research questions of interest, in certain instances, we observed tendencies that compromised the quality of studies which are subsequently described.

**Table 2:** Frequencies of Subcodes

	*n*
Mixed Methods Study	14
Non-Randomized Trial	101
Qualitative Study	28
Quantitative Descriptive Study	12
Randomized Controlled Trial	1

Overall, qualitative studies exhibited thorough methodological descriptions, e.g., of data collection and analysis. However, in a few cases the rationale behind the use of qualitative approaches remained ambiguous (*n* = 3). Studies applying quantitative-descriptive methodologies often lacked clear descriptions of sampling approaches, such as applied in- and exclusion criteria (*n* = 5), and considerations made to address non-response bias (*n* = 10). Similarly, non-randomized trials also frequently lacked information on sampling strategy and in-and exclusion criteria, thus missing crucial information to judge the representativeness of the study population (*n* = 66). Further, potential influences of confounding variables were infrequently addressed (*n* = 85). In the case of mixed-methods studies, the rationale behind the use (*n* = 4) as well as the degree of integration among different methodologies (*n* = 5) was not always consistently defined.

### Reported implementation determinants

**Fig. 3:**
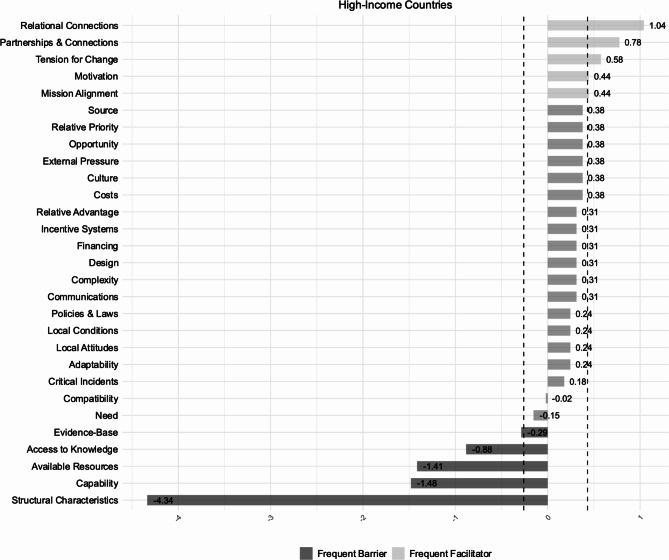
Implementation determinants | High income countries. *z*-scores of CFIR constructs based on reported barriers (*n* = 240) and facilitators (*n* = 104). The dotted lines indicate the threshold values used to categorize implementation determinants as frequent barriers (lower 15%; z ≤ -0.26) and frequent facilitators (upper 15%; z ≥ 0.43)

**Fig. 4:**
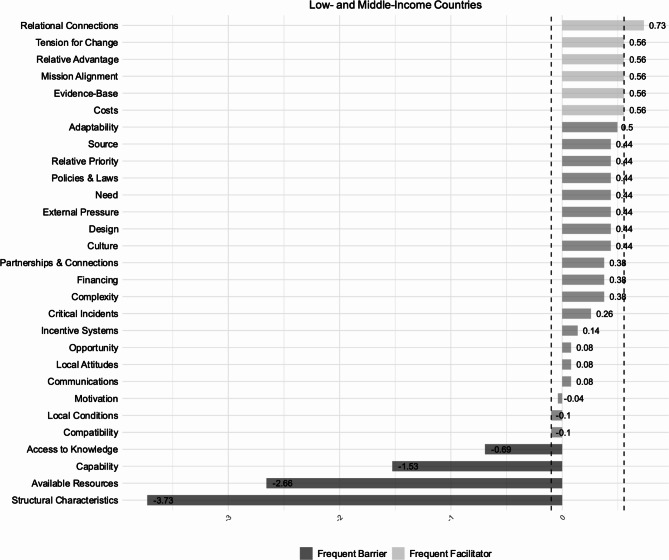
Implementation determinants | Low- and middle-income countries. *z*-scores of CFIR constructs based on reported barriers (*n* = 296) and facilitators (*n* = 83). The dotted lines indicate the threshold values used to categorize implementation determinants as frequent barriers (lower 15%; z≤-0.10) and frequent facilitators (upper 15%; z ≥ 0.56)

We extracted 802 individual subcodes describing implementation determinants (Table 3), of which 52% were reported in studies conducted in LMICs. In the following we present frequent facilitators (constructs with z-scores ≥ 0.85 percentile) and frequent barriers (constructs with z-scores ≤ 0.15 percentile). LMICs and HICs predominantly showed similarities in reported frequent barriers at the organizational level (‘Inner Setting’) (Figs. 3 and 4). Information on the classification methodology of implementation determinants is detailed in the section ‘Data Synthesis’.

**Table 3:** Absolute and normalized relative frequencies of implementation strategies

	Barriers	Facilitators	Neutral	
Innovation	17	15	3	**35**
Inner Setting	382	127	12	**521**
Outer Setting	28	11	5	**45**
Characteristics of the Individuals	114	34	53	**201**
	**541**	**187**	**74**	**802**

The calculated Cohen’s κ to measure inter-rater reliability of the subset coding of implementation determinants was 0.93, indicating a substantial level of agreement between coders.

### Frequent facilitators

‘Relational Connections’ were indicated as a common frequent facilitator across neonatal units in HICs and LMICs, and encompassed different dynamics, including peer support among patient families, (e.g., [56]), teamwork and collaboration among staff (e.g., [57, 58]), or cross-collaboration across teams and departments (e.g., [59, 60]).

A sense of urgency within organizations to implement IPC practices (‘Tension for Change’), was another frequent facilitator reported in studies conducted in HICs and LMICs, often triggered by rising infection rates (e.g., [59, 61]), or outbreaks (e.g., [62, 63]).

Studies in HICs reported additional frequent facilitators to the implementation of IPC practices, such as ‘Partnerships & Connections’, for instance through membership of neonatal units in networks dedicated to specific IPC causes (e.g., [58, 64]). Furthermore, studies conducted in neonatal units in HICs cited an enabling effect of ‘Mission Alignment’, referring to an institutional emphasis on IPC (e.g., [59, 65]), or shared organizational goals and visions (e.g., [66]).

Notably, at the individual level, ‘Motivation’ of innovation deliverers (e.g., [64]), was reported a frequent facilitator to the implementation of IPC practices in studies conducted in HICs. Motivation encompassed several aspects, including a sense of empowerment often fostered by active involvement in the implementation process, as observed in healthcare professionals (e.g., [61]), or parents and guardians (e.g., [64]).

Additional frequent facilitators reported in studies conducted in LMICs involved intervention characteristics, such as the existing evidence surrounding IPC practices (e.g., [67]), their relative advantage in comparison to other practices or programs (e.g., [68]), or low costs (e.g., [64, 69]).

### Frequent barriers

‘Structural Characteristics’ were a frequent organizational barrier across studies conducted in HICs and LMICs and involved various aspects in relation to different elements of the organizational infrastructure. For instance, a lack of clear responsibilities between different professional groups (e.g. [65, 70]), fluctuating staffing levels and staff shortages (e.g., [71–73]), or a lack of specialized IPC teams and staff (e.g. [74]), were cited with regards to ‘Work Infrastructure’. The layout of units (e.g [75, 76]), and the location and accessibility of materials and equipment (e.g., [65, 77, 78]), were reported limitations related to ‘Physical Infrastructure’.

‘Available Resources’ represented another frequent barrier reported across country income levels, albeit highly differing in frequency (Figs. 3 and 4). Physical space limitations (e.g., [60, 68]), often connected to overcrowding (e.g., [67, 79]), hindered the appropriate execution of IPC practices, such as cohorting. Financial constraints limited budget allocations for IPC (e.g., [75, 80]), and the availability of materials and equipment (e.g., [70, 81, 82]), which in some instances resulted from procurement issues (e.g., [81]), also posed a challenge to the implementation of IPC practices. Additionally, the characteristics of materials and equipment reportedly impeded implementation. Studies highlighted user discomfort, such as skin irritation from handrub use (e.g., [76, 83]), and equipment deficiencies, for example regarding cleanliness (e.g., [74]).

‘Access to Knowledge’ presented another shared frequent barrier reported in neonatal units across LMICs and HICs. This included a lack of training opportunities on IPC practices or programs for staff, patient families, and guardians (e.g., [80, 84]), as well as the absence of adequate educational material (e.g., [73, 84]). Additionally, inconsistent, and contradictory information regarding IPC practices or institutional processes was reported as a challenge to implementation (e.g., [60, 85]).

Furthermore, at the individual level (‘Characteristics of Individuals’), the ‘Capability’ of innovation deliverers predominantly healthcare professionals, and in some instance guardians or families, was cited a frequent barrier across country income levels. This included the varying educational background of some parents, necessitating the use of diverse information media to educate them on IPC practices (e.g., [86]). It also concerned the professional skill level among healthcare professional groups required to effectively execute IPC practices, including lab technicians [86]), nurse practitioners (e.g., [87]), nurses (e.g., [87, 88]), and auxiliary staff, such as cleaning staff (e.g., [89]). Additionally, in studies conducted in HICs, ‘Need’ represented a frequent barrier at the individual level, and commonly involved patient acuity (e.g., [90, 91]).

### Reported implementation strategies

We extracted 1’066 distinct strategies and coded them according to 59 ERIC strategies (Table 4). The median number of applied ERIC strategies per study was 5, ranging from 1 to 27 individual ERIC strategies per study. Values in parentheses indicate absolute and normalized relative frequencies.

**Table 4:** Absolute and normalized relative frequencies of implementation strategies

	All	HIC	LMIC	
Adapt and tailor to context	41 [0.04]	19 [0.03]	22 [0.05]	
Change infrastructure	111 [0.10]	71 [0.11]	37 [0.09]	
Develop relationships	160 [0.15]	108 [0.17]	50 [0.12]	
Engage involved individuals or groups	32 [0.03]	21 [0.03]	11 [0.03]	
Provide interactive assistance	41 [0.04]	20 [0.03]	19 [0.05]	
Support clinicians or other involved groups	65 [0.06]	35 [0.06]	27 [0.07]	
Train and educate	307 [0.29]	181 [0.28]	121 [0.30]	
Use evaluative and interactive strategies	285 [0.27]	165 [0.26]	111 [0.27]	
Utilize incentivizing/financial strategies	24 [0.02]	16 [0.03]	8 [0.02]	
	**1’066**	**636**	**406**	

Clusters of ERIC Implementation strategies based on Waltz et al. [39]. Values depict absolute frequencies and normalized relative frequencies in parentheses. Discrepancies between “All” and frequency of studies categorized by country-income level due to two studies not reporting the country

While in the included studies conducted in LMICs, implementation strategies were commonly used to enhance hand hygiene practices (*n* = 98 [0.24]), in HICs, they were more commonly utilized to prevent central line-associated and catheter-related bloodstream infections (*n* = 216 [0.34]).

Studies often did not systematically report the actors and target groups involved in implementation strategies. Nevertheless, implementation strategies were executed by and targeted toward a diverse range of groups. For instance, several implementation strategies related to hand hygiene were reportedly directed at nurses (e.g., [92]), while others specifically targeted parents and family members (e.g., [61, 93, 94]). In contrast, strategies employed in relation to environmental hygiene programs also focused on cleaning staff (e.g., [77]). A comprehensive list of actors and target groups represented is provided in the supplementary materials.

More than half of the identified strategies involved education and training as well as iterative and evaluative strategies. Studies detailed the provision of educational meetings or training sessions (*n* = 94 [0.31]), predominantly aimed at healthcare professionals, such as nurses (e.g., [95, 96]), to support the implementation of various IPC practices. Additionally, dissemination of educational materials, including visual posters, brochures, or guidelines (e.g., [96–98]), was a commonly employed strategy (*n* = 65 [0.21]). Pertaining to iterative and evaluative strategies, the organization of quality monitoring systems (*n* = 57 [0.20]), along with the utilization of audit and feedback (*n* = 50 [0.18]), were commonly reported strategies to support the implementation of IPC practices across both LMICs and HICs. Conversely, the adoption of formalized action plans appeared to be used less frequently to implement IPC practices (*n* = 10 [0.04]).

Strategies within the cluster ‘Develop relationships’ were the third most frequently used strategy type (n = 160 [0.15]). Studies often detailed the formation of multidisciplinary work groups (n = 53 [0.33]), typically QI teams, to support or lead local implementation efforts (e.g., [99–101]). The establishment or participation of neonatal units in networks or collaboratives was more commonly reported in studies conducted in HICs (n = 17 [0.16]) than in LMICs (n = 2 [0.04]).

Reported strategies within the cluster ‘Change Infrastructure’ often focused on altering the physical environment or equipment (*n* = 58 [0.52]). Examples included relocating or fixating hand rub dispensers (e.g., [102–104]), or creating designated areas and spaces, for instance to support skin-to-skin contact (e.g., [105]). Further, studies conducted in units in HICs commonly introduced changes to record or documentation systems (*n* = 19 [0.27]).

Strategies of the cluster ‘Engage involved individuals or groups’ (*n* = 32 [0.03]), ‘Provide interactive assistance’ (*n* = 41 [0.04]), ‘Support Individuals’ (*n* = 65 [0.06]) and ‘Utilize incentivizing/financial strategies’ (n = 24 [0.02]), were reported less commonly to support the implementation of IPC practices. Similarly, tailored strategies were not reported frequently (n = 41 [0.04]) and rarely defined *a priori* (e.g., [28]), as they often emerged in response to specific local conditions, e.g., adapting educational materials to cater to language preferences (e.g., [61]), relocating materials during the COVID-19 pandemic (e.g., [77]), or adjusting the timing of educational activities to accommodate shift schedules (e.g., [101, 106]).

The calculated Cohen’s κ in the subset coding of implementation strategies was 0.94, suggesting a high level of inter-rater reliability among coders.

### Association of Implementation Determinants and strategies with IPC practices and Country Income levels

The results of the Fisher’s Exact Test indicated a significant difference of reported implementation determinants at CFIR domain-level across IPC practices ($\:\rho\:$<0.05). Furthermore, at country-income level, the association with reported implementation determinants across CFIR domains (χ^2^ (3, *N*=802) = 11.00, $\:\rho\:$<0.05) and CFIR constructs ($\:\rho\:$<0.05) was significant. A post hoc analysis using standardized residuals revealed that the organizational level (‘Inner Setting’) had the most impact on the significant difference at domain-level, while ‘Innovation Evidence Base’ had the most impact on the significant association at construct-level. Notably, the sample size of the ‘Innovation’ domain was relatively low (*n* = 35). Therefore, the results of the post hoc analysis at construct-level should be interpreted with caution. However, the results highlight the importance of the organizational context level for implementing IPC practices in neonatal care despite variability regarding the relevance of individual implementation determinants across HICs and LMICs.

The results of the Fisher’s Exact Test revealed no significant difference of employed implementation strategies across IPC practices ($\:\rho\:$>0.05). However, the types of reported strategies across country income level were shown to significantly differ (χ^2^ (8, *N*=1042) = 247.19, $\:{\uprho\:}$<0.05), suggesting a level of context specificity of employed implementation strategies across HICs and LMICs. A post hoc test revealed that the strategy clusters contributing most to the observed significant association were ‘Utilize incentivizing/financial strategies’, ‘Use evaluative and iterative strategies’ and ‘Change infrastructure’.

## Discussion

This systematic review represents the first investigation of reported implementation determinants and implementation strategies related to IPC practices in neonatal care across HICs and LMICs. It comprehensively examined 156 studies using a mixed-methods approach.

Utilized IPC practices predominately targeted prevention of HAIs and pathogen colonization. Further, they usually involved generic IPC practices, i.e., IPC practices that are not specific to the neonatal care setting, such as environmental cleaning or hand hygiene. Setting-specific IPC practices, such as skin-to-skin contact or probiotic administration to promote colonization with normal flora, were reported less frequently. Studies conducted in LMICs and HICs showed strong commonalities in frequently reported barriers and facilitators, particularly structural characteristics at the organizational level (e.g., staffing levels or unit admission policies). However, the frequency of certain common factors, such as resource availability, differed between studies conducted in LMICs and HICs. Although the statistical analyses revealed no significant differences in the type of employed strategies across IPC practices, the results suggested significantly different applications of implementation strategies, such as the use of evaluative and iterative strategies, across country income levels.

One of the few explorations on this topic, a narrative review on reported implementation determinants to IPC practices conducted by authors of this systematic review, also identified the relevance of the organizational setting for implementation in the neonatal care setting [24]. However, in current literature, the evaluation of evidence-based practices in neonatal care, including IPC, has either mainly focused on their clinical effectiveness, or has actively segregated LMICs and HICs [13, 107–113]. A rationale for the separation is related to epidemiology, since LMICs tend to have higher morbidity and mortality rates of infections, including HAIs [7]. Another rationale seemingly stems from assumptions surrounding resource availability, as LMICs are usually conceptualized as resource-limited settings. However, resource-limited settings can also be found in HICs, e.g., in socially deprived areas, which have been suggested to be prone to HAIs [114] and adverse events in maternal and neonatal care [115, 116]. The tendency to analyze HICs and LMICs separately has potentially left the scope of differing and converging aspects of contextual conditions and implementation challenges unclear. In lieu of this, the observed difference in applied strategies across neonatal units in LMICs and HICs poses the question of whether these practice variances are influenced by context or driven by inherent biases. These biases might be perpetuated through over- or underestimating the influence of certain implementation determinants, such as resource availability, or epidemiological factors, on implementation. A thorough understanding of this could foster mutual learning and the development of cross-contextual implementation approaches. The latter prove particularly relevant from a global public health perspective, given the recent emphasis of the global role of IPC, particularly within the care of vulnerable patient populations by the WHO [9]. Yet, it is important to highlight the need for equitable engagement between actors from LMICs and HICs for potential cross-contextual translations of IPC implementation. Hence, the transfer of (best) practices should occur in bidirectional partnerships between researchers from LMICs and HICs; this also to avoid the perils of ‘isomorphic mimicry’ [117], wherein healthcare organizations superficially adopt practices and infrastructures of external entities from other contexts that ultimately render themselves inefficient and ineffective.

The reviewed studies predominantly applied implementation approaches that relied on linear rather than systems methodologies, meaning identified implementation determinants were usually targeted specifically and in isolation. This observation, also highlighted in the aforementioned narrative review [24], is reflective of an overarching methodological issue in the field of Implementation Science [118–121]. Current research and practice approaches tend to exclude the relational dynamics tied to implementation. While this reductionist perspective may simplify the latent and complex dynamics exhibited in real-world conditions, it risks inadequately capturing and addressing the needs of the context and perspectives of involved subjects, compromising the sustainment of practices [119]. In the case of IPC, adopting approaches that recognize the multidimensionality of implementation can elucidate how contextual variables and strategies interact, ultimately improving implementation outcomes. Such approaches could also help address the varying needs and involvement of relevant groups, as the implementation of IPC practices and programs requires collaboration among a wide range of healthcare professionals, such as neonatologists, IPC practitioners, nurses or microbiologists.

A major strength of this systematic review lies in its comprehensive analysis of implementation determinants and strategies across a diverse range of IPC practices and country income levels, thus addressing significant research gaps in neonatal care. Our inclusive search strategy, encompassing eight languages, facilitated a thorough examination of relevant literature. However, it should be noted that due to the composition of our review team, only European languages were represented.

As a result of the large volume of extracted data, we decided to use quantitative methods to assess implementation determinants and strategies to different extents. In the case of implementation determinants, we calculated the sum and *z*-scores of individual constructs using an ordinal scale (‘+1’ [facilitator], ‘0’ [neutral], ‘-1’ [barrier]). In the case of implementation strategies, we calculated the normalized relative frequencies. Both approaches accounted for variations within and across the datasets of LMICs and HICs and supported the comparisons within and across country income levels. Triangulating qualitative data with quantification therefore enhanced comparability and assessment, as it enabled us to identify and derive key patterns in our data [122]. Additionally, the large sample size of cases (i.e., of included studies) and variables (i.e., implementation determinants and strategies) made the quantitative translation approach less prone to inaccurate representations of our data [122].

A few limitations should be acknowledged. The use of frameworks such as the CFIR and ERIC supported the methodological categorization and analysis of implementation determinants and strategies within this systematic review. Nevertheless, adjustments were required to adequately capture relevant implementation determinants and utilized strategies. Additionally, while the CFIR provides detailed constructs at the meso level, it exhibits a lack of granularity at the macro level. This limitation became particularly evident when coding a study focused on macro-level implementation determinants across countries [72]; most implementation determinants were attributed to ‘Local Conditions’. While this attribution was conceptually fitting, it did not fully capture the intricacies of the macro level, which encompassed diverse factors. Our analyses therefore highlight a need for determinant frameworks within Implementation Science which adequately describe the sociopolitical context [123]. Notably, there have been recent attempts to explore the integration of policy implementation research within the field of Implementation Science [124, 125].

While the MMAT enabled the quality appraisal of a wide range of studies, its quality indicators are mainly linked to conventional study characteristics, such as clearly stated research aims [47]. For investigations like ours, where the research objectives of interest are independent of conventional study characteristics, a quality assessment tool that examines additional aspects (e.g., the data collection characteristics of implementation determinants or the operationalization of implementation strategies), might have provided a more comprehensive evaluation. It may have more appropriately captured the interplay between conventional study quality characteristics and the quality of implementation reporting and resulting implications for our research. To our knowledge, no such integrated quality assessment instrument spanning across different study designs currently exists.

It is crucial to recognize that our findings primarily point to the frequency, and therefore prominence of implementation determinants and applied implementation strategies within and across current studies in HICs and LMICs. Particularly in the case of implementation determinants, frequency implies practical relevance, but it does not necessarily reflect the determinants’ influence on implementation. Furthermore, certain trends observed in implementation determinants and implementation strategies showed concordance. For instance, we identified the membership of units in networks as a key facilitator and a frequently reported implementation strategy in studies conducted in HICs. Yet, we did not systematically explore the relationship between context (implementation determinants) and practice (implementation strategies), leaving the alignment between these aspects within and across country income levels unaddressed.

The large volume of studies posed methodological challenges. We therefore decided to pursue single coding after double-coding a subset. Even though the calculated Cohen’s κ for the subset coding of implementation strategies and determinants indicated substantial agreement among coders, this approach may have still impacted the reliability of our analyses. Additionally, we chose to limit the time period of interest, potentially overlooking temporal trends in IPC practices. For example, the prevalence of certain practices, such as the use of central lines evolved over time, affecting their relevance to our review. Nevertheless, this approach may have ensured the relevance of our findings to current practices in neonatal care.

## Conclusion

Our findings highlight the crucial role of contextual factors at the organizational level in implementing IPC practices in neonatal care settings. While existing research suggests substantial differences in these contextual factors between neonatal care settings in HICs and LMICs, the results of this systematic review indicate that these variations may be less pronounced than widely assumed. Recognizing the relative nature of these differences opens new avenues for research and practice in IPC implementation across diverse healthcare settings. It can also inform the design of effective implementation approaches by enhancing our understanding of implementation. Nevertheless, the limitations and suitability of cross-contextual translations should be critically examined.

Additional research is needed to unravel the underlying causes and dynamics of observed practice variances across country income levels. Utilizing systems approaches that account for the complex interplay related to implementation could prove particularly helpful in deciphering contextual idiosyncrasies tied to the healthcare setting versus cultural, sociopolitical, and economic aspects influencing practice and the prevalence of HAIs [114, 126].

## Electronic supplementary material

Below is the link to the electronic supplementary material.

Additional File 1: Overview of Studies and extracted Data.

Additional File 2: Actors and Target Groups of extracted Implementation Strategies.

Additional File 3: Coding Manual for Implementation Determinants based on updated Consolidated Framework for Implementation Research (CFIR).

Additional File 4: Coding Manual for Implementation Strategies based on Expert Recommendations for Implementing Change.

Additional File 5: Database Search Strategies.

Additional File 6: PRISMA 2020 Checklist.

## Data Availability

Data generated or analyzed during this study are included in this published article and its supplementary information files. Further data are available from the corresponding author on reasonable request.

## Abbreviations

CENTRAL: Cochrane Central Register of Controlled Trials
CFIR: Consolidated Framework for Implementation Research
CINAHL: Cumulative Index to Nursing and Allied Health Literature
Embase: Excerpta Medica database
ERIC: Expert Recommendations for Implementing Change
HAI(s): Healthcare-associated infection(s)
HIC(s): High-income country/(-ies)
IPC: Infection prevention and control
LMIC(s): Low-and middle-income country/(-ies)
MEDLINE: Medical Literature Analysis and Retrieval System Online
MMAT: Mixed Methods Appraisal Tool
MMSR: Mixed Method Systematic Reviews
NICU(s): Neonatal intensive care unit(s)
PICOS: Population, Intervention, Outcome, Comparators, Study design
PRISMA: Preferred Reporting Items for Systematic Reviews and Meta-Analyses
QI: Quality improvement
WHO: World Health Organization
