# Supplementary material for: Investigating the implementation of infection prevention and control practices in neonatal care across country income levels: a systematic review
Source: Antimicrob Resist Infect Control. 2025 Feb 7;14:8. doi: 10.1186/s13756-025-01516-7 (PMC11806577; doi:10.1186/s13756-025-01516-7)
Supplement: Supplementary file 1 — Additional File 1: Overview of Studies and extracted Data. [file 13756_2025_1516_MOESM1_ESM.pdf]

Investigating the Implementation of Infection Prevention and Control Practices in Neonatal Care Across Country Income Levels: A Systematic Review

Emanuela Nyantakyi, Julia Baenziger, Laura Caci, Kathrin Blum, Aline Wolfensberger, Angela Dramowski, Bianca Albers, Marta Castro, Marie-Therese Schultes, Lauren Clack

SUPPLEMENTARY FILE

Overview of Included Studies & Extracted Data

Abbreviations

BSI	Bloodstream Infection
CLABSI/CRBSI	Central-line associated Bloodstream Infection/Catheter-related Bloodstream Infection
IPC	Infection Prevention and Control
HAI	Healthcare-associated Infection
HIC	High-Income Country
ImD	Implementation Determinants
ImS	Implementation Strategies
LMIC	Low-or Middle-Income Country
NEC	Necrotizing Enterocolitis
VAP	Ventilator-associated Pneumonia

Table of Contents

Study Characteristics 4
Implementation Determinants 14
Implementation Strategies 25
References 41

Study Characteristics

Authors	Year	Study Design	IPC Practice/Area	Country	Income Level	Content
Alimohammadzadeh *et al.* [1]	2017	Mixed Methods Study	IPC, unspecified	Iran	LMIC	ImD/ImS
Almeida *et al.* [2]	2017	Quantitative Descriptive Study	Sepsis	Portugal	HIC	ImS
Alrumi *et al.* [3]	2020	Non-Randomized Trial	IPC, unspecified	Palestinian Territories	LMIC	ImD/ImS
Alshaikh *et al.* [4]	2015	Non-Randomized Trial	Human Milk	Canada	HIC	ImS
Alslaim *et al.* [5]	2022	Mixed Methods Study	IPC, unspecified	United States	HIC	ImD/ImS
Amaan *et al.* [6]	2022	Non-Randomized Trial	Hand Hygiene	Bangladesh	LMIC	ImD/ImS
Anderson [7]	2016	Quantitative Descriptive Study	Probiotics	United States	HIC	ImD
Aragona *et al.* [8]	2021	Mixed Methods Study	Pandemic Prevention	United States	HIC	ImD
Arena *et al.* [9]	2013	Non-Randomized Trial	Outbreak Containment	Italy	HIC	ImS
Azab *et al.* [10]	2015	Non-Randomized Trial	VAP Prevention	Egypt	LMIC	ImS
Azmeraw Getie *et al.* [11]	2022	Qualitative Study	Kangaroo Care	Ethiopia	LMIC	ImD
Badparva *et al.* [12]	2023	Qualitative Study	CLABSI/CRBSI Prevention	Iran	LMIC	ImD
Balachander *et al.* [13]	2020	Non-Randomized Trial	Outbreak Containment	India	LMIC	ImS
Balla *et al.* [14]	2018	Non-Randomized Trial	CLABSI/CRBSI Prevention	n/a	n/a	ImD/ImS
Barrett *et al.* [15]	2023	Non-Randomized Trial	Pandemic Prevention	United States	HIC	ImD/ImS

Authors	Year	Study Design	IPC Practice/Area	Country	Income Level	Content
Batthula *et al.* [16]	2021	Non-Randomized Trial	Sepsis	India	LMIC	ImD/ImS
Bechmann *et al.* [17]	2023	Non-Randomized Trial	Outbreak Containment	Germany	HIC	ImS
Beekman & Steward [18]	2020	Non-Randomized Trial	Skin Antisepsis	United States	HIC	ImD/ImS
Bezerra *et al.* [19]	2021	Quantitative Descriptive Study	Hand Hygiene	Brazil	LMIC	ImD
Bharadwaj *et al.* [20]	2019	Non-Randomized Trial	Outbreak Containment	Singapore	HIC	ImD/ImS
Bierlaire *et al.* [21]	2021	Non-Randomized Trial	CLABSI/CRBSI Prevention	Belgium	HIC	ImD/ImS
Biswas *et al.* [22]	2019	Non-Randomized Trial	Hand Hygiene	India	LMIC	ImD/ImS
Bowen *et al.* [23]	2017	Non-Randomized Trial	BSI Prevention	Australia	HIC	ImD/ImS
Cantey *et al.* [24]	2013	Non-Randomized Trial	Outbreak Containment	United States	HIC	ImD/ImS
Caspari *et al.* [25]	2017	Non-Randomized Trial	CLABSI/CRBSI Prevention	United States	HIC	ImD/ImS
Ceballos *et al.* [26]	2013	Non-Randomized Trial	HAI Prevention	United States	HIC	ImD/ImS
Chandonnet *et al.* [27]	2017	Non-Randomized Trial	Hand Hygiene	United States	HIC	ImD/ImS
Chandonnet *et al.* [28]	2013	Qualitative Study	CLABSI/CRBSI Prevention	United States	HIC	ImD/ImS
Cowden *et al.* [29]	2020	Qualitative Study	IPC, unspecified	Zambia	LMIC	ImD/ImS
Cross *et al.* [30]	2016	Qualitative Study	Environmental Cleaning	Multiple	LMIC	ImD
Cross *et al.* [31]	2019	Mixed Methods Study	Environmental Cleaning	Multiple	LMIC	ImD

Authors	Year	Study Design	IPC Practice/Area	Country	Income Level	Content
Dawczynski *et al.* [32]	2017	Quantitative Descriptive Study	IPC, unspecified	Germany	HIC	ImD
Delaney Manthe *et al.* [33]	2019	Qualitative Study	Human Milk	United States	HIC	ImD/ImS
Deshommes *et al.* [34]	2021	Non-Randomized Trial	Hand Hygiene	Haiti	LMIC	ImD/ImS
Dramowski *et al.* [35]	2021	Non-Randomized Trial	Environmental Cleaning	South Africa	LMIC	ImD/ImS
Dumpa *et al.* [36]	2019	Non-Randomized Trial	CLABSI/CRBSI Prevention	United States	HIC	ImD/ImS
Dye *et al.* [37]	2021	Qualitative Study	CLABSI/CRBSI Prevention	United States	HIC	ImD/ImS
Erdei *et al.* [38]	2015	Qualitative Study	CLABSI/CRBSI Prevention	United States	HIC	ImD/ImS
EsquéRuiz *et al.* [39]	2015	Qualitative Study	IPC, unspecified	Spain	HIC	ImD/ImS
Fabbri *et al.* [40]	2013	Non-Randomized Trial	Outbreak Containment	Italy	HIC	ImD/ImS
Fernández-Prada *et al.* [41]	2019	Non-Randomized Trial	Outbreak Containment	Spain	HIC	ImS
Ferry *et al.* [42]	2020	Non-Randomized Trial	Outbreak Containment	France	HIC	ImS
Fisher *et al.* [43]	2013	Non-Randomized Trial	CLABSI/CRBSI Prevention	United States	HIC	ImD/ImS
Fischer Fumeaux *et al.* [44]	2017	Non-Randomized Trial	Human Milk	France	HIC	ImD
Gafirimbi *et al.* [45]	2016	Qualitative Study	HAI Prevention	Rwanda	LMIC	ImD/ImS
Gajic *et al.* [46]	2021	Non-Randomized Trial	Outbreak Containment	Serbia	LMIC	ImS
García González *et al.* [47]	2017	Non-Randomized Trial	HAI Prevention	Spain	HIC	ImS

Authors	Year	Study Design	IPC Practice/Area	Country	Income Level	Content
Gephart *et al.* [48]	2021	Mixed Methods Study	NEC Prevention	United States	HIC	ImD/ImS
Gephart & Quinn [49]	2019	Mixed Methods Study	NEC Prevention	United States	HIC	ImD/ImS
Geraci *et al.* [50]	2014	Quantitative Descriptive Study	HAI Prevention	Italy	HIC	ImS
Giuffrè *et al.* [51]	2013	Non-Randomized Trial	Outbreak Containment	Italy	HIC	ImS
Gon *et al.* [52]	2017	Mixed Methods Study	IPC, unspecified	Tanzania	LMIC	ImD/ImS
Gon *et al.* [53]	2021	Mixed Methods Study	Environmental Cleaning	Tanzania	LMIC	ImD/ImS
Goodchild *et al.* [54]	2018	Non-Randomized Trial	Human Milk	Australia	HIC	ImD/ImS
Gopalakrishnan *et al.* [55]	2021	Non-Randomized Trial	Hand Hygiene	India	LMIC	ImS
Grover *et al.* [56]	2015	Qualitative Study	CLABSI/CRBSI Prevention	United States	HIC	ImS
Hamza *et al.* [57]	2022	Non-Randomized Trial	CLABSI/CRBSI Prevention	Egypt	LMIC	ImS
Hanley *et al.* [58]	2022	Qualitative Study	Pandemic Prevention	United Kingdom	HIC	ImD/ImS
Hawes & Lee [59]	2018	Qualitative Study	CLABSI/CRBSI Prevention	Canada	HIC	ImD/ImS
Hayashi *et al.* [60]	2021	Non-Randomized Trial	Vaccination	United States	HIC	ImD/ImS
Hensel *et al.* [61]	2017	Non-Randomized Trial	Outbreak Containment	Germany	HIC	ImS
Herbeć *et al.* [62]	2020	Qualitative Study	IPC, unspecified	Zimbabwe	LMIC	ImD/ImS
Hightower *et al.* [63]	2022	Qualitative Study	CLABSI/CRBSI Prevention	United States	HIC	ImD/ImS

Authors	Year	Study Design	IPC Practice/Area	Country	Income Level	Content
Howard-Jones *et al.* [64]	2022	Non-Randomized Trial	Outbreak Containment	Australia	HIC	ImD/ImS
Hussain *et al.* [65]	2021	Non-Randomized Trial	CLABSI/CRBSI Prevention	Pakistan	LMIC	ImS
Jacobs Pepin *et al.* [66]	2019	Non-Randomized Trial	VAP Prevention	United States	HIC	ImD/ImS
Jahan *et al.* [67]	2022	Non-Randomized Trial	VAP Prevention	Bangladesh	LMIC	ImS
Jain *et al.* [68]	2023	Non-Randomized Trial	Kangaroo Care	India	LMIC	ImD/ImS
Johnson *et al.* [69]	2022	Non-Randomized Trial	HAI Prevention	India	LMIC	ImS
Kallam *et al.* [70]	2018	Non-Randomized Trial	Hand Hygiene	Ghana	LMIC	ImD/ImS
Karabay *et al.* [71]	2019	Non-Randomized Trial	Outbreak Containment	Turkey	LMIC	ImS
Khurana *et al.* 2018 [72]	2018	Non-Randomized Trial	HAI Prevention	India	LMIC	ImS
Kumar *et al.* [73]	2022	Non-Randomized Trial	Hand Hygiene	India	LMIC	ImD/ImS
Lauderbaugh *et al.* [74]	2019	Non-Randomized Trial	VAP Prevention	United States	HIC	ImD/ImS
Lee *et al.* [75]	2015	Non-Randomized Trial	IPC, unspecified	Canada	HIC	ImS
Linam *et al.* [76]	2019	Non-Randomized Trial	HAI Prevention	United States	HIC	ImS
López *et al.* [77]	2013	Non-Randomized Trial	Sepsis Prevention	Nicaragua	LMIC	ImD/ImS
Madrid-Aguilar *et al.* [78]	2019	Non-Randomized Trial	BSI Prevention	Spain	HIC	ImD/ImS
Mahieu *et al.* [79]	2022	Non-Randomized Trial	CLABSI/CRBSI Prevention	Belgium	HIC	ImD/ImS

Authors	Year	Study Design	IPC Practice/Area	Country	Income Level	Content
Manerkar *et al.* [80]	2022	Non-Randomized Trial	Human Milk	India	LMIC	ImD/ImS
Manzo *et al.* 2022 [81]	2022	Mixed Methods Study	CLABSI/CRBSI Prevention	Brazil	LMIC	ImD
Maria *et al.* [82]	2022	Non-Randomized Trial	Human Milk	India	LMIC	ImD/ImS
Maria *et al.* [83]	2022	Qualitative Study	Hand Hygiene	India	LMIC	ImD/ImS
Marofi *et al.* [84]	2017	Non-Randomized Trial	HAI Prevention	Iran	LMIC	ImS
Marom *et al.* [85]	2020	Non-Randomized Trial	Outbreak Containment	Israel	HIC	ImS
McCord *et al.* [86]	2019	Quantitative Descriptive Study	Skin Antisepsis	Canada	HIC	ImD/ImS
Morgan *et al.* [87]	2018	Mixed Methods Study	Kangaroo Care	Uganda	LMIC	ImD/ImS
Mukerji *et al.* [88]	2013	Non-Randomized Trial	Hand Hygiene	Canada	HIC	ImD/ImS
Mwananyanda *et al.* [89]	2019	Non-Randomized Trial	BSI Prevention	Zambia	LMIC	ImS
Neill *et al.* [90]	2016	Quantitative Descriptive Study	BSI Prevention	United States	HIC	ImS
Németh *et al.* [91]	2022	Quantitative Descriptive Study	Hand Hygiene	Hungary	HIC	ImD/ImS
Ngugi *et al.* [92]	2019	Mixed Methods Study	Hand Hygiene	Kenya	LMIC	ImD/ImS
Oko *et al.* [93]	2022	Non-Randomized Trial	Hand Hygiene	Nigeria	LMIC	ImD
Pahwa *et al.* [94]	2018	Qualitative Study	IPC, unspecified	India	LMIC	ImD/ImS
Pallotto *et al.* [95]	2017	Non-Randomized Trial	CLABSI/CRBSI Prevention	United States	HIC	ImS

Authors	Year	Study Design	IPC Practice/Area	Country	Income Level	Content
Parga *et al.* [96]	2017	Non-Randomized Trial	Hand Hygiene	United States	HIC	ImD/ImS
Pasricha *et al.* [97]	2021	Non-Randomized Trial	Hand Hygiene	Canada	HIC	ImD/ImS
Patel *et al.* [98]	2014	Non-Randomized Trial	NEC Prevention	United States	HIC	ImS
Pettit *et al.* [99]	2017	Non-Randomized Trial	Skin Antisepsis	United States	HIC	ImS
Phan *et al.* [100]	2018	Non-Randomized Trial	Hand Hygiene	Viet Nam	LMIC	ImS
Phan *et al.* [101]	2020	Non-Randomized Trial	CLABSI/CRBSI Prevention	Viet Nam	LMIC	ImS
Pharande *et al.* [102]	2018	Non-Randomized Trial	Sepsis	Australia	HIC	ImD/ImS
Piazza *et al.* [103]	2016	Non-Randomized Trial	CLABSI/CRBSI Prevention	United States	HIC	ImS
Pletsch *et al.* [104]	2013	Qualitative Study	Human Milk	Canada	HIC	ImD/ImS
Prashantha *et al.* [105]	2019	Non-Randomized Trial	Sepsis Prevention	India	LMIC	ImS
Profit *et al.* [106]	2017	Quantitative Descriptive Study	HAI Prevention	United States	HIC	ImD
Quinones Cardona *et al.* [107]	2021	Non-Randomized Trial	Sepsis Prevention	United States	HIC	ImS
Radbone *et al.* [108]	2013	Qualitative Study	NEC Prevention	United Kingdom	HIC	ImD/ImS
Rai *et al.* [109]	2021	Non-Randomized Trial	Hand Hygiene	India	LMIC	ImD/ImS
Ramos Ferreira Curan *et al.* [110]	2017	Mixed Methods Study	CLABSI/CRBSI Prevention	Brazil	LMIC	ImS
Rogers *et al.* [111]	2021	Non-Randomized Trial	Skin Integrity	United States	HIC	ImD/ImS

Authors	Year	Study Design	IPC Practice/Area	Country	Income Level	Content
Rohsiswatmo *et al.* [112]	2014	Mixed Methods Study	BSI Prevention	Indonesia	LMIC	ImS
Rojas Beytía *et al.* [113]	2020	Qualitative Study	Human Milk	Chile	HIC	ImS
Rolnitsky *et al.* [114]	2019	Non-Randomized Trial	Probiotics	Canada	HIC	ImS
Rosenthal *et al.* [115]	2013	Non-Randomized Trial	CLABSI/CRBSI Prevention	Multiple	LMIC	ImS
Rosenthal *et al.* [116]	2013	Non-Randomized Trial	Hand Hygiene	Multiple	LMIC	ImS
Ruch-Ross *et al.* [117]	2014	Quantitative Descriptive Study	Pandemic Prevention	United States	HIC	ImD
Sabry & Ibrahim [118]	2021	Non-Randomized Trial	Sepsis Prevention	Egypt	LMIC	ImS
Sadeghi-Moghaddam *et al.* [119]	2015	Non-Randomized Trial	Hand Hygiene	Iran	LMIC	ImS
Salem & Youssef [120]	2017	Qualitative Study	IPC, unspecified	Egypt	LMIC	ImD/ImS
Saporito *et al.* [121]	2021	Non-Randomized Trial	HAI Prevention	Italy	HIC	ImS
Shepherd *et al.* [122]	2015	Non-Randomized Trial	CLABSI/CRBSI Prevention	United States	HIC	ImD/ImS
Shettigar *et al.* [123]	2021	Non-Randomized Trial	HAI Prevention	India	LMIC	ImD/ImS
Short [124]	2019	Non-Randomized Trial	CLABSI/CRBSI Prevention	United States	HIC	ImS
Simen-Kapeu *et al.* [125]	2015	Mixed Methods Study	IPC, unspecified	Multiple	LMIC	ImD
Singh *et al.* [126]	2021	Non-Randomized Trial	Hand Hygiene	India	LMIC	ImD/ImS
Sinha *et al.* [127]	2016	Non-Randomized Trial	CLABSI/CRBSI Prevention	United Kingdom	HIC	ImD/ImS

**Authors**	**Year**	**Study Design**	**IPC Practice/Area**	**Country**	**Income Level**	**Content**
Somasekhara Aradhya *et al.* [128]	2022	Non-Randomized Trial	Environmental Cleaning	India	LMIC	ImD/ImS
Song *et al.* [129]	2013	Non-Randomized Trial	Hand Hygiene	United States	HIC	ImD/ImS
Steiner *et al.* [130]	2015	Non-Randomized Trial	CLABSI/CRBSI Prevention	n/a	n/a	ImD/ImS
Stone *et al.* [131]	2016	Qualitative Study	Human Milk	United States	HIC	ImD/ImS
Stroever *et al.* 2020 [132]	2020	Qualitative Study	CLABSI/CRBSI Prevention	United States	HIC	ImD/ImS
Sunkwa-Mills *et al.* [133]	2020	Qualitative Study	HAI Prevention	Ghana	LMIC	ImD/ImS
Szél *et al.* [134]	2017	Non-Randomized Trial	Outbreak Containment	Hungary	HIC	ImS
Taryana *et al.* [135]	2019	Non-Randomized Trial	Hand Hygiene	Indonesia	LMIC	ImS
Taylor *et al.* [136]	2017	Non-Randomized Trial	CLABSI/CRBSI Prevention	Australia	HIC	ImS
Thakur *et al.* [137]	2022	Non-Randomized Trial	Outbreak Containment	India	LMIC	ImS
Theron *et al.* [138]	2022	Qualitative Study	HAI Prevention	South Africa	LMIC	ImD
Thomas *et al.* [139]	2019	Non-Randomized Trial	Hand Hygiene	India	LMIC	ImD/ImS
Ting *et al.* [140]	2013	Non-Randomized Trial	CLABSI/CRBSI Prevention	Canada	HIC	ImS
Tran *et al.* [141]	2018	Non-Randomized Trial	IPC, unspecified	Viet Nam	LMIC	ImS
Triantafillou *et al.* [142]	2020	Qualitative Study	HAI Prevention	Greece	HIC	ImD
Trudel *et al.* [143]	2018	Qualitative Study	HAI Prevention	Canada	HIC	ImD/ImS

Authors	Year	Study Design	IPC Practice/Area	Country	Income Level	Content
Tsiatsiou *et al.* [144]	2015	Non-Randomized Trial	Outbreak Containment	Greece	HIC	ImS
Umulisa *et al.* [145]	2016	Non-Randomized Trial	Hand Hygiene	Rwanda	LMIC	ImD/ImS
Van Rostenberghe *et al.* [146]	2014	Non-Randomized Trial	IPC, unspecified	Malaysia	LMIC	ImS
Verma *et al.* [147]	2017	Randomized Controlled Trial	HAI Prevention	India	LMIC	ImS
Villegas Sánchez *et al.* [148]	2014	Non-Randomized Trial	CLABSI/CRBSI Prevention	n/a	n/a	ImS
Weber [149]	2016	Non-Randomized Trial	VAP Prevention	United States	HIC	ImD/ImS
Weiss *et al.* [150]	2021	Qualitative Study	NEC Prevention	United States	HIC	ImD/ImS
Wilder *et al.* [151]	2016	Non-Randomized Trial	CLABSI/CRBSI Prevention	United States	HIC	ImD/ImS
Yawson & Hesse [152]	2013	Non-Randomized Trial	Hand Hygiene	Ghana	LMIC	ImD
Zachariah *et al.* [153]	2014	Quantitative Descriptive Study	CLABSI/CRBSI Prevention	United States	HIC	ImS
Zhou & Chen [154]	2022	Non-Randomized Trial	HAI Prevention	China	LMIC	ImD
Zhou *et al.* [155]	2015	Non-Randomized Trial	CLABSI/CRBSI Prevention	China	LMIC	ImS
Zhou *et al.* [156]	2020	Quantitative Descriptive Study	Human Milk	China	LMIC	ImS

Implementation Determinants

	Barriers	Facilitators
	**Outer Setting**	
Critical Incidents	Barrett et al. 2023<br>Jain et al. 2023<br>Manerkar et al. 2022<br>Maria et al. 2022<br>Triantafillou et al. 2020	
External Pressure		Wilder et al. 2016
Financing	Simen-Kapeu et al. 2015	
Local Attitudes	Cowden et al. 2020<br>López et al. 2013<br>Morgan et al. 2018<br>Simen-Kapeu et al. 2015<br>Sunkwa-Mills et al. 2020<br>Triantafillou et al. 2020	
Local Conditions	Herbeć et al. 2020<br>Oko et al. 2022<br>Simen-Kapeu et al. 2015<br>Sunkwa-Mills et al. 2020<br>Triantafillou et al. 2020	Beekman & Steward 2020
Partnerships & Connections	Simen-Kapeu et al. 2015	Dawczynski et al. 2017<br>Dumpa et al. 2019<br>Dye et al. 2021<br>Erdei et al. 2015<br>Gephart et al. 2019<br>Hawes & Lee 2018<br>Pletsch et al. 2013
Policies & Laws	Anderson 2016<br>Simen-Kapeu et al. 2015<br>Triantafillou et al. 2020	Gephart et al. 2019<br>Maria et al. 2022
	**Inner Setting**	
**Access to Knowledge & Information**	Alimohammadzadeh et al. 2017	Alslaim et al. 2022

	Barriers	Facilitators
	Amaan et al. 2022	Azmeraw Getie et al. 2022
	Anderson 2016	Bierlaire et al. 2021
	Aragona et al. 2021	Cowden et al. 2020
	Badparva et al. 2023	Dramowski et al. 2021
	Batthula et al. 2021	Erdei et al. 2015
	Ceballos et al. 2013	Fisher et al. 2013
	Cross et al. 2016	Hanley et al. 2022
	Deshommes et al. 2021	Hawes & Lee 2018
	Dye et al. 2021	Herbeć et al. 2020
	EsquéRuiz et al. 2015	López et al. 2013
	Gephart et al. 2021	Manzo et al. 2022
	Gon et al. 2021	Maria et al. 2022
	Goodchild et al. 2018	Morgan et al. 2018
	Hanley et al. 2022	Ngugi et al. 2019
	Hawes & Lee 2018	Pahwa et al. 2018
	Hayashi et al. 2021	Radbone et al. 2013
	Herbeć et al. 2020	Ruch-Ross et al. 2014
	Jacobs Pepin et al. 2019	Sinha et al. 2016
	Kumar et al. 2022	Stone et al. 2016
	Manerkar et al. 2022	Zhou & Chen 2022
	Manzo et al. 2022	
	Maria et al. 2022	
	Morgan et al. 2018	
	Pahwa et al. 2018	
	Pasricha et al. 2021	
	Rai et al. 2021	
	Rogers et al. 2021	
	Rojas Beytía et al. 2020	
	Salem & Youssef 2017	
	Simen-Kapeu et al. 2015	
	Song et al. 2013	
	Steiner et al. 2015	
	Stroever et al. 2020	
	Sunkwa-Mills et al. 2020	
	Theron et al. 2022	
	Thomas et al. 2019	

	Barriers	Facilitators
	Triantafillou et al. 2020<br>Trudel et al. 2018<br>Weber 2016<br>Weiss et al. 2021<br>Wilder et al. 2016	
Available Resources	Alimohammadzadeh et al. 2017<br>Alrumi et al. 2020<br>Amaan et al. 2022<br>Azmeraw Getie et al. 2022<br>Badparva et al. 2023<br>Balla et al. 2018<br>Barrett et al. 2023<br>Batthula et al. 2021<br>Bharadwaj et al. 2019<br>Biswas et al. 2019<br>Cantey et al. 2013<br>Ceballos et al. 2013<br>Chandonnet et al. 2013<br>Cowden et al. 2020<br>Dawczynski et al. 2017<br>Deshommes et al. 2021<br>Dramowski et al. 2021<br>Erdei et al. 2015<br>Fischer Fumeaux et al. 2017<br>Gafirimbi et al. 2016<br>Gon et al. 2017<br>Gon et al. 2021<br>Hanley et al. 2022<br>Hawes & Lee 2018<br>Herbeć et al. 2020<br>Hightower et al. 2022<br>Kumar et al. 2022<br>Lauderbaugh et al. 2019<br>López et al. 2013<br>Manzo et al. 2022<br>Maria et al. 2022	Barrett et al. 2023<br>Gafirimbi et al. 2016<br>Gon et al. 2021<br>Maria et al. 2022<br>Ngugi et al. 2019<br>Oko et al. 2022<br>Pasricha et al. 2021<br>Rojas Beytía et al. 2020<br>Sinha et al. 2016<br>Yawson & Hesse 2013

	Barriers	Facilitators
	Morgan et al. 2018<br>Ngugi et al. 2019<br>Oko et al. 2022<br>Pasricha et al. 2021<br>Rai et al. 2021<br>Salem & Youssef 2017<br>Singh et al. 2021<br>Song et al. 2013<br>Sunkwa-Mills et al. 2020<br>Theron et al. 2022<br>Thomas et al. 2019<br>Triantafillou et al. 2020<br>Trudel et al. 2018<br>Weiss et al. 2021<br>Yawson & Hesse 2013	
Communication	Ceballos et al. 2013<br>Chandonnet et al. 2013<br>Cross et al. 2016<br>Dye et al. 2021<br>EsquéRuiz et al. 2015<br>Hanley et al. 2022<br>Herbeć et al. 2020<br>Jain et al. 2023<br>Madrid-Aguilar et al. 2019<br>Manerkar et al. 2022<br>Maria et al. 2022<br>Sunkwa-Mills et al. 2020<br>Triantafillou et al. 2020<br>Trudel et al. 2018<br>Wilder et al. 2016	Ceballos et al. 2013<br>Erdei et al. 2015<br>Hanley et al. 2022<br>Hawes & Lee 2018<br>Herbeć et al. 2020<br>Pahwa et al. 2018<br>Stone et al. 2016<br>Stroever et al. 2020<br>Sunkwa-Mills et al. 2020<br>Weiss et al. 2021
Compatibility	Barrett et al. 2023<br>Batthula et al. 2021<br>Biswas et al. 2019<br>Chandonnet et al. 2013<br>Cowden et al. 2020<br>Delaney Manthe et al. 2019	Aragona et al. 2021<br>Barrett et al. 2023<br>Maria et al. 2022<br>Morgan et al. 2018<br>Stone et al. 2016

	Barriers	Facilitators
	Fischer Fumeaux et al. 2017<br>Gon et al. 2021<br>Hanley et al. 2022<br>Herbeć et al. 2020<br>Jain et al. 2023<br>Manerkar et al. 2022<br>Maria et al. 2022<br>Morgan et al. 2018<br>Trudel et al. 2018<br>Weiss et al. 2021	
Culture	Bierlaire et al. 2021<br>Cowden et al. 2020<br>Herbeć et al. 2020<br>Triantafillou et al. 2020<br>Weiss et al. 2021	Dye et al. 2021<br>Erdei et al. 2015<br>Herbeć et al. 2020<br>Profit et al. 2017<br>Sunkwa-Mills et al. 2020<br>Weiss et al. 2021
Incentive Systems	Biswas et al. 2019<br>Cross et al. 2019<br>Dye et al. 2021<br>Gephart et al. 2021<br>Kumar et al. 2022<br>Salem & Youssef 2017<br>Sunkwa-Mills et al. 2020<br>Weiss et al. 2021	Dye et al. 2021<br>Erdei et al. 2015
Mission Alignment	Delaney Manthe et al. 2019	Dye et al. 2021<br>Erdei et al. 2015<br>Hawes & Lee 2018<br>Sunkwa-Mills et al. 2020<br>Zhou & Chen 2022
Relational Connections	EsquéRuiz et al. 2015<br>Jain et al. 2023<br>Madrid-Aguilar et al. 2019<br>Maria et al. 2022<br>Morgan et al. 2018	Azmeraw Getie et al. 2022<br>Barrett et al. 2023<br>Bezerra et al. 2021<br>Chandonnet et al. 2017<br>Dumpa et al. 2019<br>Erdei et al. 2015<br>Fisher et al. 2013

	**Barriers**	**Facilitators**
		Hanley et al. 2022<br>Hawes & Lee 2018<br>Herbeć et al. 2020<br>Maria et al. 2022<br>Morgan et al. 2018<br>Profit et al. 2017<br>Stone et al. 2016<br>Stroever et al. 2020<br>Wilder et al. 2016
**Relative Priority**	Badparva et al. 2023<br>Cowden et al. 2020<br>Kumar et al. 2022<br>Song et al. 2013<br>Triantafillou et al. 2020<br>Wilder et al. 2016	Chandonnet et al. 2017<br>Hightower et al. 2022<br>López et al. 2013<br>Maria et al. 2022<br>Pharande et al. 2018<br>Pletsch et al. 2013<br>Sunkwa-Mills et al. 2020
**Structural Characteristics**	Alimohammadzadeh et al. 2017<br>Alrumi et al. 2020<br>Amaan et al. 2022<br>Badparva et al. 2023<br>Balla et al. 2018<br>Barrett et al. 2023<br>Batthula et al. 2021<br>Bharadwaj et al. 2019<br>Bierlaire et al. 2021<br>Biswas et al. 2019<br>Caspari et al. 2017<br>Ceballos et al. 2013<br>Chandonnet et al. 2013<br>Cowden et al. 2020<br>Cross et al. 2016<br>Cross et al. 2019<br>Dawczynski et al. 2017<br>Delaney Manthe et al. 2019<br>Deshommes et al. 2021<br>Dye et al. 2021	Azmeraw Getie et al. 2022<br>Chandonnet et al. 2017<br>Gephart et al. 2021<br>Hanley et al. 2022<br>Herbeć et al. 2020<br>Mahieu et al. 2022<br>Maria et al. 2022<br>Pletsch et al. 2013<br>Rai et al. 2021<br>Rojas Beytía et al. 2020<br>Stroever et al. 2020<br>Sunkwa-Mills et al. 2020<br>Yawson & Hesse 2013<br>Zhou & Chen 2022

	Barriers	Facilitators
	EsquéRuiz et al. 2015<br>Gephart et al. 2021<br>Gon et al. 2017<br>Gon et al. 2021<br>Hanley et al. 2022<br>Hawes & Lee 2018<br>Hayashi et al. 2021<br>Herbeć et al. 2020<br>Hightower et al. 2022<br>Jain et al. 2023<br>Kallam et al. 2018<br>Kumar et al. 2022<br>Lauderbaugh et al. 2019<br>López et al. 2013<br>Madrid-Aguilar et al. 2019<br>Mahieu et al. 2022<br>Manerkar et al. 2022<br>Manzo et al. 2022<br>Maria et al. 2022<br>Morgan et al. 2018<br>Mukerji et al. 2013<br>Oko et al. 2022<br>Pasricha et al. 2021<br>Rai et al. 2021<br>Rogers et al. 2021<br>Rojas Beytía et al. 2020<br>Salem & Youssef 2017<br>Simen-Kapeu et al. 2015<br>Singh et al. 2021<br>Song et al. 2013<br>Stroever et al. 2020<br>Sunkwa-Mills et al. 2020<br>Theron et al. 2022<br>Thomas et al. 2019<br>Triantafillou et al. 2020<br>Trudel et al. 2018	

	Barriers	Facilitators
	Weiss et al. 2021<br>Wilder et al. 2016<br>Yawson & Hesse 2013	
Tension for Change		Chandonnet et al. 2017<br>Erdei et al. 2015<br>Herbeć et al. 2020<br>Howard-Jones et al. 2022<br>López et al. 2013<br>Weber 2016
	**Innovation**	
Capability	Alimohammadzadeh et al. 2017<br>Alslaim et al. 2022<br>Amaan et al. 2022<br>Anderson 2016<br>Badparva et al. 2023<br>Balla et al. 2018<br>Barrett et al. 2023<br>Batthula et al. 2021<br>Bharadwaj et al. 2019<br>Biswas et al. 2019<br>Ceballos et al. 2013<br>Chandonnet et al. 2013<br>Cowden et al. 2020<br>Cross et al. 2016<br>Cross et al. 2019<br>Dye et al. 2021<br>EsquéRuiz et al. 2015<br>Fabbri et al. 2013<br>Gafirimbi et al. 2016<br>Gon et al. 2017<br>Gon et al. 2021<br>Hawes & Lee 2018<br>Hayashi et al. 2021<br>Herbeć et al. 2020<br>Jain et al. 2023	Anderson 2016<br>Azmeraw Getie et al. 2022<br>Batthula et al. 2021<br>Delaney Manthe et al. 2019<br>Dramowski et al. 2021<br>Gon et al. 2017<br>Zhou & Chen 2022

	Barriers	Facilitators
	Kumar et al. 2022	
	Lauderbaugh et al. 2019	
	López et al. 2013	
	Manerkar et al. 2022	
	Maria et al. 2022	
	McCord et al. 2019	
	Németh et al. 2022	
	Ngugi et al. 2019	
	Pasricha et al. 2021	
	Rai et al. 2021	
	Rogers et al. 2021	
	Singh et al. 2021	
	Song et al. 2013	
	Stroever et al. 2020	
	Sunkwa-Mills et al. 2020	
	Theron et al. 2022	
	Triantafillou et al. 2020	
	Trudel et al. 2018	
	Weiss et al. 2021	
Motivation	Amaan et al. 2022	Bezerra et al. 2021
	Aragona et al. 2021	Ceballos et al. 2013
	Balla et al. 2018	Chandonnet et al. 2013
	Batthula et al. 2021	Chandonnet et al. 2017
	Bharadwaj et al. 2019	Cross et al. 2016
	Biswas et al. 2019	Dramowski et al. 2021
	Chandonnet et al. 2013	Fischer Fumeaux et al. 2017
	Cross et al. 2019	Gephart et al. 2021
	Fischer Fumeaux et al. 2017	Herbeć et al. 2020
	Hanley et al. 2022	Maria et al. 2022
	Herbeć et al. 2020	Pletsch et al. 2013
	Hightower et al. 2022	Rai et al. 2021
	Jain et al. 2023	Rojas Beytía et al. 2020
	Kumar et al. 2022	Ruch-Ross et al. 2014
	López et al. 2013	Stone et al. 2016
	Madrid-Aguilar et al. 2019	Weiss et al. 2021
	Manerkar et al. 2022	Zhou & Chen 2022

	**Barriers**	**Facilitators**
	Maria et al. 2022<br>Morgan et al. 2018<br>Pahwa et al. 2018<br>Pletsch et al. 2013<br>Rojas Beytía et al. 2020<br>Shettigar et al. 2021<br>Singh et al. 2021<br>Song et al. 2013	
Need	Bharadwaj et al. 2019<br>Hanley et al. 2022<br>Hightower et al. 2022<br>Manerkar et al. 2022<br>Mukerji et al. 2013<br>Rogers et al. 2021<br>Stroever et al. 2020	Zhou & Chen 2022
Opportunity	Biswas et al. 2019<br>Herbeć et al. 2020<br>Jain et al. 2023<br>Manerkar et al. 2022<br>Maria et al. 2022<br>Morgan et al. 2018<br>Pahwa et al. 2018	Gephart et al. 2019<br>Sunkwa-Mills et al. 2020
	**Innovation**	
**Innovation Adaptability**	Batthula et al. 2021<br>Hanley et al. 2022	Batthula et al. 2021<br>Morgan et al. 2018
**Innovation Complexity**	Hanley et al. 2022<br>Maria et al. 2022	Delaney Manthe et al. 2019
**Innovation Costs**	Kallam et al. 2018<br>Shettigar et al. 2021	López et al. 2013<br>Morgan et al. 2018<br>Pletsch et al. 2013<br>Somasekhara Aradhya et al. 2022<br>Umulisa et al. 2016
**Innovation Evidence Base**	Anderson 2016<br>Beekman & Steward 2020<br>Bowen et al. 2017	Anderson 2016<br>Cowden et al. 2020<br>Maria et al. 2022

	Barriers	Facilitators
	Jacobs Pepin et al. 2019<br>McCord et al. 2019<br>Pletsch et al. 2013<br>Weiss et al. 2021	
**Innovation Relative Advantage**		Cowden et al. 2020<br>Somasekhara Aradhya et al. 2022
**Innovation Source**	Anderson 2016	Bierlaire et al. 2021<br>Hanley et al. 2022

Implementation Strategies

Adapt and tailor to context

Promote adaptability
Batthula et al. 2021
Bechmann et al. 2023
Biswas et al. 2019
Dramowski et al. 2021
Fischer Fumeaux et al. 2017
Fisher et al. 2013
Gafirimbi et al. 2016
Gon et al. 2021
Hanley et al. 2022
Hawes & Lee 2018
Hightower et al. 2022
Johnson et al. 2022
Manerkar et al. 2022
Parga et al. 2017
Weiss et al. 2021

Tailor strategies
Alimohammadzadeh et al. 2017
Alshaikh et al. 2015
Balachander et al. 2020
Barrett et al. 2023
Bowen et al. 2017
Ceballos et al. 2013
Chandonnet et al. 2017
Dye et al. 2021
Erdei et al. 2015
Gafirimbi et al. 2016
Hawes & Lee 2018
Jacobs Pepin et al. 2019
Johnson et al. 2022
Kallam et al. 2018
Manerkar et al. 2022
Maria et al. 2022
Marofi et al. 2017
Mwananyanda et al. 2019
Rai et al. 2021
Singh et al. 2021
Trudel et al. 2018
Umulisa et al. 2016
Zhou et al. 2020

Use data warehousing techniques
Dye et al. 2021
Rosenthal et al. 2013

Change Infrastructure

Change physical structure and equipment

Almeida et al. 2017
Alslaim et al. 2022
Arena et al. 2013
Balla et al. 2018
Barrett et al. 2023
Bharadwaj et al. 2019
Bierlaire et al. 2021
Bowen et al. 2017
Caspari et al. 2017
Ceballos et al. 2013
Chandonnet et al. 2013
Chandonnet et al. 2017
Cowden et al. 2020
Delaney Manthe et al. 2019
Deshommes et al. 2021
Dramowski et al. 2021
Dumpa et al. 2019
Erdei et al. 2015
EsquéRuiz et al. 2015
Fabbri et al. 2013
Ferry et al. 2020
Goodchild et al. 2018
Hamza et al. 2022
Hawes & Lee 2018
Hightower et al. 2022
Howard-Jones et al. 2022
Johnson et al. 2022
Kallam et al. 2018
Khurana et al. 2018
Kumar et al. 2022
Lauderbaugh et al. 2019
Lee et al. 2015
López et al. 2013
Maria et al. 2022
Marom et al. 2020
Mwananyanda et al. 2019
Pahwa et al. 2018
Pasricha et al. 2021
Patel et al. 2014
Phan et al. 2020
Pharande et al. 2018
Pletsch et al. 2013
Prashantha et al. 2019
Rai et al. 2021
Rosenthal et al. 2013
Shepherd et al. 2015
Shettigar et al. 2021
Singh et al. 2021
Somasekhara Aradhya et al. 2022
Song et al. 2013
Szél et al. 2017
Taryana et al. 2019
Thomas et al. 2019
Ting et al. 2013
Tsiatsiou et al. 2015
Umulisa et al. 2016
Zhou et al. 2020

Change record systems

Barrett et al. 2023
Bowen et al. 2017
Ceballos et al. 2013
Chandonnet et al. 2013
Delaney Manthe et al. 2019
Dye et al. 2021
Erdei et al. 2015
Fisher et al. 2013
García González et al. 2017
Gephart et al. 2021
Hayashi et al. 2021
Jain et al. 2023
Linam et al. 2019
Madrid-Aguilar et al. 2019
Manerkar et al. 2022
Pallotto et al. 2017
Piazza et al. 2016
Pletsch et al. 2013
Rogers et al. 2021
Rohsiswatmo et al. 2014
Rolnitsky et al. 2019
Sinha et al. 2016

Change service sites

Maria et al. 2022
Sinha et al. 2016
Song et al. 2013
Zhou et al. 2015

Create accreditation or membership requirements

Bharadwaj et al. 2019
Chandonnet et al. 2013
Mukerji et al. 2013
Pharande et al. 2018
Shettigar et al. 2021
Steiner et al. 2015
Tran et al. 2018
Zhou et al. 2015

Create or change credentialing and/or licensure standards
Alslaim et al. 2022
Azab et al. 2015
Balla et al. 2018
Batthula et al. 2021
Ceballos et al. 2013
Dramowski et al. 2021
Gephart et al. 2021
Hawes & Lee 2018
Hensel et al. 2017
Hightower et al. 2022
Kallam et al. 2018
Lauderbaugh et al. 2019
Wilder et al. 2016

Mandate change
Beekman & Steward 2020
Chandonnet et al. 2013
Dye et al. 2021
Gephart et al. 2021
Tran et al. 2018
Zachariah et al. 2014

Develop relationships

Build a coalition
Alrumi et al. 2020
Barrett et al. 2023
Cantey et al. 2013
Erdei et al. 2015
Hanley et al. 2022
López et al. 2013
Radbone et al. 2013

Capture and share local knowledge
Grover et al. 2015
Lee et al. 2015
Pallotto et al. 2017
Piazza et al. 2016
Zhou et al. 2020

Conduct local consensus discussions
Gafirimbi et al. 2016
Lee et al. 2015
Radbone et al. 2013
Ramos Ferreira Curan et al. 2017
Rolnitsky et al. 2019
Thakur et al. 2022

Develop academic partnerships
Marofi et al. 2017

Identify and prepare champions
Bharadwaj et al. 2019
Bowen et al. 2017
Ceballos et al. 2013
Dumpa et al. 2019
Dye et al. 2021
Erdei et al. 2015
Gon et al. 2021
Hayashi et al. 2021
Johnson et al. 2022
Kallam et al. 2018
Kumar et al. 2022
Madrid-Aguilar et al. 2019
Pharande et al. 2018
Prashantha et al. 2019
Quinones Cardona et al. 2021
Radbone et al. 2013
Tran et al. 2018
Weiss et al. 2021
Zhou et al. 2020

Involve executive boards
Barrett et al. 2023
Bowen et al. 2017
Delaney Manthe et al. 2019
Dye et al. 2021
Fisher et al. 2013
Howard-Jones et al. 2022
Hussain et al. 2021
Johnson et al. 2022
Kallam et al. 2018
Maria et al. 2022
Neill et al. 2016
Pharande et al. 2018
Radbone et al. 2013
Ramos Ferreira Curan et al. 2017
Rosenthal et al. 2013
Shepherd et al. 2015
Wilder et al. 2016

Model and simulate change
Hightower et al. 2022
Steiner et al. 2015

Organize clinician implementation meetings
Barrett et al. 2023
Bowen et al. 2017
Ceballos et al. 2013
Dumpa et al. 2019
Dye et al. 2021
Grover et al. 2015
Hawes & Lee 2018
Howard-Jones et al. 2022
Kallam et al. 2018
Neill et al. 2016
Pletsch et al. 2013
Ramos Ferreira Curan et al. 2017
Shepherd et al. 2015
Singh et al. 2021
Ting et al. 2013
Weiss et al. 2021
Wilder et al. 2016

Promote network weaving
Bowen et al. 2017
Chandonnet et al. 2017
Dumpa et al. 2019
Dye et al. 2021
Erdei et al. 2015
EsquéRuiz et al. 2015
Fisher et al. 2013
Grover et al. 2015
Hawes & Lee 2018

Hightower et al. 2022
Jacobs Pepin et al. 2019
Johnson et al. 2022
Pletsch et al. 2013
Radbone et al. 2013
Rolnitsky et al. 2019
Rosenthal et al. 2013
Shepherd et al. 2015
Taylor et al. 2017
Wilder et al. 2016

Promote/Lobby for interests
López et al. 2013

Recruit, designate, and train for leadership
Gephart et al. 2021
Hussain et al. 2021

Use advisory boards and workgroups
Alimohammadzadeh et al. 2017
Alshaikh et al. 2015
Balla et al. 2018
Barrett et al. 2023
Batthula et al. 2021
Bechmann et al. 2023
Bharadwaj et al. 2019
Cantey et al. 2013
Ceballos et al. 2013
Chandonnet et al. 2013
Delaney Manthe et al. 2019
Dye et al. 2021
Erdei et al. 2015
EsquéRuiz et al. 2015
Fernández-Prada et al. 2019
Fisher et al. 2013
Gafirimbi et al. 2016
Gephart et al. 2021
Goodchild et al. 2018
Hamza et al. 2022
Hanley et al. 2022
Hawes & Lee 2018
Hayashi et al. 2021
Howard-Jones et al. 2022
Jacobs Pepin et al. 2019
Johnson et al. 2022
Kallam et al. 2018
Kumar et al. 2022
Lauderbaugh et al. 2019
Lee et al. 2015
Linam et al. 2019
López et al. 2013
Manerkar et al. 2022
Maria et al. 2022
Neill et al. 2016
Pahwa et al. 2018
Patel et al. 2014
Pharande et al. 2018
Piazza et al. 2016
Radbone et al. 2013
Rai et al. 2021
Ramos Ferreira Curan et al. 2017
Rogers et al. 2021
Rolnitsky et al. 2019
Shettigar et al. 2021
Singh et al. 2021
Somasekhara Aradhya et al. 2022
Szél et al. 2017
Ting et al. 2013
Tran et al. 2018
Tsiatsiou et al. 2015
Wilder et al. 2016
Zhou et al. 2020

Use an implementation advisor
Howard-Jones et al. 2022
Maria et al. 2022
Marom et al. 2020
Pahwa et al. 2018
Pallotto et al. 2017
Piazza et al. 2016
Singh et al. 2021

Visit other sites
Dye et al. 2021
Gafirimbi et al. 2016
Pharande et al. 2018
Zhou et al. 2020

Engage Individuals

Involve patients/consumers and family members

Chandonnet et al. 2013
Dye et al. 2021
EsquéRuiz et al. 2015
Fernández-Prada et al. 2019
Fisher et al. 2013
Goodchild et al. 2018
Hawes & Lee 2018
Jain et al. 2023
Johnson et al. 2022
Maria et al. 2022
Marom et al. 2020
Neill et al. 2016
Pletsch et al. 2013
Ramos Ferreira Curan et al. 2017
Short 2019
Singh et al. 2021
Song et al. 2013
Zhou et al. 2020

Prepare patients/consumers to be active participants

Erdei et al. 2015

Use mass media

Ceballos et al. 2013
EsquéRuiz et al. 2015
Gephart et al. 2021
Gon et al. 2021
Johnson et al. 2022
Lauderbaugh et al. 2019
Maria et al. 2022
Pallotto et al. 2017
Piazza et al. 2016
Radbone et al. 2013
Wilder et al. 2016
Zhou et al. 2020

Provide interactive assistance

<u>Centralize technical assistance</u>
Bharadwaj et al. 2019
Dye et al. 2021
Manerkar et al. 2022
Piazza et al. 2016

<u>Facilitation</u>
Batthula et al. 2021
Ceballos et al. 2013
Chandonnet et al. 2013
Gephart et al. 2021
Jain et al. 2023
Manerkar et al. 2022
Maria et al. 2022
Umulisa et al. 2016
Weiss et al. 2021

<u>Provide clinical supervision</u>
Balla et al. 2018
Biswas et al. 2019
Chandonnet et al. 2017
Gon et al. 2021
Grover et al. 2015
Tran et al. 2018
Umulisa et al. 2016

<u>Provide local technical assistance</u>
Balla et al. 2018
Ceballos et al. 2013
Delaney Manthe et al. 2019
Deshommes et al. 2021
Dye et al. 2021
Erdei et al. 2015
Fischer Fumeaux et al. 2017
Gephart et al. 2019
Hawes & Lee 2018
Hayashi et al. 2021
Hussain et al. 2021
Lee et al. 2015
López et al. 2013
Manerkar et al. 2022
Maria et al. 2022
Radbone et al. 2013
Sinha et al. 2016
Sunkwa-Mills et al. 2020
Tran et al. 2018
Zhou et al. 2015
Zhou et al. 2020

Support Individuals

Create new clinical teams

Bierlaire et al. 2021
Dye et al. 2021
Erdei et al. 2015
Ramos Ferreira Curan et al. 2017
Wilder et al. 2016
Zhou et al. 2015

Develop resource sharing agreements

Dye et al. 2021
Pletsch et al. 2013

Facilitate relay of clinical data to providers

Balla et al. 2018
Dye et al. 2021
Erdei et al. 2015
Grover et al. 2015
Hawes & Lee 2018
Hussain et al. 2021
Manerkar et al. 2022
Quinones Cardona et al. 2021

Hire new or additional staff

Hensel et al. 2017
Howard-Jones et al. 2022
Maria et al. 2022
Shepherd et al. 2015

Remind clinicians or other involved groups

Alslaim et al. 2022
Balla et al. 2018
Bharadwaj et al. 2019
Bowen et al. 2017
Ceballos et al. 2013
Chandonnet et al. 2017
Cowden et al. 2020
Deshommes et al. 2021
Dramowski et al. 2021
Ferry et al. 2020
Gephart et al. 2019
Goodchild et al. 2018
Hawes & Lee 2018
Hayashi et al. 2021
Hightower et al. 2022
Hussain et al. 2021
Johnson et al. 2022
Kallam et al. 2018
Kumar et al. 2022
Lauderbaugh et al. 2019
Linam et al. 2019
Mwananyanda et al. 2019
Ramos Ferreira Curan et al. 2017
Rosenthal et al. 2013
Shettigar et al. 2021
Somasekhara Aradhya et al. 2022
Thomas et al. 2019
Ting et al. 2013
Umulisa et al. 2016
Wilder et al. 2016
Zhou et al. 2015

Revise professional roles

Balla et al. 2018
Chandonnet et al. 2013
Erdei et al. 2015
Gafirimbi et al. 2016
Hawes & Lee 2018
Hussain et al. 2021
Johnson et al. 2022
Khurana et al. 2018
Maria et al. 2022
Prashantha et al. 2019
Stone et al. 2016
Ting et al. 2013
Umulisa et al. 2016
Wilder et al. 2016

Train and educate

Conduct educational meetings or provide trainings

Almeida et al. 2017
Alrumi et al. 2020
Alshaikh et al. 2015
Amaan et al. 2022
Arena et al. 2013
Azab et al. 2015
Barrett et al. 2023
Batthula et al. 2021
Bechmann et al. 2023
Bharadwaj et al. 2019
Bierlaire et al. 2021
Biswas et al. 2019
Bowen et al. 2017
Cantey et al. 2013
Caspari et al. 2017
Ceballos et al. 2013
Chandonnet et al. 2013
Chandonnet et al. 2017
Delaney Manthe et al. 2019
Deshommes et al. 2021
Dramowski et al. 2021
Dumpa et al. 2019
Dye et al. 2021
Erdei et al. 2015
EsquéRuiz et al. 2015
Fernández-Prada et al. 2019
Fisher et al. 2013
Gafirimbi et al. 2016
García González et al. 2017
Gephart et al. 2019
Gephart et al. 2021
Giuffrè et al. 2013
Goodchild et al. 2018
Grover et al. 2015
Hamza et al. 2022
Hanley et al. 2022
Hensel et al. 2017
Hightower et al. 2022
Howard-Jones et al. 2022
Jacobs Pepin et al. 2019
Jahan et al. 2022
Jain et al. 2023
Johnson et al. 2022
Kallam et al. 2018
Karabay et al. 2019
Khurana et al. 2018
Kumar et al. 2022
Lauderbaugh et al. 2019
Linam et al. 2019
López et al. 2013
Madrid-Aguilar et al. 2019
Manerkar et al. 2022
Maria et al. 2022
Marom et al. 2020
Mukerji et al. 2013
Mwananyanda et al. 2019
Neill et al. 2016
Pallotto et al. 2017
Parga et al. 2017
Pasricha et al. 2021
Patel et al. 2014
Phan et al. 2018
Phan et al. 2020
Pharande et al. 2018
Pletsch et al. 2013
Prashantha et al. 2019
Quinones Cardona et al. 2021
Radbone et al. 2013
Rai et al. 2021
Rogers et al. 2021
Rolnitsky et al. 2019
Rosenthal et al. 2013
Sabry & Ibrahim 2021
Sadeghi-Moghaddam et al. 2015
Shettigar et al. 2021
Singh et al. 2021
Song et al. 2013
Steiner et al. 2015
Stone et al. 2016
Szél et al. 2017
Thakur et al. 2022
Thomas et al. 2019
Ting et al. 2013
Tran et al. 2018
Tsiatsiou et al. 2015
Umulisa et al. 2016
Van Rostenberghe et al. 2014
Verma et al. 2017
VillegasSánchez et al. 2014
Weber 2016
Weiss et al. 2021
Wilder et al. 2016
Zhou et al. 2015
Zhou et al. 2020

Conduct educational outreach visits

Bowen et al. 2017
Delaney Manthe et al. 2019

Pahwa et al. 2018
Prashantha et al. 2019
Zhou et al. 2015
Zhou et al. 2020

Conduct ongoing training
Balla et al. 2018
Bharadwaj et al. 2019
Cantey et al. 2013
Ceballos et al. 2013
Delaney Manthe et al. 2019
Dramowski et al. 2021
Dye et al. 2021
EsquéRuiz et al. 2015
Geraci et al. 2014
Grover et al. 2015
Howard-Jones et al. 2022
Jacobs Pepin et al. 2019
Mukerji et al. 2013
Neill et al. 2016
Németh et al. 2022
Pletsch et al. 2013
Rohsiswatmo et al. 2014
Rosenthal et al. 2013
Shettigar et al. 2021
Stone et al. 2016
Stroever et al. 2020
Sunkwa-Mills et al. 2020
Taylor et al. 2017
Van Rostenberghe et al. 2014
Weber 2016
Zhou et al. 2015

Create a learning collaborative
Bowen et al. 2017
Fisher et al. 2013
Gephart et al. 2021
Grover et al. 2015
Hightower et al. 2022
Pallotto et al. 2017
Piazza et al. 2016
Stone et al. 2016

Develop educational materials
Almeida et al. 2017
Alshaikh et al. 2015
Batthula et al. 2021
Bowen et al. 2017
Cantey et al. 2013
Ceballos et al. 2013
Chandonnet et al. 2013
Chandonnet et al. 2017
Delaney Manthe et al. 2019
EsquéRuiz et al. 2015
Fernández-Prada et al. 2019
Fisher et al. 2013
Gafirimbi et al. 2016
García González et al. 2017
Gephart et al. 2021
Geraci et al. 2014
Goodchild et al. 2018
Grover et al. 2015
Hanley et al. 2022
Hawes & Lee 2018
Hightower et al. 2022
Howard-Jones et al. 2022
Jacobs Pepin et al. 2019
Johnson et al. 2022
Kallam et al. 2018
Lauderbaugh et al. 2019
Lee et al. 2015
López et al. 2013
Manerkar et al. 2022
Maria et al. 2022
Marofi et al. 2017
McCord et al. 2019
Mukerji et al. 2013
Neill et al. 2016
Pettit et al. 2017
Phan et al. 2020
Pharande et al. 2018
Prashantha et al. 2019
Radbone et al. 2013
Ramos Ferreira Curan et al. 2017
Rogers et al. 2021
Rohsiswatmo et al. 2014
Rolnitsky et al. 2019
Shettigar et al. 2021
Short 2019
Singh et al. 2021
Steiner et al. 2015
Szél et al. 2017
Tran et al. 2018
Van Rostenberghe et al. 2014
Verma et al. 2017
Zhou et al. 2020

Distribute educational materials
Alshaikh et al. 2015
Alslaim et al. 2022
Amaan et al. 2022

Balla et al. 2018
Barrett et al. 2023
Batthula et al. 2021
Bharadwaj et al. 2019
Bowen et al. 2017
Cantey et al. 2013
Caspari et al. 2017
Ceballos et al. 2013
Chandonnet et al. 2013
Chandonnet et al. 2017
Delaney Manthe et al. 2019
Erdei et al. 2015
EsquéRuiz et al. 2015
Fernández-Prada et al. 2019
Ferry et al. 2020
Fisher et al. 2013
Gafirimbi et al. 2016
García González et al. 2017
Gephart et al. 2021
Gon et al. 2017
Goodchild et al. 2018
Gopalakrishnan et al. 2021
Grover et al. 2015
Hanley et al. 2022
Hawes & Lee 2018
Hayashi et al. 2021
Howard-Jones et al. 2022
Jacobs Pepin et al. 2019
Johnson et al. 2022
Khurana et al. 2018
Kumar et al. 2022
Lee et al. 2015
Linam et al. 2019
López et al. 2013
Manerkar et al. 2022
Maria et al. 2022
Marom et al. 2020
Morgan et al. 2018
Mukerji et al. 2013
Mwananyanda et al. 2019
Neill et al. 2016
Parga et al. 2017
Pasricha et al. 2021
Patel et al. 2014
Pettit et al. 2017
Pharande et al. 2018
Pletsch et al. 2013
Prashantha et al. 2019
Rai et al. 2021
Ramos Ferreira Curan et al. 2017
Rolnitsky et al. 2019
Rosenthal et al. 2013
Sabry & Ibrahim 2021
Salem & Youssef 2017
Saporito et al. 2021
Shettigar et al. 2021
Singh et al. 2021
Sunkwa-Mills et al. 2020
Szél et al. 2017
Thomas et al. 2019
Ting et al. 2013
Zhou et al. 2020

Make training dynamic

Alrumi et al. 2020
Alshaikh et al. 2015
Amaan et al. 2022
Balla et al. 2018
Batthula et al. 2021
Bowen et al. 2017
Chandonnet et al. 2017
Delaney Manthe et al. 2019
Dye et al. 2021
Fisher et al. 2013
Gephart et al. 2021
Gopalakrishnan et al. 2021
Grover et al. 2015
Hawes & Lee 2018
Hightower et al. 2022
Jacobs Pepin et al. 2019
Jahan et al. 2022
Johnson et al. 2022
Manerkar et al. 2022
Maria et al. 2022
Mwananyanda et al. 2019
Németh et al. 2022
Phan et al. 2018
Phan et al. 2020
Pletsch et al. 2013
Ramos Ferreira Curan et al. 2017
Shettigar et al. 2021
Szél et al. 2017
Tran et al. 2018
Van Rostenberghe et al. 2014
Verma et al. 2017
Weiss et al. 2021

Provide ongoing consultation

Balachander et al. 2020
Dye et al. 2021
Johnson et al. 2022
Maria et al. 2022

Morgan et al. 2018
Rosenthal et al. 2013

Update educational materials
Barrett et al. 2023
Batthula et al. 2021
Bharadwaj et al. 2019
Hayashi et al. 2021
Maria et al. 2022

Sinha et al. 2016

Use train-the-trainer strategies
Alshaikh et al. 2015
Dramowski et al. 2021
Dye et al. 2021
Hightower et al. 2022
Khurana et al. 2018
Lee et al. 2015

Manerkar et al. 2022
Sinha et al. 2016
Van Rostenberghe et al. 2014
Verma et al. 2017
Zhou et al. 2015
Zhou et al. 2020

Use evaluative and iterative strategies

<u>Audit and provide feedback</u>
Alrumi et al. 2020
Alslaim et al. 2022
Balachander et al. 2020
Balla et al. 2018
Barrett et al. 2023
Bharadwaj et al. 2019
Bierlaire et al. 2021
Biswas et al. 2019
Bowen et al. 2017
Ceballos et al. 2013
Chandonnet et al. 2017
Dramowski et al. 2021
Dye et al. 2021
Erdei et al. 2015
Fernández-Prada et al. 2019
Gopalakrishnan et al. 2021
Hamza et al. 2022
Hawes & Lee 2018
Hussain et al. 2021
Jain et al. 2023
Johnson et al. 2022
Kallam et al. 2018
Karabay et al. 2019
Khurana et al. 2018
Lauderbaugh et al. 2019
Lee et al. 2015
Linam et al. 2019
Madrid-Aguilar et al. 2019
Maria et al. 2022
Marom et al. 2020
Neill et al. 2016
Phan et al. 2020
Pharande et al. 2018
Radbone et al. 2013
Rai et al. 2021
Ramos Ferreira Curan et al. 2017
Rosenthal et al. 2013
Saporito et al. 2021
Shepherd et al. 2015
Shettigar et al. 2021
Sinha et al. 2016
Somasekhara Aradhya et al. 2022
Stone et al. 2016
Szél et al. 2017
Taylor et al. 2017
Ting et al. 2013
Van Rostenberghe et al. 2014
Weber 2016
Wilder et al. 2016

<u>Conduct cyclical small test of change</u>
Alshaikh et al. 2015
Balla et al. 2018
Barrett et al. 2023
Batthula et al. 2021
Bharadwaj et al. 2019
Bierlaire et al. 2021
Ceballos et al. 2013
Grover et al. 2015
Hayashi et al. 2021
Hightower et al. 2022
Jain et al. 2023
Khurana et al. 2018
Kumar et al. 2022
Linam et al. 2019
Manerkar et al. 2022
Pahwa et al. 2018
Patel et al. 2014
Piazza et al. 2016
Pletsch et al. 2013
Quinones Cardona et al. 2021
Rai et al. 2021
Rolnitsky et al. 2019
Shettigar et al. 2021
Singh et al. 2021
Somasekhara Aradhya et al. 2022
Stone et al. 2016
Ting et al. 2013

<u>Conduct local needs assessment</u>
Alimohammadzadeh et al. 2017
Azab et al. 2015
Balla et al. 2018
Barrett et al. 2023
Batthula et al. 2021
Bharadwaj et al. 2019
Bierlaire et al. 2021
Biswas et al. 2019
Ceballos et al. 2013
Chandonnet et al. 2013
Dye et al. 2021
Erdei et al. 2015
EsquéRuiz et al. 2015
Gafirimbi et al. 2016
Gon et al. 2021
Goodchild et al. 2018
Grover et al. 2015
Hawes & Lee 2018

Hayashi et al. 2021
Jacobs Pepin et al. 2019
Jain et al. 2023
Johnson et al. 2022
Kallam et al. 2018
Khurana et al. 2018
Kumar et al. 2022
Linam et al. 2019
López et al. 2013
Manerkar et al. 2022
Németh et al. 2022
Patel et al. 2014
Prashantha et al. 2019
Rai et al. 2021
Ramos Ferreira Curan et al. 2017
Rogers et al. 2021
Shettigar et al. 2021
Somasekhara Aradhya et al. 2022
Song et al. 2013
Steiner et al. 2015
Ting et al. 2013
Weber 2016
Wilder et al. 2016
Zhou et al. 2020

Develop a formal implementation blueprint

Chandonnet et al. 2013
Chandonnet et al. 2017
Delaney Manthe et al. 2019
Fisher et al. 2013
Gafirimbi et al. 2016
Grover et al. 2015
Manerkar et al. 2022
Piazza et al. 2016
Prashantha et al. 2019
Somasekhara Aradhya et al. 2022

Develop and implement tools for quality monitoring

Barrett et al. 2023
Hawes & Lee 2018
Khurana et al. 2018
Maria et al. 2022
Pahwa et al. 2018
Patel et al. 2014
Ramos Ferreira Curan et al. 2017

Develop and organize quality monitoring systems

Alshaikh et al. 2015
Arena et al. 2013
Balachander et al. 2020
Balla et al. 2018
Barrett et al. 2023
Bechmann et al. 2023
Bharadwaj et al. 2019
Bierlaire et al. 2021
Bowen et al. 2017
Cantey et al. 2013
Caspari et al. 2017
Ceballos et al. 2013
Chandonnet et al. 2013
Chandonnet et al. 2017
Delaney Manthe et al. 2019
Dramowski et al. 2021
Dye et al. 2021
Erdei et al. 2015
Fabbri et al. 2013
Gajic et al. 2021
García González et al. 2017
Gephart et al. 2019
Goodchild et al. 2018
Gopalakrishnan et al. 2021
Hawes & Lee 2018
Hayashi et al. 2021
Howard-Jones et al. 2022
Hussain et al. 2021
Jahan et al. 2022
Jain et al. 2023
Johnson et al. 2022
Kallam et al. 2018
Karabay et al. 2019
Khurana et al. 2018
Kumar et al. 2022
Linam et al. 2019
López et al. 2013
Mahieu et al. 2022
Maria et al. 2022
Németh et al. 2022
Pallotto et al. 2017
Piazza et al. 2016
Prashantha et al. 2019
Rohsiswatmo et al. 2014
Rosenthal et al. 2013
Sabry & Ibrahim 2021
Salem & Youssef 2017
Shepherd et al. 2015
Somasekhara Aradhya et al. 2022
Szél et al. 2017
Taryana et al. 2019
Taylor et al. 2017
Ting et al. 2013
Tran et al. 2018
Tsiatsiou et al. 2015

Wilder et al. 2016
Zhou et al. 2015

Obtain and use patients/consumers and family feedback
Alimohammadzadeh et al. 2017
Barrett et al. 2023
Batthula et al. 2021
Bharadwaj et al. 2019
Caspari et al. 2017
Ceballos et al. 2013
Delaney Manthe et al. 2019
Dramowski et al. 2021
EsquéRuiz et al. 2015
Fabbri et al. 2013
Gafirimbi et al. 2016
Gephart et al. 2021
Goodchild et al. 2018
Hawes & Lee 2018
Khurana et al. 2018
Manerkar et al. 2022
Maria et al. 2022
Patel et al. 2014
Prashantha et al. 2019
Radbone et al. 2013
Ramos Ferreira Curan et al. 2017
Shettigar et al. 2021
Ting et al. 2013
Tran et al. 2018
Trudel et al. 2018

Purposefully reexamine the implementation
Alrumi et al. 2020
Balachander et al. 2020
Balla et al. 2018
Barrett et al. 2023
Batthula et al. 2021
Bharadwaj et al. 2019
Biswas et al. 2019
Ceballos et al. 2013
Chandonnet et al. 2017
Dumpa et al. 2019
Dye et al. 2021
Erdei et al. 2015
Fabbri et al. 2013
Fernández-Prada et al. 2019
Grover et al. 2015
Hawes & Lee 2018
Hightower et al. 2022
Howard-Jones et al. 2022
Jacobs Pepin et al. 2019
Jain et al. 2023
Johnson et al. 2022
Kallam et al. 2018
Maria et al. 2022
Neill et al. 2016
Pahwa et al. 2018
Pallotto et al. 2017
Patel et al. 2014
Quinones Cardona et al. 2021
Ramos Ferreira Curan et al. 2017
Rohsiswatmo et al. 2014
Rolnitsky et al. 2019
Sinha et al. 2016
Somasekhara Aradhya et al. 2022
Steiner et al. 2015
Tsiatsiou et al. 2015
Wilder et al. 2016

Stage implementation scale up
Alshaikh et al. 2015
Azab et al. 2015
Barrett et al. 2023
Caspari et al. 2017
Erdei et al. 2015
Gephart et al. 2021
Gon et al. 2021
Hamza et al. 2022
Jacobs Pepin et al. 2019
Linam et al. 2019
Madrid-Aguilar et al. 2019
Parga et al. 2017
Rolnitsky et al. 2019
Somasekhara Aradhya et al. 2022
Steiner et al. 2015
Thomas et al. 2019
Verma et al. 2017

Update tools/systems for quality monitoring
Almeida et al. 2017
Balla et al. 2018
Barrett et al. 2023
Chandonnet et al. 2013
Erdei et al. 2015
EsquéRuiz et al. 2015
Hayashi et al. 2021
Maria et al. 2022
Patel et al. 2014
Saporito et al. 2021
Szél et al. 2017
Ting et al. 2013
Weber 2016

Wilder et al. 2016

Utilize financial/incentivizing strategies

Access new funding
Dye et al. 2021
Sinha et al. 2016

Alter incentive/allowance structures
Bowen et al. 2017
Ceballos et al. 2013
Dye et al. 2021
Erdei et al. 2015
EsquéRuiz et al. 2015
Gephart et al. 2021
Goodchild et al. 2018
Gopalakrishnan et al. 2021
Hanley et al. 2022
Hawes & Lee 2018
Herbeć et al. 2020
Hussain et al. 2021
Johnson et al. 2022
Lauderbaugh et al. 2019
Maria et al. 2022
Marofi et al. 2017
Pharande et al. 2018
Rohsiswatmo et al. 2014
Shepherd et al. 2015
Van Rostenberghe et al. 2014
Wilder et al. 2016

Place innovation on fee for service lists/formularies
Delaney Manthe et al. 2019

References

1. Alimohammadzadeh, K., et al., *Assessing Common Medical Errors in a Children's Hospital NICU Using Failure Mode and Effects Analysis (FMEA).* Trauma Monthly, 2017. **22**(5): p. -.
2. Almeida, C.C., et al., *Nosocomial sepsis: evaluation of the efficacy of preventive measures in a level-III neonatal intensive care unit.* J Matern Fetal Neonatal Med, 2017. **30**(17): p. 2036-2041.
3. Alrumi, N., et al., *Infection control measures in neonatal units: implementation of change in the Gaza-Strip.* The Journal of Maternal-Fetal & Neonatal Medicine, 2020. **33**(20): p. 3490-3496.
4. Alshaikh, B., et al., *Effect of a Quality Improvement Project to Use Exclusive Mother's Own Milk on Rate of Necrotizing Enterocolitis in Preterm Infants.* Breastfeed Med, 2015. **10**(7): p. 355-61.
5. Alslaim, H.S., et al., *Discordance among Belief, Practice, and the Literature in Infection Prevention in the NICU.* Children, 2022. **9**(4): p. 492.
6. Amaan, A., S.K. Dey, and K. Zahan, *Improvement of Hand Hygiene Practices among the Healthcare Workers in a Neonatal Intensive Care Unit.* Can J Infect Dis Med Microbiol, 2022. **2022**: p. 7688778.
7. Anderson, S., *Barriers to Knowledge Translation Regarding the Use of Probiotics as a Risk-Reduction Strategy for Necrotizing Enterocolitis.* Adv Neonatal Care, 2016. **16**(4): p. E3-e14.
8. Aragona, E., D. West, and J. Loyal, *Well-Newborn Unit Director Experiences During the COVID-19 Pandemic: A BORN Study.* Hosp Pediatr, 2021. **11**(9): p. e170-e181.
9. Arena, F., et al., *Large oligoclonal outbreak due to Klebsiella pneumoniae ST14 and ST26 producing the FOX-7 AmpC β-lactamase in a neonatal intensive care unit.* J Clin Microbiol, 2013. **51**(12): p. 4067-72.
10. Azab, S.F., et al., *Reducing ventilator-associated pneumonia in neonatal intensive care unit using "VAP prevention Bundle": a cohort study.* BMC Infect Dis, 2015. **15**: p. 314.
11. Azmeraw Getie, B., A. Engida Yismaw, and A. Eskezia Tiguh, *Kangaroo mother care knowledge and practice among mothers who gave birth to preterm and low birth weight babies in Amhara regional state referral hospitals, North West Ethiopia.* International Journal of Africa Nursing Sciences, 2022. **17**: p. 100470.
12. Badparva, B., et al., *Prevention of central line-associated bloodstream infections: ICU nurses' knowledge and barriers.* Nurs Crit Care, 2023. **28**(3): p. 419-426.
13. Balachander, B., et al., *Response Measures to Infection Outbreaks During the Second Year of Sustenance Phase of Infection Control Quality Improvement.* The Indian Journal of Pediatrics, 2020. **87**(5): p. 333-338.
14. Balla, K.C., et al., *Decreasing Central Line-associated Bloodstream Infections Through Quality Improvement Initiative.* Indian Pediatr, 2018. **55**(9): p. 753-756.
15. Barrett, R.E., et al., *Reducing MRSA Infection in a New NICU During the COVID-19 Pandemic.* Pediatrics, 2023. **151**(2).
16. Batthula, V., S.H. Somnath, and V. Datta, *Reducing late-onset neonatal sepsis in very low birthweight neonates with central lines in a low-and-middle-income country setting.* BMJ Open Qual, 2021. **10**(Suppl 1).
17. Bechmann, L., et al., *Serratia marcescens outbreak in a neonatal intensive care unit associated with contaminated donor milk.* Infection Control & Hospital Epidemiology, 2023. **44**(6): p. 891-897.
18. Beekman, K. and D. Steward, *Chlorhexidine Gluconate Utilization for Infection Prevention in the NICU: A Survey of Current Practice.* Adv Neonatal Care, 2020. **20**(1): p. 38-47.
19. Bezerra, T.B., et al., *Influencing factors of hand hygiene in critical sections of a brazilian hospital.* J Infect Dev Ctries, 2021. **15**(6): p. 840-846.
20. Bharadwaj, S., et al., *Eliminating MRSA transmission in a tertiary neonatal unit-A quality improvement initiative.* Am J Infect Control, 2019. **47**(11): p. 1329-1335.
21. Bierlaire, S., et al., *How to minimize central line–associated bloodstream infections in a neonatal intensive care unit: a quality improvement intervention based on a retrospective analysis and the adoption of an evidence-based bundle.* European Journal of Pediatrics, 2021. **180**(2): p. 449-460.
22. Biswas, A., et al., *Addressing Hand Hygiene Compliance in a Low-Resource Neonatal Intensive Care Unit: a Quality Improvement Project.* J Pediatric Infect Dis Soc, 2019. **8**(5): p. 408-413.
23. Bowen, J.R., et al., *Decreasing infection in neonatal intensive care units through quality improvement.* Archives of Disease in Childhood - Fetal and Neonatal Edition, 2017. **102**(1): p. F51-F57.

24. Cantey, J.B., et al., *Prompt control of an outbreak caused by extended-spectrum β-lactamase-producing Klebsiella pneumoniae in a neonatal intensive care unit.* J Pediatr, 2013. **163**(3): p. 672-9.e1-3.
25. Caspari, L., et al., *Human factors related to time-dependent infection control measures: "Scrub the hub" for venous catheters and feeding tubes.* Am J Infect Control, 2017. **45**(6): p. 648-651.
26. Ceballos, K., et al., *Nurse-driven quality improvement interventions to reduce hospital-acquired infection in the NICU.* Adv Neonatal Care, 2013. **13**(3): p. 154-63; quiz 164-5.
27. Chandonnet, C.J., et al., *It's in Your Hands: An Educational Initiative to Improve Parent/Family Hand Hygiene Compliance.* Dimens Crit Care Nurs, 2017. **36**(6): p. 327-333.
28. Chandonnet, C.J., et al., *Health care failure mode and effect analysis to reduce NICU line-associated bloodstream infections.* Pediatrics, 2013. **131**(6): p. e1961-9.
29. Cowden, C., et al., *Healthcare worker perceptions of the implementation context surrounding an infection prevention intervention in a Zambian neonatal intensive care unit.* BMC Pediatrics, 2020. **20**(1): p. 432.
30. Cross, S., et al., *Hygiene on maternity units: lessons from a needs assessment in Bangladesh and India.* Global Health Action, 2016. **9**(1): p. 32541.
31. Cross, S., et al., *An invisible workforce: the neglected role of cleaners in patient safety on maternity units.* Global Health Action, 2019. **12**(1): p. 1480085.
32. Dawczynski, K., et al., *[Infection Prevention in Premature Infants and Newborns in Thuringia: Implementation of Recommendation of the Commission for Hospital Hygiene and Infection Prevention (KRINKO)].* Z Geburtshilfe Neonatol, 2017. **221**(1): p. 30-38.
33. Delaney Manthe, E., P.H. Perks, and J.R. Swanson, *Team-Based Implementation of an Exclusive Human Milk Diet.* Adv Neonatal Care, 2019. **19**(6): p. 460-467.
34. Deshommes, T., et al., *A Quality Improvement Initiative to Increase Hand Hygiene Awareness and Compliance in a Neonatal Intensive Care Unit in Haiti.* J Trop Pediatr, 2021. **67**(3).
35. Dramowski, A., et al., *NeoCLEAN: a multimodal strategy to enhance environmental cleaning in a resource-limited neonatal unit.* Antimicrobial Resistance & Infection Control, 2021. **10**(1): p. 35.
36. Dumpa, V., et al., *Reduction in Central Line-Associated Bloodstream Infection Rates After Implementations of Infection Control Measures at a Level 3 Neonatal Intensive Care Unit().* Am J Med Qual, 2019. **34**(5): p. 488-493.
37. Dye, M.E., et al., *Developing a Unit-Based Quality Improvement Program in a Large Neonatal ICU.* Jt Comm J Qual Patient Saf, 2021. **47**(10): p. 654-662.
38. Erdei, C., et al., *Is zero central line-associated bloodstream infection rate sustainable? A 5-year perspective.* Pediatrics, 2015. **135**(6): p. e1485-93.
39. Esqué Ruiz, M.T., et al., *Towards a safety culture in the neonatal unit: Six years experience.* Anales de Pediatría (English Edition), 2015. **83**(4): p. 236-243.
40. Fabbri, G., et al., *Outbreak of ampicillin/piperacillin-resistant Klebsiella pneumoniae in a neonatal intensive care unit (NICU): investigation and control measures.* Int J Environ Res Public Health, 2013. **10**(3): p. 808-15.
41. Fernández-Prada, M., et al., *Brote de Klebsiella pneumoniae productora de betalactamasas de espectro extendido en una unidad de cuidados intensivos neonatales: factores de riesgo y medidas de prevención clave para su erradicación en tiempo récord.* Anales de Pediatría, 2019. **91**(1): p. 13-20.
42. Ferry, A., et al., *Enterobacter cloacae colonisation and infection in a neonatal intensive care unit: retrospective investigation of preventive measures implemented after a multiclonal outbreak.* BMC Infectious Diseases, 2020. **20**(1): p. 682.
43. Fisher, D., et al., *Reducing central line-associated bloodstream infections in North Carolina NICUs.* Pediatrics, 2013. **132**(6): p. e1664-71.
44. Fischer Fumeaux, C.J., et al., *Early Use of Mother's Own Raw Milk, Maternal Satisfaction, and Breastfeeding Continuation in Hospitalised Neonates: A Prospective Cohort Study.* Neonatology, 2017. **113**(2): p. 131-139.
45. Gafirimbi, N., et al., *Lessons learned in establishing a quality improvement project to reduce hospital acquired infections in the neonatology ward at a referral hospital in Rwanda.* On the Horizon, 2016. **24**(4): p. 341-348.
46. Gajic, I., et al., *Clinical and molecular characteristics of OXA-72-producing Acinetobacter baumannii ST636 outbreak at a neonatal intensive care unit in Serbia.* Journal of Hospital Infection, 2021. **112**: p. 54-60.
47. García González, A., et al., *Cinco pasos para la disminución de las infecciones relacionadas con la asistencia sanitaria en prematuros grandes inmaduros. Estudio cuasiexperimental.* Anales de Pediatría, 2017. **87**(1): p. 26-33.

48. Gephart, S.M., et al., *Feasibility and Acceptability of a Neonatal Project ECHO (NeoECHO) as a Dissemination and Implementation Strategy to Prevent Necrotizing Enterocolitis.* Worldviews Evid Based Nurs, 2021. **18**(6): p. 361-370.
49. Gephart, S.M. and M.C. Quinn, *Relationship of Necrotizing Enterocolitis Rates to Adoption of Prevention Practices in US Neonatal Intensive Care Units.* Adv Neonatal Care, 2019. **19**(4): p. 321-332.
50. Geraci, D.M., et al., *Methicillin-resistant Staphylococcus aureus colonization: a three-year prospective study in a neonatal intensive care unit in Italy.* PLoS One, 2014. **9**(2): p. e87760.
51. Giuffrè, M., et al., *Outbreak of colonizations by extended-spectrum β-lactamase-producing Escherichia coli sequence type 131 in a neonatal intensive care unit, Italy.* Antimicrob Resist Infect Control, 2013. **2**(1): p. 8.
52. Gon, G., et al., *Unpacking the enabling factors for hand, cord and birth-surface hygiene in Zanzibar maternity units.* Health Policy Plan, 2017. **32**(8): p. 1220-1228.
53. Gon, G., et al., *The Clean pilot study: evaluation of an environmental hygiene intervention bundle in three Tanzanian hospitals.* Antimicrobial Resistance & Infection Control, 2021. **10**(1): p. 8.
54. Goodchild, L., et al., *Promoting early expression of breast milk in mothers of preterm infants in a neonatal unit: a best practice implementation project.* JBI Database System Rev Implement Rep, 2018. **16**(10): p. 2027-2037.
55. Gopalakrishnan, S., et al., *Stepwise interventions for improving hand hygiene compliance in a level 3 academic neonatal intensive care unit in north India.* Journal of Perinatology, 2021. **41**(12): p. 2834-2839.
56. Grover, T.R., et al., *Interdisciplinary teamwork and the power of a quality improvement collaborative in tertiary neonatal intensive care units.* J Perinat Neonatal Nurs, 2015. **29**(2): p. 179-86.
57. Hamza, W.S., et al., *A multidisciplinary intervention to reduce central line-associated bloodstream infection in pediatrics and neonatal intensive care units.* Pediatr Neonatol, 2022. **63**(1): p. 71-77.
58. Hanley, S.J., et al., *Implementation of Public Health England infection prevention and control guidance in maternity units in response to the COVID-19 pandemic.* Journal of Hospital Infection, 2022. **129**: p. 219-226.
59. Hawes, J.A. and K.S. Lee, *Reduction in Central Line-Associated Bloodstream Infections in a NICU: Practical Lessons for Its Achievement and Sustainability.* Neonatal Netw, 2018. **37**(2): p. 105-115.
60. Hayashi, M., et al., *Improving timeliness of hepatitis B vaccine administration in an urban safety net level III NICU.* BMJ Qual Saf, 2021. **30**(11): p. 911-919.
61. Hensel, K.O., et al., *Nursing staff fluctuation and pathogenic burden in the NICU - effective outbreak management and the underestimated relevance of non-resistant strains.* Scientific Reports, 2017. **7**(1): p. 45014.
62. Herbeć, A., et al., *Barriers and facilitators to infection prevention and control in a neonatal unit in Zimbabwe – a theory-driven qualitative study to inform design of a behaviour change intervention.* Journal of Hospital Infection, 2020. **106**(4): p. 804-811.
63. Hightower, H.B., et al., *Reduction of Central-line-Associated Bloodstream Infections in a Tertiary Neonatal Intensive Care Unit through Simulation Education.* Pediatr Qual Saf, 2022. **7**(6): p. e610.
64. Howard-Jones, A.R., et al., *Prompt control of a Serratia marcescens outbreak in a neonatal intensive care unit informed by whole-genome sequencing and comprehensive infection control intervention package.* Antimicrobial Stewardship & Healthcare Epidemiology, 2022. **2**(1): p. e104.
65. Hussain, A.S., et al., *CLABSI reduction using evidence based interventions and nurse empowerment: a quality improvement initiative from a tertiary care NICU in Pakistan.* Arch Dis Child, 2021. **106**(4): p. 394-400.
66. Jacobs Pepin, B., et al., *ZAP-VAP: A Quality Improvement Initiative to Decrease Ventilator-Associated Pneumonia in the Neonatal Intensive Care Unit, 2012-2016.* Adv Neonatal Care, 2019. **19**(4): p. 253-261.
67. Jahan, I., et al., *Effectiveness of Educational Intervention in Preventing Ventilator Associated Pneumonia in Neonatal Intensive Care Unit: A Cohort Study: Prevention of ventilator associated pneumonia.* Bangladesh Medical Research Council Bulletin, 2022. **47**(2): p. 143-150.

68. Jain, H., et al., *Quality improvement initiative approach to increase the duration of Kangaroo Mother Care in a neonatal intensive care unit of a tertiary care institute in South India during the COVID-19 pandemic.* Journal of Pediatric Nursing, 2023. **68**: p. 74-78.
69. Johnson, J., et al., *Implementation of the Comprehensive Unit-Based Safety Program to Improve Infection Prevention and Control Practices in Four Neonatal Intensive Care Units in Pune, India.* Frontiers in Pediatrics, 2022. **9**.
70. Kallam, B., et al., *Implementation science in low-resource settings: using the interactive systems framework to improve hand hygiene in a tertiary hospital in Ghana.* International Journal for Quality in Health Care, 2018. **30**(9): p. 724-730.
71. Karabay, M., et al., *Effect of camera monitoring and feedback along with training on hospital infection rate in a neonatal intensive care unit.* Annals of Clinical Microbiology and Antimicrobials, 2019. **18**(1): p. 35.
72. Khurana, S., et al., *Reducing Healthcare-associated Infections in Neonates by Standardizing and Improving Compliance to Aseptic Non-touch Techniques: A Quality Improvement Approach.* Indian Pediatr, 2018. **55**(9): p. 748-752.
73. Kumar, A., et al., *Improvement of Hand Hygiene Compliance Using the Plan-Do-Study-Act Method: Quality Improvement Project From a Tertiary Care Institute in Bihar, India.* Cureus, 2022. **14**(6): p. e25590.
74. Lauderbaugh, D., et al., *Reducing Ventilator Associated Pneumonia in the NICU through oral care education: A quality improvement project.* Journal of Neonatal Nursing, 2019. **25**(3): p. 127-129.
75. Lee, S.K., et al., *The Evidence-based Practice for Improving Quality method has greater impact on improvement of outcomes than dissemination of practice change guidelines and quality improvement training in neonatal intensive care units.* Paediatr Child Health, 2015. **20**(1): p. e1-9.
76. Linam, W.M., et al., *Focusing on Families and Visitors Reduces Healthcare Associated Respiratory Viral Infections in a Neonatal Intensive Care Unit.* Pediatr Qual Saf, 2019. **4**(6): p. e242.
77. López, S., et al., *Quality in practice: preventing and managing neonatal sepsis in Nicaragua.* Int J Qual Health Care, 2013. **25**(5): p. 599-605.
78. Madrid-Aguilar, M., et al., *Implementación de NeoKissEs en España: un sistema validado de vigilancia de la sepsis nosocomial en recién nacidos de muy bajo peso.* Anales de Pediatría, 2019. **91**(1): p. 3-12.
79. Mahieu, L., et al., *Compliance with international prevention guidelines for central-line-associated bloodstream infections in neonatal intensive care units in Belgium: a national survey.* J Hosp Infect, 2022. **129**: p. 49-57.
80. Manerkar, S., et al., *Improving Early Colostrum Feeding in a Tertiary Neonatal Intensive Care Unit: A Quality Improvement Initiative.* Breastfeed Med, 2022. **17**(2): p. 143-148.
81. Manzo, B.F., et al., *Knowledge and Practices for Central Line Infection Prevention Among Brazilian Nurses: A Mixed-Methods Study.* Adv Neonatal Care, 2022. **22**(2): p. 180-187.
82. Maria, A., et al., *Barriers and enablers of breastfeeding in mother-newborn dyads in institutional settings during the COVID-19 pandemic: A qualitative study across seven government hospitals of Delhi, India.* Front Nutr, 2022. **9**: p. 1052340.
83. Maria, A., et al., *Improving handwashing among parent-attendants visiting a newborn unit practising family participatory care.* BMJ Open Qual, 2022. **11**(Suppl 1).
84. Marofi, M., et al., *The Impact of an Educational Program Regarding Total Parenteral Nutrition on Infection Indicators in Neonates Admitted to the Neonatal Intensive Care Unit.* Iran J Nurs Midwifery Res, 2017. **22**(6): p. 486-489.
85. Marom, R., et al., *A silent outbreak of vancomycin-resistant Enterococcus faecium in a neonatal intensive care unit.* Antimicrobial Resistance & Infection Control, 2020. **9**(1): p. 87.
86. McCord, H., E. Fieldhouse, and W. El-Naggar, *Current Practices of Antiseptic Use in Canadian Neonatal Intensive Care Units.* Am J Perinatol, 2019. **36**(2): p. 141-147.
87. Morgan, M.C., et al., *Kangaroo mother care for clinically unstable neonates weighing ≤2000 g: Is it feasible at a hospital in Uganda?* J Glob Health, 2018. **8**(1): p. 010701.
88. Mukerji, A., et al., *An observational study of the hand hygiene initiative: a comparison of preintervention and postintervention outcomes.* BMJ Open, 2013. **3**(5): p. e003018.
89. Mwananyanda, L., et al., *Preventing Bloodstream Infections and Death in Zambian Neonates: Impact of a Low-cost Infection Control Bundle.* Clin Infect Dis, 2019. **69**(8): p. 1360-1367.
90. Neill, S., et al., *Sustained Reduction in Bloodstream Infections in Infants at a Large Tertiary Care Neonatal Intensive Care Unit.* Adv Neonatal Care, 2016. **16**(1): p. 52-9.
91. Németh, I.A.K., et al., *Establishing a Learning Model for Correct Hand Hygiene Technique in a NICU.* Journal of Clinical Medicine, 2022. **11**(15): p. 4276.

92. Ngugi, S.K., F.V. Murila, and R.N. Musoke, *Hand hygiene practices among healthcare workers in a newborn unit of a tertiary referral hospital in Kenya.* Journal of Infection Prevention, 2019. **20**(3): p. 132-138.
93. Oko, C., A. Yusuf, and F. Taib, *Hand Hygiene Compliance during COVID-19 Pandemic among Neonatal Nurses in a Federal University Teaching Hospital in Nigeria.* Malaysian Journal of Medicine and Health Sciences, 2022. **18**: p. 119-127.
94. Pahwa, P., S. Lunsford, and N. Livesley, *Experiences of Indian Health Workers Using WhatsApp for Improving Aseptic Practices With Newborns: Exploratory Qualitative Study.* JMIR Med Inform, 2018. **6**(1): p. e13.
95. Pallotto, E.K., et al., *Sustaining SLUG Bug CLABSI Reduction: Does Sterile Tubing Change Technique Really Work?* Pediatrics, 2017. **140**(4).
96. Parga, J.J., et al., *Handshake-free zone in a neonatal intensive care unit: Initial feasibility study.* American Journal of Infection Control, 2017. **45**(7): p. 787-792.
97. Pasricha, S., et al., *Neonatal intensive care unit hand hygiene: Exploring current practice and adherence barriers in a Canadian hospital.* Canadian Journal of Infection Control, 2021. **36**(2): p. 77-85.
98. Patel, A.L., et al., *Reducing necrotizing enterocolitis in very low birth weight infants using quality-improvement methods.* J Perinatol, 2014. **34**(11): p. 850-7.
99. Pettit, J.D. and E.L. Sharpe, *The Effect of Education on Chlorhexidine Use in a Neonatal Intensive Care Unit.* Journal of the Association for Vascular Access, 2017. **22**(3): p. 115-123.
100. Phan, H.T., et al., *An educational intervention to improve hand hygiene compliance in Vietnam.* BMC Infectious Diseases, 2018. **18**(1): p. 116.
101. Phan, H.T., et al., *Enhanced infection control interventions reduced catheter-related bloodstream infections in the neonatal department of Hung Vuong Hospital, Vietnam, 2011–2012: a pre- and post-intervention study.* Antimicrobial Resistance & Infection Control, 2020. **9**(1): p. 9.
102. Pharande, P., et al., *Trends in late-onset sepsis in a neonatal intensive care unit following implementation of infection control bundle: A 15-year audit.* J Paediatr Child Health, 2018. **54**(12): p. 1314-1320.
103. Piazza, A.J., et al., *SLUG Bug: Quality Improvement With Orchestrated Testing Leads to NICU CLABSI Reduction.* Pediatrics, 2016. **137**(1).
104. Pletsch, D., et al., *Mothers' "liquid gold": a quality improvement initiative to support early colostrum delivery via oral immune therapy (OIT) to premature and critically ill newborns.* Nurs Leadersh (Tor Ont), 2013. **26 Spec No 2013**: p. 34-42.
105. Prashantha, Y.N., et al., *Onsite mentoring of special newborn care unit to improve the quality of newborn care.* Indian J Public Health, 2019. **63**(4): p. 357-361.
106. Profit, J., et al., *Teamwork in the NICU Setting and Its Association with Health Care–Associated Infections in Very Low-Birth-Weight Infants.* Am J Perinatol, 2017. **34**(10): p. 1032-1040.
107. Quinones Cardona, V., et al., *Eliminating Contamination in Umbilical Cord Blood Culture Sampling for Early-Onset Neonatal Sepsis.* Frontiers in Pediatrics, 2021. **9**.
108. Radbone, L., J. Birch, and M. Upton, *The development and implementation of a care bundle aimed at reducing the incidence of NEC.* Infant, 2013. **9**(1): p. 14-19.
109. Rai, R., et al., *Quality Improvement Initiative to Improve Hand Hygiene Compliance in Indian Special Newborn Care Unit.* Pediatr Qual Saf, 2021. **6**(6): p. e492.
110. Ramos Ferreira Curan, G., E. Giovanini Rossetto, and T. Corrêa Castral, *Using the knowledge translation framework to change practical care of central catheters in a Brazilian neonatal unit.* J Infect Dev Ctries, 2017. **11**(6): p. 445-452.
111. Rogers, S., et al., *A Quality Improvement Approach to Perineal Skin Care: Using Standardized Guidelines and Novel Diaper Wipes to Reduce Diaper Dermatitis in NICU Infants.* Adv Neonatal Care, 2021. **21**(3): p. 189-197.
112. Rohsiswatmo, R., S. Rafika, and P.M. Marsubrin, *Prevention and control of blood stream infection using the balanced scorecard approach.* Acta Med Indones, 2014. **46**(3): p. 209-16.
113. Rojas Beytía, J.P., et al., *[Health professional's perception about the use of human colostrum, as preventive measure for necrotizing enterocolitis in preterm newborns].* Rev Chil Pediatr, 2020. **91**(4): p. 536-544.
114. Rolnitsky, A., et al., *A Quality Improvement Intervention to Reduce Necrotizing Enterocolitis in premature infants with Probiotic Supplementation.* Pediatr Qual Saf, 2019. **4**(5): p. e201.
115. Rosenthal, V.D., et al., *Findings of the International Nosocomial Infection Control Consortium (INICC), Part III Effectiveness of a Multidimensional Infection Control Approach to Reduce Central Line–Associated Bloodstream Infections in the Neonatal Intensive Care Units of 4 Developing Countries.* Infection Control & Hospital Epidemiology, 2013. **34**(3): p. 229-237.
116. Rosenthal, V.D., et al., *Impact of the International Nosocomial Infection Control Consortium (INICC) Multidimensional Hand Hygiene Approach over 13 Years in 51 Cities of 19 Limited-Resource Countries from Latin America, Asia, the Middle East, and Europe.* Infection Control & Hospital Epidemiology, 2013. **34**(4): p. 415-423.

117. Ruch-Ross, H.S., et al., *General influenza infection control policies and practices during the 2009 H1N1 influenza pandemic: A survey of women's health, obstetric, and neonatal nurses.* American Journal of Infection Control, 2014. **42**(6): p. e65-e70.
118. Sabry, N. and M.H. Ibrahim, *A new approach to managing neonates born to mothers at risk for early-onset neonatal sepsis: is it cost-effective and can it reduce NICU admissions?* Journal of Pediatric and Neonatal Individualized Medicine (JPNIM), 2021. **10**(1): p. e100122.
119. Sadeghi-Moghaddam, P., et al., *Does training improve compliance with hand hygiene and decrease infections in the neonatal intensive care unit? A prospective study.* J Neonatal Perinatal Med, 2015. **8**(3): p. 221-5.
120. Salem, M.R. and M.R.L. Youssef, *Health care providers' perspectives for providing quality infection control measures at the neonatal intensive care unit, Cairo University Hospital.* American Journal of Infection Control, 2017. **45**(9): p. e99-e102.
121. Saporito, L., et al., *Efficacy of a coordinated strategy for containment of multidrug-resistant Gram-negative bacteria carriage in a Neonatal Intensive Care Unit in the context of an active surveillance program.* Antimicrobial Resistance & Infection Control, 2021. **10**(1): p. 30.
122. Shepherd, E.G., et al., *Significant Reduction of Central-Line Associated Bloodstream Infections in a Network of Diverse Neonatal Nurseries.* The Journal of Pediatrics, 2015. **167**(1): p. 41-46.e3.
123. Shettigar, S., et al., *Reducing healthcare-associated infections by improving compliance to aseptic non-touch technique in intravenous line maintenance: a quality improvement approach.* BMJ Open Quality, 2021. **10**(Suppl 1): p. e001394.
124. Short, K.L., *Implementation of a Central Line Maintenance Bundle for Dislodgement and Infection Prevention in the NICU.* Adv Neonatal Care, 2019. **19**(2): p. 145-150.
125. Simen-Kapeu, A., et al., *Treatment of neonatal infections: a multi-country analysis of health system bottlenecks and potential solutions.* BMC Pregnancy and Childbirth, 2015. **15**(2): p. S6.
126. Singh, M., et al., *Supplementing hand washing with proper use of alcoholic hand rub in a special neonatal care unit in a large academic public health institute at Jabalpur, Madhya Pradesh, India.* BMJ Open Qual, 2021. **10**(4).
127. Sinha, A.K., et al., *Prevention of Late Onset Sepsis and Central-line Associated Blood Stream Infection in Preterm Infants.* Pediatr Infect Dis J, 2016. **35**(4): p. 401-6.
128. Somasekhara Aradhya, A., et al., *Reducing the costs of floor cleaning in a level III NICU of Bangalore rural: a quality.* BMJ Open Qual, 2022. **11**(Suppl 1).
129. Song, X., et al., *Improving hand hygiene compliance in health care workers: Strategies and impact on patient outcomes.* American Journal of Infection Control, 2013. **41**(10): p. e101-e105.
130. Steiner, M., et al., *Significant Reduction of Catheter-associated Blood Stream Infections in Preterm Neonates After Implementation of a Care Bundle Focusing on Simulation Training of Central Line Insertion.* Pediatr Infect Dis J, 2015. **34**(11): p. 1193-6.
131. Stone, S., H.C. Lee, and P.J. Sharek, *Perceived Factors Associated with Sustained Improvement Following Participation in a Multicenter Quality Improvement Collaborative.* Jt Comm J Qual Patient Saf, 2016. **42**(7): p. 309-15.
132. Stroever, S., et al., *Qualitative process evaluation of a central line-associated bloodstream infection (CLABSI) prevention team in the neonatal intensive care unit.* American Journal of Infection Control, 2020. **48**(9): p. 987-992.
133. Sunkwa-Mills, G., et al., *Perspectives and practices of healthcare providers and caregivers on healthcare-associated infections in the neonatal intensive care units of two hospitals in Ghana.* Health Policy and Planning, 2020. **35**(Supplement_1): p. i38-i50.
134. Szél, B., et al., *Successful elimination of extended-spectrum beta-lactamase (ESBL)-producing nosocomial bacteria at a neonatal intensive care unit.* World J Pediatr, 2017. **13**(3): p. 210-216.
135. Taryana, A., M. Sampurna, and G. Sari, *Compliance in Maintaining Hand Cleaning on Health Care Workers in Neonatology Unit in Tertiary Referral Hospital Indonesia: The Usage of CCTV for Supervision.* Indian Journal of Public Health Research & Development, 2019. **10**: p. 1188.
136. Taylor, J.E., et al., *A quality improvement initiative to reduce central line infection in neonates using checklists.* Eur J Pediatr, 2017. **176**(5): p. 639-646.
137. Thakur, A., et al., *Clinical Presentation, Investigation and Control of an Outbreak of Adenoviral Conjunctivitis in a Neonatal Unit at a Tertiary Hospital.* Pediatr Infect Dis J, 2022. **41**(3): p. 243-247.
138. Theron, M., Y. Botma, and T. Heyns, *Infection prevention and control practices of non-medical individuals in a neonatal intensive care unit: A Donabedian approach.* Midwifery, 2022. **112**: p. 103393.

139. Thomas, A.M., et al., *Effectiveness of hand hygiene promotional program based on the WHO multimodal hand hygiene improvement strategy, in terms of compliance and decontamination efficacy in an indian tertiary level neonatal surgical intensive care unit.* Indian J Med Microbiol, 2019. **37**(4): p. 496-501.
140. Ting, J.Y., V.S. Goh, and H. Osiovich, *Reduction of central line-associated bloodstream infection rates in a neonatal intensive care unit after implementation of a multidisciplinary evidence-based quality improvement collaborative: A four-year surveillance.* Can J Infect Dis Med Microbiol, 2013. **24**(4): p. 185-90.
141. Tran, H.T., et al., *Early Essential Newborn Care Is Associated With Reduced Adverse Neonatal Outcomes in a Tertiary Hospital in Da Nang, Viet Nam: A Pre- Post- Intervention Study.* EClinicalMedicine, 2018. **6**: p. 51-58.
142. Triantafillou, V., et al., *Influence of national culture and context on healthcare workers' perceptions of infection prevention in Greek neonatal intensive care units.* Journal of Hospital Infection, 2020. **104**(4): p. 552-559.
143. Trudel, C., et al., *Human factors considerations in designing for infection prevention and control in neonatal care – findings from a pre-design inquiry.* Ergonomics, 2018. **61**(1): p. 169-184.
144. Tsiatsiou, O., et al., *Successful management of an outbreak due to carbapenem-resistant Acinetobacter baumannii in a neonatal intensive care unit.* European Journal of Pediatrics, 2015. **174**(1): p. 65-74.
145. Umulisa, S., et al., *Improvement of hand hygiene compliance among health professional staff of Neonatology Department in Nyamata Hospital.* On the Horizon, 2016. **24**(4): p. 349-356.
146. Van Rostenberghe, H., et al., *A Psychologist-Led Educational Intervention Results in a Sustained Reduction in Neonatal Intensive Care Unit Infections.* Frontiers in Pediatrics, 2014. **2**.
147. Verma, A., et al., *Family-Centered Care to Complement Care of Sick Newborns: A Randomized Controlled Trial.* Indian Pediatr, 2017. **54**(6): p. 455-459.
148. Villegas Sánchez, M., M. Arias Jiménez, and M. Hernández de Mezerville, *Acciones educativas dirigidas al personal médico y de enfermería para disminuir las infecciones del tracto sanguíneo relacionadas a catéteres venosos centrales.* Enfermería Actual en Costa Rica, 2014. **0**(27).
149. Weber, C.D., *Applying Adult Ventilator-associated Pneumonia Bundle Evidence to the Ventilated Neonate.* Adv Neonatal Care, 2016. **16**(3): p. 178-90.
150. Weiss, A.B., et al., *Exploring Internal Facilitators' Experience With NeoECHO to Foster NEC Prevention and Timely Recognition Through the iPARIHS Lens.* Adv Neonatal Care, 2021. **21**(6): p. 462-472.
151. Wilder, K.A., et al., *CLABSI Reduction Strategy: A Systematic Central Line Quality Improvement Initiative Integrating Line-Rounding Principles and a Team Approach.* Adv Neonatal Care, 2016. **16**(3): p. 170-7.
152. Yawson, A.E. and A.A. Hesse, *Hand hygiene practices and resources in a teaching hospital in Ghana.* J Infect Dev Ctries, 2013. **7**(4): p. 338-47.
153. Zachariah, P., et al., *The Association of State Legal Mandates for Data Submission of Central Line–Associated Bloodstream Infections in Neonatal Intensive Care Units with Process and Outcome Measures.* Infection Control & Hospital Epidemiology, 2014. **35**(9): p. 1133-1139.
154. Zhou, J. and S. Chen, *Knowledge, Attitudes, and Practices of NICU Doctors and Nurses Toward Prevention and Control of Nosocomial Infection With Multidrug Resistant Organism.* Frontiers in Pediatrics, 2022. **10**.
155. Zhou, Q., et al., *Successful reduction in central line–associated bloodstream infections in a Chinese neonatal intensive care unit.* American Journal of Infection Control, 2015. **43**(3): p. 275-279.
156. Zhou, Q., et al., *A Quality Improvement Initiative to Increase Mother's Own Milk Use in a Chinese Neonatal Intensive Care Unit.* Breastfeed Med, 2020. **15**(4): p. 261-267.
