# Supplementary material for: Investigating the implementation of infection prevention and control practices in neonatal care across country income levels: a systematic review
Source: Antimicrob Resist Infect Control. 2025 Feb 7;14:8. doi: 10.1186/s13756-025-01516-7 (PMC11806577; doi:10.1186/s13756-025-01516-7)
Supplement: Supplementary file 2 — Additional File 2: Actors and Target Groups of extracted Implementation Strategies. [file 13756_2025_1516_MOESM2_ESM.pdf]

Investigating the Implementation of Infection Prevention and Control Practices in Neonatal Care Across Country Income Levels: A Systematic Review

Emanuela Nyantakyi, Julia Baenziger, Laura Caci, Kathrin Blum, Aline Wolfensberger, Angela Dramowski, Bianca Albers, Marta Castro, Marie-Therese Schultes, Lauren Clack

SUPPLEMENTARY FILE

Actors and Target Groups of extracted Implementation Strategies

Table of Contents

Adapt and tailor to context 3

Change Infrastructure 5

Develop Relationships 7

Engage Individuals 15

Provide Interactive Assistance 17

Support Individuals 20

Train and Educate 22

Use evaluative and iterative strategies 30

Utilize financial/incentivizing strategies 38

Values represent number of studies

Adapt and tailor to context

Actors	Advisory Group/ Expert Group	Authors/ Study Team	Healthcare Professionals, unspecified	Network/ Collaborative	Project Team/Task Force/ Work Group	QI Team	Risk Management (Team)	n/a
**Promote adaptability**	1		1		1	1		12
**Tailor strategies**		1			1	1	1	19
**Use data warehousing techniques**				1				1

Target Groups	Champions	Healthcare Professionals, unspecified	Mothers/ Parents/ Families/ Caregivers /Visitors	NICUs/Units	Nurses	QI Team	Patients/ Neonates	n/a
**Promote adaptability**	1	2	2	1	2			7

Values represent number of studies

Target Groups	Champions	Healthcare Professionals, unspecified	Mothers/ Parents/ Families/ Caregivers /Visitors	NICUs/Units	Nurses	QI Team	Patients/ Neonates	n/a
**Tailor strategies**		4	4	1	3	1	1	9
**Use data warehousing techniques**				1				1

Values represent number of studies

Change Infrastructure

Actors	Hospital, unspecified	IPC Team	Ministry of Health	Network/ Collaborative	NICUs/Unit	QI Team	State/Policy	Unit Leadership	n/a
**Change physical structure and equipment**						1			57
**Change record systems**				1		2			19
**Change service sites**	1				3				
**Create accreditation or membership requirements**									8
**Create or change credentialing and/or licensure standards**		1				2			10
**Mandate change**			1				3	2	

Values represent number of studies

Target Groups	Cleaning Staff	Doctors/Neonatologists	Healthcare Professionals, unspecified	Mothers/ Parents/ Families/Caregivers/Visitors	NICUs/Units	Nurses	QI Team	n/a
**Change physical structure and equipment**	1		1	4		1		52
**Change record systems**					1	2		20
**Change service sites**								4
**Create accreditation or membership requirements**		1	3				1	2
**Create or change credentialing and/or licensure standards**	1		8			2		1
**Mandate change**	1		1		3	1		2

Values represent number of studies

Develop Relationships

Actors	Advisory Group/ Expert Group	Champions	Facilitators	Hospital, unspecified	Hospital Administration	Healthcare Professionals, unspecified	IPC Team	IPC Doctors/ Nurses/Personnel	Project Team/Task Force/ Work Group	Ministry of Health	Multidisciplinary Team	Network Collaborative	NGOs/NPOs	NICUs/Units	Nurses
**Build a coalition**							1	1		1				1	
**Capture and share local knowledge**									2					3	
**Conduct local consensus discussions**			1						2						
**Develop academic partnerships**															
**Identify and prepare champions**		1													
**Involve executive boards**					1	1	1							1	
**Model and simulate change**														1	

Values represent number of studies

Actors	Advisory Group/ Expert Group	Champions	Facilitators	Hospital, unspecified	Hospital Administration	Healthcare Professionals, unspecified	IPC Team	IPC Doctors/ Nurses/Personnel	Project Team/Task Force/ Work Group	Ministry of Health	Multidisciplinary Team	Network Collaborative	NGOs/NPOs	NICUs/Units	Nurses
**Organize clinician implementation meetings**		1					1		4						
**Promote network weaving**				1		1	2					3		8	
**Promote/Lobby for interests**													1		
**Recruit, designate, and train for leadership**															
**Use advisory boards and workgroups**	3						3		9		5				
**Use an implementation advisor**									1						
**Visit other sites**														1	2

Values represent number of studies

Actors (cont'd)	Outbreak Management Team	Patient/Family (Advisory) Group	QI Leadership	QI Team	Risk Management Team	Unit Leadership	n/a
**Build a coalition**							4
**Capture and share local knowledge**				1			
**Conduct local consensus discussions**				2			1
**Develop academic partnerships**							1
**Identify and prepare champions**				2			16
**Involve executive boards**						1	14
**Model and simulate change**				1			2
**Organize clinician implementation meetings**			1	5		1	4

Values represent number of studies

Actors (cont'd)	Outbreak Management Team	Patient/Family (Advisory) Group	QI Leadership	QI Team	Risk Management Team	Unit Leadership	n/a
**Promote network weaving**				1			4
**Promote/Lobby for interests**							
**Recruit, designate, and train for leadership**						1	1
**Use advisory boards and workgroups**	3	2	1	25	1		3
**Use an implementation advisor**				3			3
**Visit other sites**				1			1

Values represent number of studies

Target Groups	Champions	Cleaning Staff	Conferences/Congresses	Doctors/Neonatologists	External Partners	Hospital Administration	Healthcare Professionals, unspecified	IPC Doctors/Nurses/ Personnel	IPC Team	Leadership (various)	Ministry of Health	Mothers/ Parents/ Families/Caregivers/ Visitors	Network/Collaborative
**Build a coalition**		1			1						1		
**Capture and share local knowledge**													
**Conduct local consensus discussions**							1						
**Develop academic partnerships**													
**Identify and prepare champions**	5			2		1	2						
**Involve executive boards**						7				4			
**Model and simulate change**							2						
**Organize clinician implementation meetings**							5					1	

Values represent number of studies

Target Groups	Champions	Cleaning Staff	Conferences/Congresses	Doctors/Neonatologists	External Partners	Hospital Administration	Healthcare Professionals, unspecified	IPC Doctors/Nurses/ Personnel	IPC Team	Leadership (various)	Ministry of Health	Mothers/ Parents/ Families/Caregivers/ Visitors	Network/Collaborative
**Promote network weaving**			1				1		1				12
**Promote/Lobby for interests**											1		
**Recruit, designate, and train for leadership**							1	1					
**Use advisory boards and workgroups**							1		1				
**Use an implementation advisor**											1		
**Visit other sites**											1		

Values represent number of studies

Target Groups (cont'd)	**NGOs/NPOs**	**NICUs/Units**	**Nurses**	Patient/Family (Advisory) Group	**Pharmacists**	**Project Team/Task Force/ Work Group**	**QI Nurses**	**QI Team**	**Senior HCPs**	**Unit Leadership**	**University/Higher Education Institution**	**n/a**
**Build a coalition**	1	2										1
**Capture and share local knowledge**		3	1			1		1				
**Conduct local consensus discussions**												5
**Develop academic partnerships**			5								1	
**Identify and prepare champions**					1		1					3
**Involve executive boards**									3	3		3
**Model and simulate change**												
**Organize clinician implementation meetings**		1										11

Values represent number of studies

Target Groups (cont'd)	NGOs/NPOs	NICUs/Units	Nurses	Patient/Family (Advisory) Group	Pharmacists	Project Team/Task Force/ Work Group	QI Nurses	QI Team	Senior HCPs	Unit Leadership	University/Higher Education Institution	n/a
**Promote network weaving**		3										3
**Promote/Lobby for interests**												
**Recruit, designate, and train for leadership**												
**Use advisory boards and workgroups**				1				5		1		44
**Use an implementation advisor**												6
**Visit other sites**												4

Values represent number of studies

Engage Individuals

Actors	Advisory Group/ Expert Group	Facilitators	Mothers/ Parents/ Families/ Caregivers/ Visitors	Nurses	QI Nurses	QI Team	n/a
Involve patients/consumers and family members	1	1	1	1		1	15
Prepare patients/consumers to be active participants							1
Use mass media					1		11

Values represent number of studies

Target Groups	Champions	Cleaning Staff	Healthcare Professionals, unspecified	Hospital Administration	Leadership (various)	Mothers/Parents/ Families/Caregivers/ Visitors	Nurses	Leadership (various)	Unit Leadership	Patient/Family (Advisory) Group	n/a
**Involve patients/consumers and family members**		1	6	1		12			1	1	1
**Prepare patients/consumers to be active participants**							1				
**Use mass media**	2		4		1	1	1	1			3

Values represent number of studies

Provide Interactive Assistance

Actors	Advisory Group/ Expert Group	Champions	Doctors/Neonatologists	Educator/Coach	Lactation Specialist/ Consultants/ Counselors	Legal Team	Facilitators	Healthcare Professionals, unspecified	IPC Doctors/Nurses/ Personnel	IPC Team	Midwives
**Centralize technical assistance**	1				1						
**Facilitation**			1				2	1			
**Provide clinical supervision**		1						1	1		
**Provide local technical assistance**	1	1	1	1	4	1			5	1	1

Values represent number of studies

Actors (cont'd)	Nurse	Nurse Educators	Nutritionists/Dietitians	Project Team/Task Force/ Work Group	Public Institution	QI Nurse	QI Team	Senior Healthcare Professionals, unspecified	Unit Leadership	n/a
**Centralize technical assistance**										2
**Facilitation**				1			2			1
**Provide clinical supervision**	1			1				1	1	1
**Provide local technical assistance**	1	1	1	1	1	2				2

Values represent number of studies

Target Groups	Authors/ Study Team	Champions	Healthcare Professionals, unspecified	Mothers/Parents/ Families/Caregivers/ Visitors	NICUs/Units	Nurses	Patients/Neonates	Project Team/Task Force/ Work Group	QI Team	Unit Leadership	n/a
**Centralize technical assistance**									1		3
**Facilitation**	2		5	2			1				2
**Provide clinical supervision**			2	2	1						2
**Provide local technical assistance**		1	2	6	1	1		1	2	1	9

Values represent number of studies

Support Individuals

Actors	Doctors/Neonatologists	Hospital Administration	IPC Team	Microbiologists	NICUs/Units	Nurse Leaders	Pharmacists	Authors/Study Team	QI Team	n/a
**Create new clinical teams**										6
**Develop resource sharing agreements**					1					
**Facilitate relay of clinical data to providers**				1						7
**Hire new or additional staff**		1								3
**Remind clinicians or other involved groups**	1					1	1	1	1	28
**Revise professional roles**			1							13

Values represent number of studies

Target Groups	Authors/Study Team	Cleaning Staff	Epidemiology Team	Healthcare Professionals, unspecified	IPC Team	Mothers/Parents/ Families/Caregivers/ Visitors	Network/Collaborative	Nutritionists/Dietitians	Nurses	Project Team/Task Force/ Work Group	QI Leadership	n/a
**Create new clinical teams**					3			1				3
**Develop resource sharing agreements**							2					
**Facilitate relay of clinical data to providers**	1			4					1	1		1
**Hire new or additional staff**			1	2					1			
**Remind clinicians or other involved groups**				20		3			1			10
**Revise professional roles**		1		2	1				8		1	2

Values represent number of studies

Train and Educate

Actors	Advisory Group/Expert Group	Authors/Study Team	Champions	Doctors/Neonatologists	Educators	Individual Experts	Facilitators	Healthcare Professionals, unspecified	IPC Doctors/Nurses/Personnel	IPC Team	Lactation Specialist/Consultant	Leadership, unspecified	Local Health Authority
**Conduct educational meetings or provide trainings**	1	1	1	3		1	1	2	3	6		1	
**Conduct educational outreach visits**	1					2							1
**Conduct ongoing training**				1				1		4			
**Create a learning collaborative**													
**Develop educational materials**		1		1									
**Distribute educational materials**										1	1		
**Make training dynamic**		1				1	1			1			

Values represent number of studies

Actors	Advisory Group/Expert Group	Authors/Study Team	Champions	Doctors/Neonatologists	Educators	Individual Experts	Facilitators	Healthcare Professionals, unspecified	IPC Doctors/Nurses/ Personnel	IPC Team	Lactation Specialist/Consultant	Leadership, unspecified	Local Health Authority
**Provide ongoing consultation**		1		1	1								
**Update educational materials**				1									
**Use train-the-trainer strategies**													

Actors (cont'd)	Microbiologist	Ministry pf Health	Network/ Collaborative	NICUs/Units	Nurses	Nurse Educators	Nutritionists/Dietitians	Other NICUs/Units	Parents/Families/Caregivers/Visitors	Professional Associations	Project Team/ Task Force/ Work Group	Public Institution
**Conduct educational meetings or provide trainings**	1		1		2	1	1		1		5	2

Values represent number of studies

Actors (cont'd)	Microbiologist	Ministry pf Health	Network/ Collaborative	NICUs/Units	Nurses	Nurse Educators	Nutritionists/Dietitians	Other NICUs/Units	Parents/Families/Caregivers/Visitors	Professional Associations	Project Team/ Task Force/ Work Group	Public Institution
**Conduct educational outreach visits**			1					1				
**Conduct ongoing training**					1	1	1					
**Create a learning collaborative**												
**Develop educational materials**		1		1	1		1			1	6	
**Distribute educational materials**		1	1	1							3	
**Make training dynamic**												1
**Provide ongoing consultation**			1									
**Update educational materials**										1	5	

Values represent number of studies

Actors (cont'd)	Microbiologist	Ministry pf Health	Network/ Collaborative	NICUs/Units	Nurses	Nurse Educators	Nutritionists/Dietitians	Other NICUs/Units	Parents/Families/Caregivers/Visitors	Professional Associations	Project Team/ Task Force/ Work Group	Public Institution
**Use train-the-trainer strategies**	1		1		2	1						2

Actors (cont'd)	QI Team	QI Leadership	Senior HCPs	Unit Leadership	n/a
**Conduct educational meetings or provide trainings**	3		1	1	77
**Conduct educational outreach visits**					
**Conduct ongoing training**	2				16

Values represent number of studies

Actors (cont'd)	QI Team	QI Leadership	Senior HCPs	Unit Leadership	n/a
**Create a learning collaborative**					8
**Develop educational materials**	4				40
**Distribute educational materials**	2				57
**Make training dynamic**	1				26
**Provide ongoing consultation**	1	1			
**Update educational materials**	1				4
**Use train-the-trainer strategies**	1	1			7

Values represent number of studies

Target Groups	Champions	Cleaning Staff	Doctors/Neonatologists	Facilitators	Healthcare Professionals, unspecified	IPC Team	Lactation Specialist/Consultant/Counselors	Mothers/Parents/ Families/Caregivers/ Visitors	Network/Collaborative	NICUs/Units	Nurses	Project Team/Task Force/ Work Group
**Conduct educational meetings or provide trainings**	2	2	2		61	4	1	15		1	11	2
**Conduct educational outreach visits**					1		1			1	1	
**Conduct ongoing training**		2			15	1		1			2	1
**Create a learning collaborative**										7		
**Develop educational materials**					11		1	5		3	6	
**Distribute educational materials**					26	1		21	1	1	3	
**Make training dynamic**				1	20			2			5	1
**Provide ongoing consultation**			1		2	1		2				

Values represent number of studies

Target Groups	Champions	Cleaning Staff	Doctors/Neonatologists	Facilitators	Healthcare Professionals, unspecified	IPC Team	Lactation Specialist/Consultant/Counselors	Mothers/Parents/Families/Caregivers/Visitors	Network/Collaborative	NICUs/Units	Nurses	Project Team/Task Force/ Work Group
**Update educational materials**					3			2				
**Use train-the-trainer strategies**					2		2	1			2	1

Target Groups (conz'd)	QI Team	Senior HCPs	Unit Leadership	n/a
**Conduct educational meetings or provide trainings**	2	1		11
**Conduct educational outreach visits**	1		1	

Values represent number of studies

Target Groups (conz'd)	QI Team	Senior HCPs	Unit Leadership	n/a
**Conduct ongoing training**	1			4
**Create a learning collaborative**				1
**Develop educational materials**				27
**Distribute educational materials**				22
**Make training dynamic**	1			3
**Provide ongoing consultation**	1			
**Update educational materials**				1
**Use train-the-trainer strategies**	2			2

Values represent number of studies

Use evaluative and iterative strategies

Actors	Auditors	Authors/Study Team	Champions	Cleaning Staff	Coordinating Center	Doctors/ Neonatologists	Epidemiology Team	Facilitator	Healthcare Professionals, unspecified	Hospital Administration	IPC Doctors/Nurses/ Personnel	IPC Team	Leadership, unspecified
**Audit and provide feedback**	2	1	1		1		1					1	1
**Conduct cyclical small test of change**													
**Conduct local needs assessment**		1		1							3	1	
**Develop a formal implementation blueprint**						1						1	
**Develop and implement tools for quality monitoring**								1	1				
**Develop and organize quality monitoring systems**	1	1	1			4			3		5	6	
**Obtain and use patients/consumers and family feedback**									1			1	

Values represent number of studies

Actors	Auditors	Authors/Study Team	Champions	Cleaning Staff	Coordinating Center	Doctors/ Neonatologists	Epidemiology Team	Facilitator	Healthcare Professionals, unspecified	Hospital Administration	IPC Doctors/Nurses/ Personnel	IPC Team	Leadership, unspecified
**Purposefully reexamine the implementation**		1	1	1					1			5	1
**Stage implementation scale up**												1	
**Update tools/systems for quality monitoring**	2	1	1							1		1	1

Actors (cont'd)	Network/ Collaborative	NICUs/Units	Nurse Educator	Nurse	Nurse Leaders	Project Team/Task Force/ Work Group	QI Leadership	QI Nurse	QI Team	Risk Management Team	Senior HCPs	Surveillance Team	Trust Board
**Audit and provide feedback**	2			2		1	1		3			1	

Values represent number of studies

Actors (cont'd)	Network/ Collaborative	NICUs/Units	Nurse Educator	Nurse	Nurse Leaders	Project Team/Task Force/ Work Group	QI Leadership	QI Nurse	QI Team	Risk Management Team	Senior HCPs	Surveillance Team	Trust Board
**Conduct cyclical small test of change**		2				1			9				
**Conduct local needs assessment**		1		2		5			12	1			
**Develop a formal implementation blueprint**						2			2				
**Develop and implement tools for quality monitoring**						1			2				
**Develop and organize quality monitoring systems**	1	4	1	3	1	2			1		1		
**Obtain and use patients/consumers and family feedback**						2			2				
**Purposefully reexamine the implementation**		1	1			5	3	1	9				1
**Stage implementation scale up**									1				

Values represent number of studies

Actors (cont'd)	Network/ Collaborative	NICUs/Units	Nurse Educator	Nurse	Nurse Leaders	Project Team/Task Force/ Work Group	QI Leadership	QI Nurse	QI Team	Risk Management Team	Senior HCPs	Surveillance Team	Trust Board
**Update tools/systems for quality monitoring**	2	1		2		1	1		3		1		

Actors (cont'd)	Unit Leadership	n/a
**Audit and provide feedback**	1	44
**Conduct cyclical small test of change**		15
**Conduct local needs assessment**		22

Values represent number of studies

Actors (cont'd)	Unit Leadership	n/a
**Develop a formal implementation blueprint**	2	4
**Develop and implement tools for quality monitoring**		2
**Develop and organize quality monitoring systems**	1	30
**Obtain and use patients/consumers and family feedback**		19
**Purposefully reexamine the implementation**		14
**Stage implementation scale up**		15
**Update tools/systems for quality monitoring**		10

Values represent number of studies

Target Groups	Cleaning Staff	Epidemiology Team	Hospital Administration	Healthcare Professionals, unspecified	IPC Doctors/ Nurses/Personnel	IPC Team	Mothers/Parents/Families/Caregivers/Visitors	Lactation Specialist/ Consultants/ Counselors	Leadership (various)	Network/Collaborative	NICUs/Units	Nurses	Patients/Neonates	Project Team/ Task Force/Work Group
**Audit and provide feedback**		1	2	35			2		2		9	6		
**Conduct cyclical small test of change**												1		
**Conduct local needs assessment**	1			2			1				4	4		
**Develop a formal implementation blueprint**				1			1				3			
**Develop and implement tools for quality monitoring**	1			3		1						1		
**Develop and organize quality monitoring systems**	1		1	14	1	1	2			1	4	5	2	
**Obtain and use patients/consumers and family feedback**				13		1	4	1				7		
**Purposefully reexamine the implementation**			2	10		1	3				2	3		1

Values represent number of studies

Target Groups	Cleaning Staff	Epidemiology Team	Hospital Administration	Healthcare Professionals, unspecified	IPC Doctors/ Nurses/Personnel	IPC Team	Mothers/Parents/Families/Car egivers/Visitors	Lactation Specialist/ Consultants/ Counselors	Leadership (various)	Network/Collaborative	NICUs/Units	Nurses	Patients/Neonates	Project Team/ Task Force/Work Group
**Stage implementation scale up**				1			5				1	1		
**Update tools/systems for quality monitoring**				3			1					1		

Target Groups (cont'd)	Pharmacists	Public Health Institution	Public/Commmunity	Unit Leadership	n/a
**Audit and provide feedback**		1	1	2	
**Conduct cyclical small test of change**					

Values represent number of studies

Target Groups (cont'd)	Pharmacists	Public Health Institution	Public/Commmunity	Unit Leadership	n/a
**Conduct local needs assessment**					
**Develop a formal implementation blueprint**					
**Develop and implement tools for quality monitoring**				1	
**Develop and organize quality monitoring systems**	1				
**Obtain and use patients/consumers and family feedback**				1	
**Purposefully reexamine the implementation**					
**Stage implementation scale up**					
**Update tools/systems for quality monitoring**					

Values represent number of studies

Utilize financial/incentivizing strategies

Actors	Hospital Administration	IPC Team	Trust Board	Unit Leadership	Project Team/Task Force/ Work Group	n/a
**Access new funding**			1			1
**Alter incentive/ allowance structures**	1	1		1		19
**Place innovation on fee for service lists/ formularies**					1	

Values represent number of studies

Target Groups	Champions	Doctors/Neonatologists	Facilitators	Finance Department	Healthcare Professionals, unspecified	Midwives	Mothers/Parents/Families/ Caregivers/Visitors	NICUs/Units	Nurses	QI Leadership	QI Team	Senior HCPs	n/a
**Access new funding**								1					
**Alter incentive/ allowance structures**	1	1	1		8	1	1	1	4			1	3
**Place innovation on fee for service lists/ formularies**				1									

Values represent number of studies
