# Supplementary material for: Investigating the implementation of infection prevention and control practices in neonatal care across country income levels: a systematic review
Source: Antimicrob Resist Infect Control. 2025 Feb 7;14:8. doi: 10.1186/s13756-025-01516-7 (PMC11806577; doi:10.1186/s13756-025-01516-7)
Supplement: Supplementary file 3 — Additional File 3: Coding Manual for Implementation Determinants based on updated Consolidated Framework for Implementation Research (CFIR). [file 13756_2025_1516_MOESM3_ESM.pdf]

Investigating the Implementation of Infection Prevention and Control Practices in Neonatal Care Across Country Income Levels: A Systematic Review

Emanuela Nyantakyi, Julia Baenziger, Laura Caci, Kathrin Blum, Aline Wolfensberger, Angela Dramowski, Bianca Albers, Marta Castro, Marie-Therese Schultes, Lauren Clack

SUPPLEMENTARY FILE

Coding Manual for implementation determinants based on updated Consolidated Framework for Implementation Research (CFIR)

Content

Innovation 3
Outer Setting 5
Inner Setting 9
Individuals 21
Characteristics of Individuals 22
References 27

Innovation

Innovation: Bundled or Single-component IPC practices or programs, employed in inpatient neonatal care settings and targeted toward neonates, healthcare workers (HCWs) or caregivers (i.e., parents, caregivers)

Construct	Definition	Subcodes	Example
**Innovation Source**	The degree to which the group that developed and/or visibly sponsored use of the IPC practice or program is reputable, credible, and/or trustable	Official Buy-In	*One of the respondents commented that probiotics remain in "regulatory purgatory" by citing the 2013 Acta Paediatrica article by Janvier and colleagues. The respondent suggested that the lack of FDA pro-duct regulation was a hindrance to completing clinical trials and moving forward probiotic use in VLBW and ELBW preterm infants in the United States. [1]*
		Credibility	*Joint statements from RCOG [Royal College of Obstetricians and Gynaecologists], RCM [Royal College of Midwives], PHE so there's no variation. It's not, you know, not ambiguous and guidelines, you know, get updated with time to implement. And you know con-sistent advice really. I think the other thing is joined up between, so like our microbiologists, we had to say this is the RCM, RCOG guideline. So, I think joint colleges, you know, joining together so if you say right, we have to test all inpatients, then think about all the different kinds of inpatients, children, neonates, maternity. [2]*
**Innovation Evidence-Base**	The degree to which the IPC practice or program has robust evidence supporting its effectiveness	Existing Evidence	*Since there was no evidence-based neonatal oral care procedure found in the literature review, a detailed procedure that focused on areas identified in the literature as bacterial reservoirs was designed using applicable adult best practices and oral swab kit manufacturer recommendations (Table 2). [3]*
		Patient Safety	*When asked about the best antiseptic to use in the NICU, many expressed their concerns about the limited safety data in premature infants, lack of good quality evidence, and the unapproved use of the current antiseptics in neonatal population. [4]*
**Innovation Relative Advantage**	The degree to which the IPC practice or program is better than other available IPC practices or programs or current practice	Superiority	*Glutaraldehyde-based solution, in comparison to hypochlorite, had the advantage of action even in the presence of organic matter, killing Mycobacteria and spores of Bacillus. The cost of hypochlorite-based solutions was relatively less. [5]*

Construct	Definition	Subcodes	Example
**Innovation Adaptability**	The degree to which the IPC practice or modified, tailored, or refined to fit local context or needs	Adaptability	*Concerns were raised by many that the guidance was not specific to maternity and it lacked detail on ways to address some of the unique aspects of care at birth. This created ambiguity, often related to PPE requirements in different scenarios. One participant described instances where staff felt unprotected when attending women in labour. [2]*
**Innovation Trialability**	The degree to which the IPC practice or program can be tested or piloted on a small scale and undone	-	
**Innovation Complexity**	The degree to which the IPC practice or program is complicated, which may be reflected by its scope and/or the nature and number of connections and steps	Ease of Use	*Ease of preparing milk with Prolact+ H2MF was rated as either very easy or easy by 96% of the respondents and the remaining 4% rated as neither easy nor difficult. Zero percent rated the process as difficult or very difficult. Ease of mixing milk with CMB-HMF was rated as very easy or easy by 83% of respondents, with 13% rating as neither easy nor difficult, and 4% rating the process as difficult or very difficult. [6]*
**Innovation Design**	The degree to which the IPC practice or program is well designed and packaged, including how it is assembled, bundled, and presented	Practice Variation	*Variation in feeding practice focused particularly on the timing of commencement of feeds, volume and frequency of trophic feeds, the interval between feeds, the rate of increasing feed volume in the at-risk population and the type of milk used for first feed [7]*
**Innovation Cost**	The degree to which the IPC practice or program purchase and operating costs are cost-efficient/effective	Costs	*A chlorhexidine mix with cetrimide was used to disinfect non-metallic surfaces, instead of chlorine solution, which would be less costly and more effective. [8]*

Outer Setting

Outer setting: The setting in which the inner setting exists, i.e., healthcare system and policy structures, sociocultural context

Construct	Definition	Subcodes	Example
**Critical incidents**	The degree to which large-scale and/or unanticipated events disrupt implementation and/or delivery of the IPC practice or program	COVID-19 Pandemic	*COVID pandemic has changed the functioning of healthcare aspects in many different ways. There were changes in the visitation policy in our unit where instead of parental visit being 24/7, restriction was implemented to the visit of father as 2 h. This could potentially affect the duration of KMC in this pandemic. [9]*
		Economic Crisis	*Respondents noted that these barriers were either caused or exacerbated by the economic crisis, particularly the memorandum that does not allow new recruitment in the public sector [10]*
**Local Attitudes**	The degree to which sociocultural values (e.g., shared responsibility in helping recipients) and beliefs (e.g., convictions about the worthiness of recipients) encourage the Outer Setting to support implementation and/or delivery of the IPC practice or program	Cultural Practices	*There are restrictions on visits by family and friends in both NICUs. Staff explained that this was an IPC measure. However, the reason for this restriction was not understood by most mothers. These mothers were opposed to these restrictions, as the local cul-ture encourages family members to celebrate the arrival of a new baby by seeing the newborn. [11]*
		Cultural Mindsets & Beliefs	*There was a belief that mothers could have another baby to 'replace' an infant who died, and therefore intensive neonatal care was not thought to be a priority [12]*
		Prejudice & Stigma	*Several providers mentioned that stigma and guilt about having a preterm infant are common in the local communities [13]*
**Local Conditions**	The degree to which economic, environmental, political, and/or technological conditions enable the Outer Setting to support implementation and/or delivery of the IPC practice or program	Care Accessibility	*delay in reaching appropriate care because of large distances to health facilities [14]*
		Data Reporting	*There were ten out of twelve country teams who reported failure to collect data on management of neo-natal infections, including data on antibiotic use, due to the lack of indicators for treatment of newborn infections in health information systems. Most country teams also highlighted that clinical records for sick and small newborns were inadequate at all levels of care [14]*
		Disease Prevalence	*In developing countries, the problem of HAIs is unacceptably high (13) with Nigeria having the highest rate of neonatal mortality in Africa and the world. [15]*

Construct	Definition	Subcodes	Example
		Drug Supply & Procurement	*A common challenge reported was inefficiency of procurement and supply management systems (9 out of 12 country teams), leading to erratic antibiotic supply. There are manufacturing gaps, and limited distribution of supplies, as well as inadequate systems for forecasting and restocking. Consequently there are frequent stock-outs and pre-scription of alternative, second line antibiotics. [14]*
		Local Economy	*The staff at the NNU is affected by the struggling Zimbabwean economy. This has led to strikes by different unions at the beginning of the year [16]_Supplement*
		Quality of Care	*At national level, respondents repeatedly identified that infection prevention is not a priority and is undervalued, and that the con-cept of infection prevention as a profession does not exist. As a result, the structures for infection control in hospitals and nationally are underdeveloped [10]*
		Quality of Healthcare System	*Quality of care was an important barrier for optimal management of neonatal infections at all levels of the health system [14]*
		Quality of Workforce	*Retaining professional, well-trained health workers in rural areas is challenging. [14]*<br><br>*At national level, respondents repeatedly identified that infection prevention is not a priority and is undervalued, and that the con-cept of infection prevention as a profession does not exist. As a result, the structures for infection control in hospitals and nationally are underdeveloped [10]*
		Workforce Level	*indicated a shortage of health care workers with adequate knowledge and skills to competently identify and manage newborn infections, especially in primary care settings [14]*
**Partnerships & Connections**	The degree to which the Inner Setting is networked with external entities, including referral networks, academic affiliations, and professional organization networks	Initiatives	*Institution-based initiatives such as Baby Friendly Hospital designation were mentioned by some participants as facilitators to promoting breastfeeding and the use of human milk. [17]*
		Institutional Collaborations	*Wie von der KRINKO empfohlen, stehen alle Zentren im engen Kontakt mit den mikrobiologischen Laboren und erhalten umgehend bei Nachweis von multiresistenten Isolaten eine Information [18]*

Construct	Definition	Subcodes	Example
		Networks	*The group also is part of the New Jersey NICU collaborative, the focus of which is to foster QI in the care of NICU patients, including decreasing CLABSI rates in neonates. The study NICU also belongs to the national catheter-associated bloodstream infection (NCABSI collaborative, which focuses on prevention of CLABSIs [19]*
**Policies & Laws**	The degree to which Legislation, regulations, professional group guidelines and recommendations, or accreditation standards support implementation and/or delivery of the IPC practice or program	Policies & Regulations	*[National] incentives to motivate staff, support job satisfaction, and career development were lacking. [14]*<br><br>*Respondents noted that these barriers were either caused or exacerbated by the economic crisis, particularly the memorandum that does not allow new recruitment in the public sector [10]*
**Financing**	The degree to which funding from external entities (e.g., grants, reimbursement) is available to implement and/or deliver the IPC practice or program	Healthcare Financing	*Seven out of 10 country workshop participants reported insufficient funding for procurement, and continuous, sustainable distribution of antibiotics. High out-of-pocket expenses, arising from consultancy fees, transport, and treatment, as well as some unofficial payments, were a burden for families, especially for second- and third-line antibiotics. Limited coverage of financing schemes and insurance mechanisms for care of sick newborns was highlighted by all country teams. [14]*<br><br>*(..)and delay in receiving appropriate care and antibiotic treatment due to financial barriers (e.g. consultation fees in teaching hospitals or costs of second line antibiotics) [14]*
**External Pressure**<br>*If none of the subcodes apply, please code under "external pressure"*	The degree to which external pressures drive implementation and/or delivery of the IPC practice or program:		
	A. *Societal Pressure*: The degree to which mass media campaigns, advocacy groups, or social movements or protests drive implementation and/or delivery of the IPC practice or program	-	
	B. *Market Pressure*: The degree to which competing with and/or imitating peer entities drives implementation and/or delivery of the IPC practice or program	-	
	C. *Performance-Measurement Pressure*: The degree to which quality or	Benchmarking	*In 2011, our NICU CLABSI rate was almost triple the nationwide Children's hospital benchmark aver-age. Therefore, a concentrated*

Construct	Definition	Subcodes	Example
	benchmarking metrics or established service goals drive implementation and/or delivery of the IPC practice or program		*effort to design a bundled approach focusing on hand hygiene, central line insertion checklists, scrub the hub, sterile cap/tubing change frequency, and prompt central line removal was initiated. [20]*

Inner Setting

Inner setting: Inpatient neonatal care settings (e.g., Kangaroo care (KC) hospitals/units, acute neonatal hospitals/units, neonatal intensive care hospitals/units, labor hospitals/units, postnatal hospitals/units, pediatric hospitals/units with admission of neonates)

Construct	Definition	Subcodes	Examples
**Structural characteristics** *If none of the subcodes apply, please code under "Structural Characteristics"*	The degree to which infrastructure components support functional performance of the inpatient neonatal care setting<br>A. *Physical Infrastructure*: The degree to which layout and configuration of space and other tangible material features support functional performance of the Inner Setting regarding the implementation IPC practices/programs	Area Labeling	*no signage for the isolation area.' [21]*
		Equipment Accessibility	*The soaps were not properly placed leading to contamination of the soap bars. There was availability of only two hand driers in the whole unit. Cloth towels used for hand drying were changed infrequently [22]*
		Equipment Location	*The variability in product type, location, and means of transport [23]*
		Quality of Space	*(...) absence of basins at entrances and in the tearoom to promote hand hygiene, walls and floors of the neonatal ICU not intact (...) [21]*
		Sight	*limited sight lines in the new NICU to observe hand hygiene and practice 200% accountability*<br><br>*decreased sight lines in the new environment, making observation of infection prevention practices, such as hand hygiene and equipment disinfection, more difficult [23]*
		Size of Space	*Larger NICUs had significantly higher overall compliance with the international CLABSI prevention guidelines [24]*
		Space Layout	*increased environmental touch-points given a larger geographic footprint, including wall-mounted touch pads to call for assistance and a heavy reliance on mobile communication devices [23]*
		Temperature	*Cold outside (2); Cold inside (5) [25]*
		Volume	*hat performing hand hygiene was complicated by managing equipment alarms and supplies outside the neonate environment. Alarms go off frequently and in the event that staff are required to leave the neonate environment to silence an alarm, ABHR is not available at the point of interaction (i.e. the bed) and must be accessed at the work counter, which is not within arm's reach. [26]*
		Other	*Clutter [27]*
	B. *Information Technology Infrastructure:* The degree to which technological	Availability of Tech Support	*IFs were comfortable with the technology, valued the telemedicine team's tech support [28]*

Construct	Definition	Subcodes	Examples
	systems for tele-communication, electronic documentation, and data storage, management, reporting, and analysis support functional performance of the Inner Setting regarding the implementation IPC practices/programs	Data Management	*Nur wenige Zentren verfügen über eine Ausstattung mit elektronischen Systemen die Langzeitdaten zu Erregern und Resistenzen erfassen bzw. analysieren können. [18]*
		Information System	*Participants shared details of the IT set-up on their units being incompatible with what the guidance was suggesting. For example, this participant spoke of a lack of resources to enable individuals to work from home.*<br><br>*Additionally, this participant spoke of the inability to alter handovers due to the lack of available software in some areas of the unit. [2]*
		Availability of Internet	*A feedback using the SurveyMonkey application was obtained regarding the feasibility and usefulness of the game. Only 5 out of 25 (20%) nurses were able to complete the game. The major reasons for non-completion were poor internet connectivity [29]*
		Use of Mobile Phones	*Mobile phones are ever-present. Mothers, nurses and doctors often take them out of pockets and bags, they are plugged into the sockets everywhere around the wards. Mobile phones, though known hearths of bacteria, are necessary tools in the assessment of patients, calculation of numbers and communication between staff. It seems, however, that there is little awareness of their impact on IPC.*<br><br>*Mobile phones are ever-present and are constantly used by staff and patients' relatives [16]*
	C. *Work Infrastructure*: The degree to which the organization of tasks and responsibilities within and between individuals and teams, and general staffing levels, support functional performance of the Inner Setting regarding the implementation IPC practices/programs	Admission/Discharge Policies	*A less restrictive admission policy set us apart from most NICUs, by allowing the visitation of siblings in any age group. [30]*
		Auditing Practices	*Hospital-acquired infection prevention was not well implemented and audits of maternal-perinatal care were rare, and did not include the assessment for newborn infections based on risk factors. [14]*
		Availability of Specialized Staff/Teams	*Successes in implementing PHE guidance was often related to staff being dedicated to COVID-specific roles, which allowed for the cascade of appropriate and timely information to other maternity staff members [2]*
		Clarity of Responsibilities	*The shortage of orderlies was further aggravated by the fact that most of the orderlies interviewed also performed healthcare related tasks such as antenatal care, wound dressing, prescribing medications and assisting deliveries, which significantly reduced the time they spent on cleaning activities. [31]*

Construct	Definition	Subcodes	Examples
		Contracting	*All new nurses were hired on temporary contracts. All interviewees universally agreed that "More health care providers are needed." [32]*
		Documentation Practices	*there were no records of who was responsible for the regular filling of bottles with hand spray and the bottles were dirty.' (#5773 - Theron 2022, S. 4)*
		Hectic	*This code refers to activities that nurses must do in a hurry or the perception that the activities must be done quickly due to high census or number of tasks. "And I, you know, people, they get in a hurry and if they are not worried about, you know, if they think scrubbing it with an alcohol wipe a couple of times is good enough for them then that's all they are going to do." [33]*<br><br>*The hectic environment of the NICU can lead to gaps in reviewing the need for the catheter during medical rounds daily. [34]*
		Hospital Type	*The results showed that the influencing factors of knowledge were as follows: Grade III class B hospital (compared with Grade II class A hospital) (B = 0.02, p = 0.01), intermediate title (B = 0.02, p = 0.02), and senior title (B = 0.04, p = 0.01) (compared with primary title). No factors significantly affected attitude. [35]*
		Legal issues	*(3) legal and institutional barriers to timely administration. [36]*
		Length of Stay	*prolonged hospital length of stay [37]*
		Process Delays	*Feeds often delayed for 2-3 d waiting for MOM [6]*
		Shift System	*Furthermore, there were many mentions of staff being unable to attend fit testing clinics due to shift patterns which, in some cases, meant that participants had still not been fit tested [2]*
		Staffing Levels	*Organizational barriers hampered recruitment as organizations did not pay IFs, and the research supported only modest incentives. QI teams did not get release time from clinical care, and bed-side nursing representation was less than hoped [28]*<br><br>*In 12% of the facilities without an operating theatre (n ¼ 24), there was no SBA during the morning and night shift prior to the survey (Table 2); whereas, all facilities with an operating theatre had at least one SBA present. Staffing shortages and high caseloads were frequently mentioned during qualitative interviews as reasons for poor IPC. [31]*

Construct	Definition	Subcodes	Examples
		Staff Rotation and Deployment	*an important barrier given the high rotation of staff [16]*<br><br>*Staff deployment*<br>*Staff allocation rosters were rationalized according to risk exposure and client load. [38]*
		Standardization	*Barriers to the routine implementation of PHE guidance existed due to units adopting different processes for the testing of both women and partners. Specifically, some participants spoke about arranging testing for women prior to attending the unit for an elective caesarean section, whilst others did not test any elective admissions. [2]*
		Job Differentiation	*Another challenge noted by one IF was that the med-ical team was not allowed to educate the nursing staff [39]*<br><br>*There is only one dedicated housekeeper for weekdays and one for evenings, and the latter's job role is limited to emptying waste, restocking ABHR and cleaning infant bays for new admissions when required [26]*
		Time Availability	*They recognized that there were certain basic nursing practices that they should be providing (like bathing) but felt they did not have the time to perform them, especially when there were life-saving procedures to be completed [12]*
		Turnover/Attrition	*With substantial staff and resident turnover [36]*
		Visitor Traffic	*high visitor traffic without control access (not only mothers who attend six to eight times per day for feeding, but for example full surgical teams with medical students for training) potentially promoted infection transmission [16]*
		Work Processes	*Prior to interventions, the person consenting for the infant's vaccination was limited to the biological mother. However, frequently she was not easily available which delayed consent. When she did consent, it was unclear if verbal consent was sufficient and whether a system for consistent documentation existed. [36]*
		Workload	*NICUs with a high number of admissions (and high turnover) had lower overall compliance [24]*

Construct	Definition	Subcodes	Examples
**Relational Connections**	The degree to which there are high quality formal and informal relationships, networks, and teams within and across Inner Setting boundaries (e.g., structural, professional)	Cross-Collaboration	*We cannot overemphasize the value of hospital-wide support and multilevel collaboration. Our hospital administration supported nursing champions, who had dedicated protected time to work on CLABSI reduction initiatives. [40]*
		Family Support	*Almost half of providers discussed the importance of family support, with two specifically commenting on the role of the mother-in-law in influencing KMC practice in facilities [13]*
		Involvement of Patients/Families	*IPC requires teamwork and is a responsibility of all staff, patients and their relatives [16]*
		Peer Support	*Less than one-fourth (21.1%) of the postnatal mothers were getting help from other postnatal mothers, and nearly one-third (30.2%) of the participants acquired help from other family members [41]*
		Representation	*Another participant described that obstetric representation at hospital-wide meetings allowed for regular updates of the ever-changing situation. [2]*
		Teamwork & Collaboration among Staff	*This NICU has a multidisciplinary QI committee that meets once a month, and this team provided the foundation of this QI work. The clinical staff has an established culture of evidence-based collaborative decision making [19]*
**Communications**	The degree to which there are high quality formal and informal information sharing practices within and across Inner Setting boundaries (e.g., structural, professional)	Communication Flow	*This informal communication on the checklist allowed for rapid follow-up by the clinical nurse specialist or NICU medical director [42]*
		Data Tracking	*The interview results also suggested that overall monitoring of IPC was generally poor across the facilities, reflecting the lack of formal committees charged with this role as captured by the Facility Needs Assessment Tool. [43]*<br><br>*An initial barrier for the team was lack of data to support rapid tests of change [44]*
		Meetings & Discussions	*From a practical perspective, huddle coordination has been a key factor in facilitating timely huddle completion. Huddle times are prescheduled in the QI team calendars by the infection control practitioner and if there are no CLABSIs, the huddle is cancelled [34]*
		Patient-Provider Interaction	*During our study, we learnt that one of the key barriers to adequate KMC practice was the communication gap between the healthcare worker and the parents which existed secondary to lack of complete knowledge and awareness about the ideal practice [9]*

Construct	Definition	Subcodes	Examples
		Peer-to-peer Feedback	*Social influence was a very important factor for IPC in the unit. Staff's practices around IPC were influenced by their colleagues, e.g., through reminders and encouragement, as well as explicit instructions. [16]*
		Provider Feedback	*Second, incorporating a data-driven feedback system promoted buy-in and team engagement. [45]*
		Quality of Communication	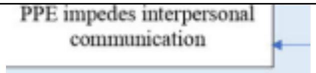
**Culture**<br>*If none of the subcodes apply, please code under "Culture"*	The degree to which there are shared values, beliefs, and norms across the Inner Setting	-	-
	A. *Human Equality-Centeredness*: The degree to which there are shared values, beliefs, and norms about the inherent equal worth and value of all human beings	Hierarchies	*Several reasons for not actively participating in the WhatsApp group were presented. The hierarchical structure of staffing contributed to fear and hesitation around being judged by peers and superiors for one's remarks, comments, or suggestions:*
	B. *Recipient-Centeredness*: The degree to which there are shared values, beliefs, and norms around caring, supporting, and addressing the needs and welfare of recipients	-	
	C. *Deliverer-Centeredness*: The degree to which there are shared values, beliefs, and norms around caring, supporting, and addressing the needs and welfare of deliverers	-	-
	D. *Learning-Centeredness*: The degree to which there are shared values, beliefs, and norms around psychological safety, continual improvement, and using data to inform practice	Learning & Improvement Culture	*The initial implementation period of the new central line "bundles" was characterized by poor compliance rate by the nursing team, thought to be primarily due to most of the senior nursing staff being resistant to change. [46]*

The following constructs in this domain are specific to the implementation or delivery of the innovation (i.e., IPC practice/program)

Construct	Definition	Example	
**Tension for Change**	The degree to which the current situation is intolerable and needs to change	Infection Rates	*for proper HH among parents and family members. The IPC recognized this rate of HH compliance was not acceptable [30]*
		Outbreaks	*Outbreaks in the unit can temporarily improve IPC resources and practices (e.g. of cholera) [16]*

Construct	Definition	Example	
**Compatibility**	The degree to which the IPC practice or program fits with workflows, systems, and processes	Adaptation of Work Processes	*Adaptation to new workflows "Abandoning family centered rounds and minimizing residents' involvement in direct patient care."*<br>*"Newborns don't leave parent rooms."*<br>*"[W]e lost our cuddlers to help with management of our neonatal abstinence infants."*<br>*"Dealing with early discharge pressures."*<br>*"Ensuring adequate follow up with rapid discharges."* [47]
		Emergencies	*They recognized that there were certain basic nursing practices that they should be providing (like bathing) but felt they did not have the time to perform them, especially when there were life-saving procedures to be completed* [12]
		Length of Tasks	*nurses reported difficulty in managing colostrum collection in addition to other nursing routines since it was quite time consuming* [48]
		Order of tasks	*First, obtaining consent from mothers to bathe the babies with CHG solution was routinely mentioned by doctors and nurses as a barrier to timely completion of the task* [12]
		Task Fit	*The set-up and flow of some units made it difficult to implement and comply with published guidance, whilst many participants detailed issues with being unable to comply with published guidance due to the nature of their roles and a lack of availability and accessibility to PPE.*<br><br>*Many participants spoke of the nature of their roles not allowing for compliance with the guidance. In particular, there were several mentions of the inability to comply with social distancing guidelines whilst delivering clinical care. This participant details how the layout and care plan on their neonatal unit does not fit with the distancing guidance for staff* [2]
		Timing	*Four participants referred to the undesirable timings related to the release of guidance updates. Updates were released on Friday afternoons, and experiences frantically trying to implement the new guidance before the weekend were shared.*

Construct	Definition	Example	
**Relative Priority**	The degree to which implementing and delivering the IPC practice or program is important compared to other initiatives	Institutional Priority	*nursing managers' inattention towards the scientific implementation of nursing care [49]*<br><br>*It was suggested that this attitude led to a lack of public awareness about the NICU and subsequent devaluation by hospital administrators. Respondents believed that resource allocation reflected these priorities [12]*
		Management/Leadership Involvement	*Departmental leadership played a very critical role as an enabler ("leadership in crisis"). Active engagement in surge preparedness for IPC and pandemic combat ("leading from front") was deemed as a major leadership trait by the participants. [38]*
**Incentive systems**<br>*Please take note of the congruent ERIC strategies Alter incentive/allowance structures and Develop disincentives*	The degree to which tangible and/or intangible incentives and rewards and/or disincentives and punishments support implementation and delivery of the IPC practice or program	Disincentives	*administrative sanctions, and rewards [50]*
		Incentives	*The need for 'incentives' e.g. bonuses to act as a motivating factor for cleaning staff was noted [51]*
		Performance Evaluation	*No IPC emphasis in staff appraisals [11]*
		Remuneration	*One nurse mentioned, "The low salary and the weak potentials for financial increments increased the turnover rate of the already appointed nurses. [32]*
**Mission alignment**	The degree to which implementing and delivering the IPC practice or program is in line with the overarching commitment, purpose, or goals in the Inner Setting	Institutional Emphasis	*Heavy emphasis on clinical care delivery [11]*
		Shared Goals/Visions	*A key factor in the motivation of our NICU team has been the alignment of our goals with hospital-wide initiatives aimed to achieve a reduction in health care–associated condi-tions (HACs), among which CLABSI was included [34]*
**Available Resources**<br>*If none of the subcodes apply, please code under "Available Resources"*	The degree to which resources available to implement and deliver the IPC practice or program.	General Resources	*However, not all staff believed that training would be sufficient: "the challenges come on implementation because the resources were never availed for training we just train people but resources are not there" [16]*
	A. *Funding*: The degree to which funding is available to implement and deliver the IPC practice or program	Financial Resource Availability	*barriers included nursing staff turnover, buy-in from staff, funding [37]*
	B. *Space*: The degree to which physical space is available to implement and deliver the IPC practice or program.	Overcrowding	*Overcrowding [29]*
		Privacy	*getting KMC private space in the hospital [41]*
		Space Availability	*Space constraints (assistive staff, equipment) [30]*

Construct	Definition	Example	
	C. *Materials & Equipment*: The degree to which Supplies are available to implement and deliver the IPC practice or program	Availability of Materials & Equipment	*Shortages of PPE, especially at the start of the pandemic, was a big issue for a lot of units. Participants described instances where PPE was being reused, insufficient PPE was being worn in certain scenarios and resources were used for alternative means. Often, this led to participants sourcing PPE elsewhere [2]*
		Supply Chain of Materials & Equipment	*Alcohol gel was not included on MINSA's basic supplies list and was therefore not available at many facilities [8](#3539 - López 2013)*
	D. *Characteristics of Materials & Equipment*[1]: The degree to which the available materials/equipment support the delivery of the IPC practice or program	Comfortability	*cumbersome administrative processes, uncomfortable beds (no backrest to support breastfeeding mothers), and lack of privacy and comfort. [38]*
		Equipment Labeling	*Hand hygiene equipment is well labelled [25]*
		Legibility of Equipment	*Bedside maintenance checklists were revised to be more user friendly, be easier to read, and have more areas for nursing input. [40]*
		Quality of Materials & Equipment	*Additionally, the viscosity varied such that a uniform volume of sanitizer was not consistently dispensed. [23] (#549 - Barrett 2023)*<br><br>*'cleaning equipment was old and dirty as cleaners brought their own cleaning equipment (e.g. mops and buckets) from home [21]*
		Size of Equipment	*staff needing to be re-tested or wear masks that did not fit properly. [2]*
		Skin Irritation	*Frequent Gel Use Causes Skin Irritation [52]*
		Texture/Smell/ Temperature	*Some respondents did not like the texture or smell of the hand rub and believed it led to excessive hand dryness [12]*
		Other Characteristics of Materials & Equipment:	*The unit experimented with adding off-the-shelf ABHR holders to the beds but since they were not permanently fixed, they would go missing and were expensive to replace*

[1] added construct

Construct	Definition	Example	
			in the case of extremely premature infants, there are many materials that are not suit-able for our type of patient
**Access to knowledge & information**	The degree to which guidance and/or training is accessible to implement and deliver the IPC practice or program	Accessibility of Information	*In discussing breaches, the PE/HE diagram was reviewed by some staff, suggesting the distinctions and actions required between zones were not clear to them. Some HCWs had trouble finding the diagram among other posting [26]*
		Availability of Education Staff	*barriers (e.g., few nursing educators, no support to attend meetings or conferences or formal QI collaboratives [28]*
		Consistency of Information	*One major barrier to the implementation of PHE guidance was related to participants' beliefs that much of the guidance was contradictory, both in updates and between specialities and hospital sites, which often led to great confusion. [2]*<br><br>*Another barrier was the inconsistent advice and education provided to pregnant and postnatal women from staff providing care. [53]*
		Counseling	*Inadequate counseling on IPC for newborn care [38]*<br><br>*Interaction between the doctor, RDMs, and family members got compromised due to fear and limited visitations in face of staff shortage. The gap for counseling services was most glaring at places where these were most needed.*
		Education Material	*Satisfaction with training materials, resources and scenario-based training was also highlighted. This participant talks about the ease in understanding and implementing PPE guidance presented on posters around their unit*<br><br>*There is a lack of design affordances to clarify IPAC risks and education spance and tools to help clarify risks*
		Expert Guidance/Supervision	*The two most common areas of need identified were (1) ability to elicit external expert guidance [44]*
		(Fast) Evolving Information	*The official online HBV hospital guideline was outdated reflecting the old recommendations. [36]*
		Guideline Availability	*there were no available treatment guidelines,*

Construct	Definition	Example	
			lack of prescribing guidelines, and competing NEC risk-reduction strategies. [1]
		Patient/Families Information	*One of the barriers related to the limited antenatal breastfeeding information received by mothers. Women who deliver prior to 34 weeks' gestation miss the opportunity to receive the routine antenatal education delivered to mothers because of the timing of their delivery.*<br><br>*Limited antenatal breastfeeding information as antenatal classes missed due to early delivery. [53]*<br><br>*Posting the number of days from the last infection on a centralized poster board in the NICU allowed caregivers, as well as families, to become powerful and informed team members. [40]*
		Information Source	*Two-thirds (64.84%) of postnatal mothers got information from health professionals after being admitted to the hospital. Only a few 44 (7.10%) mothers got information from the mass media, others 55 (8.87%), 47(7.58%), 38 (6.1%), 22 (3.55%), and 12 (1.96%) got information from the internet, colleagues, relatives, neighbors, and others [41]*
		Language	*Finally, Anay shared that language may have presented a barrier to participation. Some of the group members were not as confident in English, which was the dominant language used in WhastsApp communication [54]*
		Poster & Signage	*This participant talks about the ease in understanding and implementing PPE guidance presented on posters around their unit*
		Promotion of Education	*Neonatology fellows were encouraged to participate in conferences, webinars, and data collection as part of their QI scholarly activities [40]*<br><br>*barriers (e.g., few nursing educators, no support to attend meetings or conferences or formal QI collaboratives [28]*

Construct	Definition	Example	
		Reminders	*There were very few hand hygiene reminders and posters It was assessed that the taps were hand operated and poor availability of soaps and alcohol hand rubs (AHRs). [22]*
		Scope of Training	*Of those facilities providing training, the interviews revealed that training was not comprehensive; reaching only a small number of cleaners and was generally limited to training in hand washing and surface cleaning. [43]*
		Availability of Training	*Training was a key area of need as raised repeatedly throughout the interviews with stakeholders*<br><br>*Training was notably absent in the majority of facilities in Gujarat and in the two facilities with the lowest SOH-V scores in Dhaka Division.*
		Training/Education Method	*Secondly, the teaching of central line placement skills for young residents was inadequate and inconsistent, being frequently based on the educational model "see one, do one, teach one" [55]*

Individuals

Individuals: The roles of individuals involved in the implementation of IPC practices/programs

Construct	Definition	Example
**High-level Leaders**	Individuals with a high level of authority, including key decision-makers, executive leaders, or directors	
**Mid-level Leaders**	Individuals with a moderate level of authority, including leaders supervised by a high-level leader and who supervise others	
**Opinion Leaders**	Individuals with informal influence on the attitudes and behaviors of others	
**Implementation Facilitators**	Individuals with subject matter expertise who support implementation	
**Implementation Leads**	Individuals who lead efforts to implement the IPC practice or program	
**Implementation Team Members**	Individuals who collaborate with and support the Implementation Leads to implement the IPC practice or program, ideally including Innovation Deliverers and Recipients	
**Other Implementation Support**	Individuals who support the Implementation Leads and/or Implementation Team Members to implement the IPC practice or program	
**Innovation Deliverers**	Individuals who are directly or indirectly delivering the IPC practice or program	
**Innovation Recipients**	Individuals who are directly or indirectly receiving the IPC practice or program	

Characteristics of Individuals

Individuals: The roles of individuals involved in the implementation of IPC practices/programs

If you are coding one of the following constructs, please specify the Individuals involved (see Individuals)

Construct	Definition		*Example*
**Need**	The degree to which the individual(s) has deficits related to survival, well-being, or personal fulfillment, which will be addressed by implementation and/or delivery of the IPC practice or program	Gender	*The influencing factors of self-reported practice were as follows: being female… Gender (compared with male) [35]*
		Need for Invasive Devices	*prolonged requirement for central venous lines [37]*
		Pain or Discomfort	*Postdelivery pain, medical reasons for mother being nonambulatory [48]*
		Patient Acuity	*including high patient acuity [37]*
**Capability**	The degree to which the individual(s) has interpersonal competence, knowledge, and skills to fulfill the role	Education Level	*Seven per cent of mothers had no formal education at all, while 41% of mothers had attended school up to the primary level (Table 1), but most were not able to understand or read English well. This presented a challenge with the use of written media as a channel of IPC communication, as HH posters and instructions were mostly in English. Mothers expressed the desire to receive IPC information via simplified messages on social media, using visual illustrations and/or in the local languages (Figure 1, Individual-level factors). [11]*
		Execution / Skill / Technique	*The root causes were the lack of sufficient training for service provider, the low number of nurses to number of patients, the lack of adequate facilities for washing hands, and the use of gloves instead of washing hands. [56]*
		Familiarity with Guidelines & Processes	*Lack of familiarity with CLABSI care instructions [49]*

Construct	Definition	*Example*
	Knowledge about Evidence	*Of the respondents surveyed, 8 (27.6%) were not familiar with any evidence surrounding probiotics. [1]*
	Knowledge/Familiarity with IPC Practice	*A 2015 survey of our NICU staff, conducted prior to the development of a formal unit-based QI program, indicated that only 34% of staff were aware of our unit's clinical outcomes, and most staff were not actively involved in (nor aware of) QI activities in our NICU. [44]*
	Memory & Attention	*forgetfulness and preference for the use of gloves were the most commonly cited barriers [57]*
	Professional Status/Role	*The univariate analysis showed that knowledge was significantly correlated with gender, hospital grade, job position, professional title, hospital's emphasis on establishing a nosocomial infection management department, hospital's emphasis on the prevention and control of MDRO infection, and the department's emphasis on the prevention and control of MDRO infection (p < 0.05 or 0.01) [35]*
	Professional Education	*Managers noted that currently training is 'suboptimal' and discussed the lack of knowledge and awareness of Class 4 (cleaning) staff [43]*
	Work Experience	*Results are summarized in Table 5 and showed that providers with more years of experience had lower opinion scores on non-sterile gowns (p < 0.01). [58]*
	Other	*During one team discussion, it was found that the attendants (parents and relatives) and ward aayas were not able to understand what the AHR was [59]*
**Opportunity**	Material Resources	*One mother stated that lack of family support and lack of money for mothers to buy food are*

Construct	Definition		*Example*
	The degree to which the individual(s) has availability, scope, and power to fulfill the role		*common in the newborn unit. A father discussed difficulties with KMC related to cost and increased time away from work. [13]*
		Physical Opportunity	*father not able to take part in giving KMC [9]*<br><br>*RDMs [Recently Delivered Mothers] lacked support as attendants entry into the wards was restricted [38]*
		Social Influence & Pressure	*This sentiment, expressed by a senior staff person, encapsulated one reason why group members were reluctant to actively participate. According to Dr Vijay, they did not want to be held accountable for any specific suggestion or idea [54]*
**Motivation**	The degree to which the individual(s) is fulfilling the role	Attitude & Awareness	*Apart from awareness of ANTT, the cost of using gloves and availability of closed ports were concerns [60]*<br><br>*Through this analysis, we identified a number of problems that could potentially contribute to non-compliance to AHR uses, including stakeholders' awareness regarding use of hand rub and its effect [59]*
		Beliefs about Consequences	*The head nurse revealed that the more people observe others doing it, the more they do it. However, some participants emphasized the hierarchical nature of the workplace and fear of repercussions, such as hindering career advancement, would prevent widespread adoption of this strategy [32](#5119 - Salem 2017, S. 3)*
		Buy-In	*(...) buy-in through the perspective of the physician IF was starkly different, finding that there was not only buy-in from peers but also from nursing staff, lactation consultants, and*

Construct	Definition		*Example*
			nutritionists. Buy-in for the content presented, despite its basis of evidence, was found to be a barrier by the IFs due to continued resistance for changing practice [39]
		Emotions & Personal Beliefs	*HCWs experience physical and cognitive exertion to adapt to and manage spatial limitations within the environment which may be compromising infection practice [26]*<br><br>*Some senior nurses felt that student nurses who used gloves while changing soiled cot sheets were wasting the limited supplies. Some HPs also used normal examination gloves and sterile gloves interchangeably, although there were guidelines for the use of each type of glove. [11]*
		Motivation	*"Post NeoECHO discussions together and really brainstorm together. I think having that engagement makes people feel empowered, and they want to continue." [39]*
		Intentions & Goals	*Many interviewees had intentions or goals that were congruent with adhering to IPC practices and improving patient outcomes, as well as protecting their own health or the health of their families. [16]*
		Optimism	*Despite the aforementioned challenges we found that all respondents believed it was possible to reduce HAIs in the NICU and that this was an important goal [12]*
		Professional Confidence	*Additionally, doctors and nurses did not have confidence in the test results reported by the laboratory as they had already formed an opinion on the case and so laboratory results were not considered reliable [8]*

Construct	Definition		*Example*
			Many respondents reported feeling uncomfortable in advising colleagues about their adherence to infection control guidelines. For example, a nurse said, "I felt uncomfortable approaching colleagues to suggest improvements they could make to their practice or raising issues with nursing or clinical staff if I observed any malpractice [32]
		Professional Role & Identity	*Nurses took the lead in training mothers on how to clean their baby's immediate environment, but remained willing to ensure that cleaning was thorough, even when mothers were absent, unable or unwilling to partake in cleaning efforts. [61]*<br><br>*including downloading documents and watching videos. Additionally, being accessible all the time resulted in little separation or balance between work and personal life. One respondent stated she was able to remain up to date on work through the WhatsApp communication while she was on leave. She viewed remaining informed as an asset, but it did not allow her to separate herself from work while away [54]*
		Reinforcement	*Almost all the nurses claimed that there had been no academic sessions for them regarding HH practices within at least previous six months, which could motivate them more in that regard. [62]*
		Satisfaction	*job satisfaction [63]*

References

1. Anderson S: **Barriers to Knowledge Translation Regarding the Use of Probiotics as a Risk-Reduction Strategy for Necrotizing Enterocolitis.** *Adv Neonatal Care* 2016, **16**(4):E3-e14.
2. Hanley SJ, Jones AB, Oberman J, Baxter E, Sharkey D, Gray J, Walker KF: **Implementation of Public Health England infection prevention and control guidance in maternity units in response to the COVID-19 pandemic.** *Journal of Hospital Infection* 2022, **129**:219-226.
3. Weber CD: **Applying Adult Ventilator-associated Pneumonia Bundle Evidence to the Ventilated Neonate.** *Adv Neonatal Care* 2016, **16**(3):178-190.
4. McCord H, Fieldhouse E, El-Naggar W: **Current Practices of Antiseptic Use in Canadian Neonatal Intensive Care Units.** *Am J Perinatol* 2019, **36**(2):141-147.
5. Somasekhara Aradhya A, Mercy L, Reddy V, Venkatagiri P: **Reducing the costs of floor cleaning in a level III NICU of Bangalore rural: a quality.** *BMJ Open Qual* 2022, **11**(Suppl 1).
6. Delaney Manthe E, Perks PH, Swanson JR: **Team-Based Implementation of an Exclusive Human Milk Diet.** *Adv Neonatal Care* 2019, **19**(6):460-467.
7. Radbone L, Birch J, Upton M: **The development and implementation of a care bundle aimed at reducing the incidence of NEC.** *Infant* 2013, **9**(1):14-19.
8. López S, Wong Y, Urbina L, Gómez I, Escobar F, Tinoco B, Parrales A: **Quality in practice: preventing and managing neonatal sepsis in Nicaragua.** *Int J Qual Health Care* 2013, **25**(5):599-605.
9. Jain H, Chandrasekaran I, Balakrishnan U, Amboiram P, D V: **Quality improvement initiative approach to increase the duration of Kangaroo Mother Care in a neonatal intensive care unit of a tertiary care institute in South India during the COVID-19 pandemic.** *Journal of Pediatric Nursing* 2023, **68**:74-78.
10. Triantafillou V, Kopsidas I, Kyriakousi A, Zaoutis TE, Szymczak JE: **Influence of national culture and context on healthcare workers' perceptions of infection prevention in Greek neonatal intensive care units.** *Journal of Hospital Infection* 2020, **104**(4):552-559.
11. Sunkwa-Mills G, Rawal L, Enweronu-Laryea C, Aberese-Ako M, Senah K, Tersbøl BP: **Perspectives and practices of healthcare providers and caregivers on healthcare-associated infections in the neonatal intensive care units of two hospitals in Ghana.** *Health Policy and Planning* 2020, **35**(Supplement_1):i38-i50.
12. Cowden C, Mwananyanda L, Hamer DH, Coffin SE, Kapasa ML, Machona S, Szymczak JE: **Healthcare worker perceptions of the implementation context surrounding an infection prevention intervention in a Zambian neonatal intensive care unit.** *BMC Pediatrics* 2020, **20**(1):432.
13. Morgan MC, Nambuya H, Waiswa P, Tann C, Elbourne D, Seeley J, Allen E, Lawn JE: **Kangaroo mother care for clinically unstable neonates weighing ≤2000 g: Is it feasible at a hospital in Uganda?** *J Glob Health* 2018, **8**(1):010701.
14. Simen-Kapeu A, Seale AC, Wall S, Nyange C, Qazi SA, Moxon SG, Young M, Liu G, Darmstadt GL, Dickson KE *et al*: **Treatment of neonatal infections: a multi-country analysis of health system bottlenecks and potential solutions.** *BMC Pregnancy and Childbirth* 2015, **15**(2):S6.
15. Oko C, Yusuf A, Taib F: **Hand Hygiene Compliance during COVID-19 Pandemic among Neonatal Nurses in a Federal University Teaching Hospital in Nigeria.** *Malaysian Journal of Medicine and Health Sciences* 2022, **18**:119-127.
16. Herbeć A, Chimhini G, Rosenberg-Pacareu J, Sithole K, Rickli F, Chimhuya S, Manyau S, Walker AS, Klein N, Lorencatto F *et al*: **Barriers and facilitators to infection prevention and control in a neonatal unit in Zimbabwe – a theory-driven qualitative study to inform design of a behaviour change intervention.** *Journal of Hospital Infection* 2020, **106**(4):804-811.
17. Gephart SM, Quinn MC: **Relationship of Necrotizing Enterocolitis Rates to Adoption of Prevention Practices in US Neonatal Intensive Care Units.** *Adv Neonatal Care* 2019, **19**(4):321-332.

18. Dawczynski K, Schleußner E, Dobermann H, Proquitté H: **[Infection Prevention in Premature Infants and Newborns in Thuringia: Implementation of Recommendation of the Commission for Hospital Hygiene and Infection Prevention (KRINKO)]**. *Z Geburtshilfe Neonatol* 2017, **221**(1):30-38.
19. Dumpa V, Adler B, Allen D, Bowman D, Gram A, Ford P, Sannoh S: **Reduction in Central Line-Associated Bloodstream Infection Rates After Implementations of Infection Control Measures at a Level 3 Neonatal Intensive Care Unit()**. *Am J Med Qual* 2019, **34**(5):488-493.
20. Wilder KA, Wall B, Haggard D, Epperson T: **CLABSI Reduction Strategy: A Systematic Central Line Quality Improvement Initiative Integrating Line-Rounding Principles and a Team Approach**. *Adv Neonatal Care* 2016, **16**(3):170-177.
21. Theron M, Botma Y, Heyns T: **Infection prevention and control practices of non-medical individuals in a neonatal intensive care unit: A Donabedian approach**. *Midwifery* 2022, **112**:103393.
22. Thomas AM, Kaur S, Biswal M, KL NR, Vig S: **Effectiveness of hand hygiene promotional program based on the WHO multimodal hand hygiene improvement strategy, in terms of compliance and decontamination efficacy in an indian tertiary level neonatal surgical intensive care unit**. *Indian J Med Microbiol* 2019, **37**(4):496-501.
23. Barrett RE, Fleiss N, Hansen C, Campbell MM, Rychalsky M, Murdzek C, Krechevsky K, Abbott M, Allegra T, Blazevich B *et al*: **Reducing MRSA Infection in a New NICU During the COVID-19 Pandemic**. *Pediatrics* 2023, **151**(2).
24. Mahieu L, Van Damme K, Mertens K, Pierart J, Tackoen M, Cossey V: **Compliance with international prevention guidelines for central-line-associated bloodstream infections in neonatal intensive care units in Belgium: a national survey**. *J Hosp Infect* 2022, **129**:49-57.
25. Pasricha S, Valiquette CR, Singh M, Pasricha R, Jimal D, Khurshid F: **Neonatal intensive care unit hand hygiene: Exploring current practice and adherence barriers in a Canadian hospital**. *Canadian Journal of Infection Control* 2021, **36**(2):77-85.
26. Trudel C, Cobb S, Momtahan K, Brintnell J, Mitchell A: **Human factors considerations in designing for infection prevention and control in neonatal care – findings from a pre-design inquiry**. *Ergonomics* 2018, **61**(1):169-184.
27. Chandonnet CJ, Kahlon PS, Rachh P, Degrazia M, Dewitt EC, Flaherty KA, Spigel N, Packard S, Casey D, Rachwal C *et al*: **Health care failure mode and effect analysis to reduce NICU line-associated bloodstream infections**. *Pediatrics* 2013, **131**(6):e1961-1969.
28. Gephart SM, Newnam K, Weiss A, Wyles C, Shea K: **Feasibility and Acceptability of a Neonatal Project ECHO (NeoECHO) as a Dissemination and Implementation Strategy to Prevent Necrotizing Enterocolitis**. *Worldviews Evid Based Nurs* 2021, **18**(6):361-370.
29. Batthula V, Somnath SH, Datta V: **Reducing late-onset neonatal sepsis in very low birthweight neonates with central lines in a low-and-middle-income country setting**. *BMJ Open Qual* 2021, **10**(Suppl 1).
30. Chandonnet CJ, Boutwell KM, Spigel N, Carter J, DeGrazia M, Ozonoff A, Flaherty K: **It's in Your Hands: An Educational Initiative to Improve Parent/Family Hand Hygiene Compliance**. *Dimens Crit Care Nurs* 2017, **36**(6):327-333.
31. Gon G, Ali SM, Towriss C, Kahabuka C, Ali AO, Cavill S, Dahoma M, Faulkner S, Haji HS, Kabole I *et al*: **Unpacking the enabling factors for hand, cord and birth-surface hygiene in Zanzibar maternity units**. *Health Policy Plan* 2017, **32**(8):1220-1228.
32. Salem MR, Youssef MRL: **Health care providers' perspectives for providing quality infection control measures at the neonatal intensive care unit, Cairo University Hospital**. *American Journal of Infection Control* 2017, **45**(9):e99-e102.
33. Stroever S, Boston K, Ellsworth M, Cuccaro P, McCurdy S: **Qualitative process evaluation of a central line-associated bloodstream infection (CLABSI) prevention team in the neonatal intensive care unit**. *American Journal of Infection Control* 2020, **48**(9):987-992.
34. Hawes JA, Lee KS: **Reduction in Central Line-Associated Bloodstream Infections in a NICU: Practical Lessons for Its Achievement and Sustainability**. *Neonatal Netw* 2018, **37**(2):105-115.

35. Zhou J, Chen S: **Knowledge, Attitudes, and Practices of NICU Doctors and Nurses Toward Prevention and Control of Nosocomial Infection With Multidrug Resistant Organism**. *Frontiers in Pediatrics* 2022, **10**.
36. Hayashi M, Grover TR, Small S, Staples T, Roosevelt G: **Improving timeliness of hepatitis B vaccine administration in an urban safety net level III NICU**. *BMJ Qual Saf* 2021, **30**(11):911-919.
37. Hightower HB, Young JA, Thomas J, Smith JJ, Hobby-Noland D, Palombo G, McCaskey M, Benton B, Hutto C, Coghill C *et al*: **Reduction of Central-line-Associated Bloodstream Infections in a Tertiary Neonatal Intensive Care Unit through Simulation Education**. *Pediatr Qual Saf* 2022, **7**(6):e610.
38. Maria A, Mukherjee R, Upadhyay S, Pratima K, Bandyopadhyay T, Gupta R, Dubey B, Sharma A, Mall PK, Sahoo M *et al*: **Barriers and enablers of breastfeeding in mother-newborn dyads in institutional settings during the COVID-19 pandemic: A qualitative study across seven government hospitals of Delhi, India**. *Front Nutr* 2022, **9**:1052340.
39. Weiss AB, Newnam KM, Wyles C, Shea K, Gephart SM: **Exploring Internal Facilitators' Experience With NeoECHO to Foster NEC Prevention and Timely Recognition Through the iPARIHS Lens**. *Adv Neonatal Care* 2021, **21**(6):462-472.
40. Erdei C, McAvoy LL, Gupta M, Pereira S, McGowan EC: **Is zero central line-associated bloodstream infection rate sustainable? A 5-year perspective**. *Pediatrics* 2015, **135**(6):e1485-1493.
41. Azmeraw Getie B, Engida Yismaw A, Eskezia Tiguh A: **Kangaroo mother care knowledge and practice among mothers who gave birth to preterm and low birth weight babies in Amhara regional state referral hospitals, North West Ethiopia**. *International Journal of Africa Nursing Sciences* 2022, **17**:100470.
42. Ceballos K, Waterman K, Hulett T, Makic MB: **Nurse-driven quality improvement interventions to reduce hospital-acquired infection in the NICU**. *Adv Neonatal Care* 2013, **13**(3):154-163; quiz 164-155.
43. Cross S, Afsana K, Banu M, Mavalankar D, Morrison E, Rahman A, Roy T, Saxena D, Vora K, Graham WJ: **Hygiene on maternity units: lessons from a needs assessment in Bangladesh and India**. *Global Health Action* 2016, **9**(1):32541.
44. Dye ME, Pugh C, Sala C, Scott TA, Wallace T, Grubb PH, Hatch LD: **Developing a Unit-Based Quality Improvement Program in a Large Neonatal ICU**. *Jt Comm J Qual Patient Saf* 2021, **47**(10):654-662.
45. Stone S, Lee HC, Sharek PJ: **Perceived Factors Associated with Sustained Improvement Following Participation in a Multicenter Quality Improvement Collaborative**. *Jt Comm J Qual Patient Saf* 2016, **42**(7):309-315.
46. Bierlaire S, Danhaive O, Carkeek K, Piersigilli F: **How to minimize central line–associated bloodstream infections in a neonatal intensive care unit: a quality improvement intervention based on a retrospective analysis and the adoption of an evidence-based bundle**. *European Journal of Pediatrics* 2021, **180**(2):449-460.
47. Aragona E, West D, Loyal J: **Well-Newborn Unit Director Experiences During the COVID-19 Pandemic: A BORN Study**. *Hosp Pediatr* 2021, **11**(9):e170-e181.
48. Manerkar S, Kalamdani P, Patra S, Kalathingal T, Mondkar J: **Improving Early Colostrum Feeding in a Tertiary Neonatal Intensive Care Unit: A Quality Improvement Initiative**. *Breastfeed Med* 2022, **17**(2):143-148.
49. Badparva B, Ghanbari A, Karkhah S, Osuji J, Kazemnejad Leyli E, Jafaraghaee F: **Prevention of central line-associated bloodstream infections: ICU nurses' knowledge and barriers**. *Nurs Crit Care* 2023, **28**(3):419-426.
50. Biswas A, Bhattacharya SD, Singh AK, Saha M: **Addressing Hand Hygiene Compliance in a Low-Resource Neonatal Intensive Care Unit: a Quality Improvement Project**. *J Pediatric Infect Dis Soc* 2019, **8**(5):408-413.
51. Cross S, Gon G, Morrison E, Afsana K, Ali SM, Manjang T, Manneh L, Rahman A, Saxena D, Vora K *et al*: **An invisible workforce: the neglected role of cleaners in patient safety on maternity units**. *Global Health Action* 2019, **12**(1):1480085.

52. Song X, Stockwell DC, Floyd T, Short BL, Singh N: **Improving hand hygiene compliance in health care workers: Strategies and impact on patient outcomes**. *American Journal of Infection Control* 2013, **41**(10):e101-e105.
53. Goodchild L, Hussey L, McPhee AJ, Lizarondo L, Gillis J, Collins CT: **Promoting early expression of breast milk in mothers of preterm infants in a neonatal unit: a best practice implementation project**. *JBI Database System Rev Implement Rep* 2018, **16**(10):2027-2037.
54. Pahwa P, Lunsford S, Livesley N: **Experiences of Indian Health Workers Using WhatsApp for Improving Aseptic Practices With Newborns: Exploratory Qualitative Study**. *JMIR Med Inform* 2018, **6**(1):e13.
55. Steiner M, Langgartner M, Cardona F, Waldhör T, Schwindt J, Haiden N, Berger A: **Significant Reduction of Catheter-associated Blood Stream Infections in Preterm Neonates After Implementation of a Care Bundle Focusing on Simulation Training of Central Line Insertion**. *Pediatr Infect Dis J* 2015, **34**(11):1193-1196.
56. Alimohammadzadeh K, Bahadori M, Jahangir T, Ravangard R: **Assessing Common Medical Errors in a Children's Hospital NICU Using Failure Mode and Effects Analysis (FMEA)**. *Trauma Monthly* 2017, **22**(5):-.
57. Ngugi SK, Murila FV, Musoke RN: **Hand hygiene practices among healthcare workers in a newborn unit of a tertiary referral hospital in Kenya**. *Journal of Infection Prevention* 2019, **20**(3):132-138.
58. Alslaim HS, Chan J, Saleem-Rasheed F, Ibrahim Y, Karabon P, Novotny N: **Discordance among Belief, Practice, and the Literature in Infection Prevention in the NICU**. *Children* 2022, **9**(4):492.
59. Singh M, Agrawal A, Sisodia D, Kasar PK, Kaur A, Datta V, Savanna RS, Singh M, Livesley N: **Supplementing hand washing with proper use of alcoholic hand rub in a special neonatal care unit in a large academic public health institute at Jabalpur, Madhya Pradesh, India**. *BMJ Open Qual* 2021, **10**(4).
60. Shettigar S, Aradhya AS, Ramappa S, Reddy V, Venkatagiri P: **Reducing healthcare-associated infections by improving compliance to aseptic non-touch technique in intravenous line maintenance: a quality improvement approach**. *BMJ Open Quality* 2021, **10**(Suppl 1):e001394.
61. Dramowski A, Aucamp M, Bekker A, Pillay S, Moloto K, Whitelaw AC, Cotton MF, Coffin S: **NeoCLEAN: a multimodal strategy to enhance environmental cleaning in a resource-limited neonatal unit**. *Antimicrobial Resistance & Infection Control* 2021, **10**(1):35.
62. Amaan A, Dey SK, Zahan K: **Improvement of Hand Hygiene Practices among the Healthcare Workers in a Neonatal Intensive Care Unit**. *Can J Infect Dis Med Microbiol* 2022, **2022**:7688778.
63. Bezerra TB, Valim MD, Bortolini J, Ferreira AM, Almeida WA, Rigotti MA, De Andrade D, Fronteira I, Lopes Sousa AF: **Influencing factors of hand hygiene in critical sections of a brazilian hospital**. *J Infect Dev Ctries* 2021, **15**(6):840-846.
