# Supplementary material for: Investigating the implementation of infection prevention and control practices in neonatal care across country income levels: a systematic review
Source: Antimicrob Resist Infect Control. 2025 Feb 7;14:8. doi: 10.1186/s13756-025-01516-7 (PMC11806577; doi:10.1186/s13756-025-01516-7)
Supplement: Supplementary file 4 — Additional File 4: Coding Manual for Implementation Strategies based on Expert Recommendations for Implementing Change. [file 13756_2025_1516_MOESM4_ESM.pdf]

Investigating the Implementation of Infection Prevention and Control Practices in Neonatal Care Across Country Income Levels: A Systematic Review

Emanuela Nyantakyi, Julia Baenziger, Laura Caci, Kathrin Blum, Aline Wolfensberger, Angela Dramowski, Bianca Albers, Marta Castro, Marie-Therese Schultes, Lauren Clack

SUPPLEMENTARY FILE

Coding Manual for Implementation Strategies based on Expert Recommendations for Implementing Change Taxonomoy

Contents

Use evaluative and iterative strategies 3
Provide interactive assistance 8
Adapt and tailor to context 9
Develop relationships 12
Train and educate 16
Support clinicians 19
Engage involved individuals or groups 21
Utilize financial/incentivizing strategies 22
Change infrastructure 24
References 26

Use evaluative and iterative strategies

ERIC strategy	Definition	Example
**Assess for readiness (and identify barriers and facilitators)**<br>*Note: Emphasis must really be on (organizational) readiness*	Assess various aspects of an organization to determine its degree of readiness to implement, barriers that may impede implementation, and strengths that can be used in the implementation effort	
**Audit and provide feedback**	Collect and summarize clinical performance data (e.g., IPC rates, compliance rates) over a specified time period and give it to clinicians and administrators to monitor, evaluate, and modify provider behavior	*Encouragement was provided to bedside clinicians through random chart audits from June 2016–May 2017, praising staff that coordinated care, providing performance recognition at staff meetings, and reminding staff who did not through friendly face to face interaction and email of the importance of routine, coordinated oral care [1]*<br><br>*The monthly results of cost consumption and HCAI rates were displayed on the hospital notice [2]*<br><br>*The rates of adherence to the WHO guideline on Hand Hygiene were monitored and included in the feedback to the units throughout the outbreak period. [3]*
**Purposefully reexamine the implementation**<br>*Note: Assessment during/after practice has been implemented*	Monitor progress and adjust clinical practices and implementation strategies to continuously improve the quality of care	*The Trust introduced a venous infusion phlebitis score and required that the condition of cannula sites was recorded in the medical records each day, so that early signs of phlebitis were noted and appropriate action was taken. [4]*<br><br>*In addition, at every month-end, the online laboratory data of each patient admitted was*

ERIC strategy	Definition	Example
		reviewed for any positive culture reports that were missed [5]
**Develop and implement tools for quality monitoring**<br>*Note: Emphasis on* <u>*introduction*</u> *of a QM tool/system*	Develop, (test,) and introduce into quality-monitoring systems the right input—the appropriate language, protocols, algorithms, standards, and measures (of processes, patient/consumer outcomes, and implementation outcomes) that are often specific to the innovation being implemented	*The Trust introduced a venous infusion phlebitis score and required that the condition of cannula sites was recorded in the medical records each day, so that early signs of phlebitis were noted and appropriate action was taken. [4]*<br><br>*The process indicators were based on hand hygiene (30 audits per month) and central line care audits (10 audits per month). If all the steps of hand hygiene including the six core steps and the duration were correctly performed, it was considered 'overall compliant to HH'. [5]*<br><br>*The housekeeping supervisor uses a daily cleaning checklist to ensure that all the areas are cleaned. A color-coding system is used with separate cloths for patient, toilet, isolation and kitchen areas [6]*
**Develop and organize quality monitoring systems**<br>*Note: Emphasis on* <u>*organization/process*</u> *of a QM system or procedure*	Develop and organize systems and procedures (protocols, algorithms, standards, and measures (of processes, patient/consumer outcomes, and implementation outcomes) that monitor clinical processes and/or outcomes for the purpose of quality assurance and improvement	*Regular documentation of the number of young infants in the NICU, those with central lines, and those on antibiotics, invasive ventilation, non-invasive ventilation, and parenteral nutrition was strengthened. The data was cross-verified on a periodic basis [5]*
**Update tools/systems for quality monitoring**<br>*(added)*	Update systems and procedures (protocols, algorithms, standards, and measures (of processes, patient/consumer outcomes, and implementation outcomes) that monitor	*Hospital-wide blood culture guidelines updated and re-emphasized [4])*

ERIC strategy	Definition	Example
	clinical processes and/or outcomes for the purpose of quality assurance and improvement	
**Develop a formal implementation blueprint**	Develop a formal implementation blueprint that includes all goals and strategies. The blueprint should include the following: 1) aim/purpose of the implementation; 2) scope of the change (*e.g.*, what organizational units are affected); 3) timeframe and milestones; and 4) appropriate performance/progress measures. Use and update this plan to guide the implementation effort over time	*The NICU's multidisciplinary leadership team decided to pursue obtaining the HMB-HMF for premature infants with a birth weight 1250 g or less and organized a methodical approach to implementation, financial sustainability, and clinical outcomes tracking. [7]*
**Conduct local needs assessment**<br>*Note: Usually assessment of barriers and/or facilitators to understand current practice and need (often before rollout of program)*	Collect and analyze data related to the need for the innovation	*Root cause analysis for the CLABSI in the unit identified various causes (Web Fig. 1), based on which, the QI initiative focused on hand hygiene (HH) and care bundle approach to central line care. [5]*
**Stage implementation scale up**	Phase implementation efforts by starting with small pilots or demonstration projects and gradually move to a system wide rollout	*Observation audits were conducted during the week of the educational intervention. After a break for 1 week, the timer phase was implemented. This phase involved surveying nurses as to how they estimated scrub times, and implementation of bedside timers to improve scrub time accuracy for both venous catheters and feeding tubes. Two types of timers were trialed: actual timers and music buttons. Music buttons played 15 seconds of "Twinkle Little Star." For standardization, the timers were set for 15 seconds. For both devices, the nurse was required to press the device twice– once for each alcohol swab–to achieve the total 30-second scrub time. The timers and music buttons were mounted on*

ERIC strategy	Definition	Example
		laminated index cards with instructions on their use, and were placed ran-domly at the bedsides of the 20 intensive care beds (available on request) [8]<br><br>*The months of September and October served as an opportunity for HCPs to develop experience and comfort with the HFZs; during these initial months, no surveys were distributed. During November and December 2015, the same HCPs and patient family surveys were distributed as before the HFZs were established. Survey responses were subsequently evaluated and compared [9]*
**Obtain and use patients/consumers and family feedback**	Develop strategies to increase patient/consumer and family feedback on the implementation effort	**Consumers = HCWs**<br>*In January 2020, after feedback from NICU and infection prevention leadership, a new disinfection policy was implemented [10]*<br><br>*Subsequently a feedback meeting to present the audit results was conducted in August 2014, the implementation process was discussed to propose new knowledge transfer strategies to achieve the goals [11]*
**Conduct cyclical small tests of change**	Implement changes in a cyclical fashion using small tests of change before taking changes system-wide. Tests of change benefit from systematic measurement, and results of the tests of change are studied for insights on how to do better. This process continues serially over time, and refinement is added with each cycle	*To ensure 20-30 seconds of hand rub, recitation of a nursery rhyme or doing each step 8 times were tried. However these were quickly abandoned, as they did not ensure 20 seconds of hand rub. The successful PDSA cycle was to do the hand rub by the clock for 20-30 seconds. It was ensured that a clock with a second hand was easily visible from each bed of the unit. [5]*

ERIC strategy	Definition	Example
		With no significant changes to the rate of HCAI, the team decided to extend the use of hypochlorite solution to three shifts and continue with glutaraldehyde in one shift in the second PDSA (2 August 2021–31 August 2021). [2]

Provide interactive assistance

ERIC strategy	Definition	Example
**Facilitation**	A process of interactive problem solving and support that occurs in a context of a recognized need for improvement and a supportive interpersonal relationship	*Creation of a WhatsApp group of the QI team and nurses of NICU. This was started by the QI team leader and it was planned to create awareness regarding CLABSI bundle on this group [12]*
**Provide local technical assistance**	Develop and use a system to deliver technical assistance focused on implementation issues using local personnel	*The availability of specialist expertise (such as a vascular access nurse) [4]*<br><br>*Trained and designated QI nurses were initially involved in this process, however due to lack of coverage on all days; this was abandoned [5]*<br><br>*We clarified verbal consent was sufficient by the hospital legal team. The parent consenting would be given the Vaccine Information Sheet (VIS) and this was to be documented in the Medication Administration Record by the nurse. In situations where biological mothers were not available to consent, we received approval for the other parent to consent [13]*
**Provide clinical supervision**	Provide clinicians with ongoing supervision focusing on the innovation. Provide training for clinical supervisors who will supervise clinicians who provide the innovation	*A senior nurse or doctor supervised the process of insertion using a checklist and any deviation from the policy was noted and stopped promptly [5]*
**Centralize technical assistance**	Develop and use a centralized system to deliver technical assistance focused on implementation issues	*The QI project was registered with the hospital-wide online "QI PORTAL," and all project members underwent a standard duration of training in preparation. [14]*

Adapt and tailor to context

ERIC strategy	Definition	Example
**Tailor strategies**	Tailor the implementation strategies to address barriers and leverage facilitators that were identified through earlier data collection	*For the initial training, the PowerPoint presentation was delivered in a classroom setting. However, due to shift scheduling it was impossible to gather all NICU staff together for a single training, so the presentation was also recorded to allow staff to take the training when scheduling was convenient. [15]*<br><br>*In the first step and after consulting with the NICU experts, 4 key processes were selected through studying the care processes and brainstorming including drug administration, infection control, medical equipment use, and laboratory tests. In the second and third steps, 27 activities of these processes and 50 potential failure modes, as well as their effects were detected and recorded in the final worksheet of FMEA. In the fourth step, the PRNs were calculated and given the range of 1 < RPN < 130, and the ranking scale of 1 to 10 for 3 indicators mentioned above, 27 potential failure modes with PRN > 65 were determined as the high risk failures, and were listed in the final work-sheet. In the fifth step, their causes were determined us-ng the team members' opinion and recorded in the root causes column of FMEA worksheet. Finally, suggestions for reducing the probability of error occurrence, decreasing the error severity, and increasing the probability of error detection were*

ERIC strategy	Definition	Example
		offered according to the scores of D, O, and S indicators. [16]
**Promote adaptability**	Identify the ways a clinical innovation can be tailored to meet local needs and clarify which elements of the innovation must be maintained to preserve fidelity	*When Clinell wipes were out of stock, staff were trained to use alternative cleaning agents e.g. cleaning with liquid detergent and water and drying, followed by disinfection with 70% alcohol spray* [6]<br><br>*HCPs explained the concept and rationale of HFZs to patient families. The HFZs were promoted as a means to decrease the spread of hospital-acquired disease by bringing attention to the importance of hands as vectors for disease, by emphasizing the importance of hand hygiene, by discouraging the handshake, and by encouraging alternative gestures (such as touching someone's shoulder, Namaste, or fist-bump) and other nonverbal forms of communication (such as eye contact, smiles, and asking about other's well-being)* [9]
**Use data experts**	Involve, hire, and/or consult experts to inform management on the use of data generated by implementation efforts	
**Use data warehousing techniques**	Integrate clinical records across facilities and organizations or teams to facilitate implementation across systems	*Completed INICC process surveillance forms for hand hygiene were sent monthly by ICT members from each participating ICU to the INICC headquarters. The team at the INICC headquarters uploaded the data into a database, analyzed the data, and sent to ICT members of each participating ICU a report on hand hygiene compliance showing hand hygiene compliance by*

ERIC strategy	Definition	Example
		month, sex, HCW profession, ICU, work shift, and type of contact. [17]<br><br>*[W]e developed a secure, Web-based data infrastructure for our teams using the Research Electronic Data Capture (REDCap) program housed at our medical center. (...) In this way, clinical information is manually entered into the database only one time and then shared by multiple teams [18]*

Develop relationships

ERIC strategy	Definition	Example
**Identify and prepare champions**	Identify and prepare individuals who dedicate themselves to supporting, marketing, and driving through an implementation, overcoming indifference or resistance that the intervention may provoke in an organization	*"Just in Time" performance feedback was provided by HH champions on all noncompliant HH moments and infection control practices. [14]*
**Organize clinician implementation team meetings**	Develop and support teams of clinicians who are implementing the innovation and give them protected time to reflect on the implementation effort, share lessons learned, and/or support one another's learning	*At the beginning of our program, no faculty or staff member received salary support or had protected time for QI. As the program grew, hospital support for the QI pro-gram increased. Currently, two of the QI leadership team members receive limited salary support for leading QI in the NICU. Dedicated time for QI leadership enabled individual team mentoring to continue as our QI program and project teams grew. [18]*<br><br>*The NICU interdisciplinary QI team met twice monthly to develop strategies for improving CLABSI and VAP. [19]*
**Recruit, designate, and train for leadership**	Recruit, designate, and train leaders for the change effort	
**Inform local opinion leaders**	Inform providers identified by colleagues as opinion leaders or "educationally influential" about the clinical innovation in the hopes that they will influence colleagues to adopt it	
**Build a coalition**	Recruit and cultivate relationships with partners in the implementation effort	*Partnering with EVS allowed us to: (1) provide education about MRSA in the NICU, (2) share baseline fluorescent marking data to support the need for improvement [...] [10]*

ERIC strategy	Definition	Example
		The trainers' team included Infection control specialists from the Palestinian Ministry of Health. [20]
**Obtain formal commitments**	Obtain written commitments from key partners that state what they will do to implement the innovation	
**Identify early adopters**	Identify early adopters at the local site to learn from their experiences with the practice innovation	
**Conduct local consensus discussions**	Include local providers and other stakeholders in discussions that address whether the chosen problem is important and whether the clinical innovation to address it is appropriate	*Since over half of the infants screened positive for VRE-fm, after consulting with the MoH [Ministry of Health], it was decided that positive and exposed infants (i.e., all infants in the NICU at this time point) will comprise a single cohort and be treated according to the same protocol [3]*<br><br>*In this step, the team members determined the potential failure modes of each process steps or components of the system using brain storming, and recorded them in the related form.*<br>*5. Determining the Causes of Each Failure and Error: In this step, the team members determined the causes leading to the failure modes using brainstorming, and cause and effect diagrams. [16]*
**Capture and share local knowledge**	Capture local knowledge from implementation sites on how implementers and clinicians made something work in their setting and then share it with other sites	*In addition, the business proposal also included a list of other institutions having implemented an EHMD. [7]*

ERIC strategy	Definition	Example
**Use advisory boards and workgroups**	Create and engage a formal group of multiple kinds of stakeholders to provide input and advice on implementation efforts and to elicit recommendations for improvements	*The consumer advisory group involved 10 mothers of premature infants who had been dis-charged from the WCH unit in the previous 12 months and were in the hospital attending follow-up appointments for their babies [21]*
**Use an implementation advisor**	Seek guidance from experts in implementation	
**Model and simulate change**	Model or simulate the change that will be implemented prior to implementation	
**Visit other sites**	Visit sites where a similar implementation effort has been considered successful	*[S]end nurses to other hospital units to observe proper aseptic techniques [22]*
**Involve executive boards**	Involve existing governing structures (*e.g.*, boards of directors, medical staff boards of governance) in the implementation effort, including the review of data on implementation processes	*[T]he clear commitment of management across the Trust [4]*<br><br>*Compliance data were presented, stratified by role, and shared with applicable leadership as a mechanism for comparison, feedback, and intervention. [10]*<br><br>*Monthly reporting of data to NICU faculty and quarterly to service line chief. [23]*
**Develop an implementation glossary**	Develop and distribute a list of terms describing the innovation, implementation, and stakeholders in the organizational change	
**Develop academic partnerships**	Partner with a university or academic unit for the purposes of shared training and bringing research skills to an implementation project	
**Promote network weaving**	Identify and build on existing high-quality working relationships and networks within and outside the organization, organizational units, teams, etc. to promote information	*To ensure that teams were able to leverage all available resources for QI, including benchmarking data, peer mentoring, and group learning, the QI leadership team, in consultation with hospital and*

ERIC strategy	Definition	Example
	sharing, collaborative problem-solving, and a shared vision/goal related to implementing the innovation	*NICU senior leadership, attempted to align existing teams with ongoing ex-ternal quality and safety initiatives when available. Decisions on external collaboration were driven by unit goals informed by the NICU QI Dashboard. Examples of this included participation of the NICU Nutrition team in the Tennessee Initiative for Perinatal Quality Care (TIPQC),20 our Infection Prevention and Mechanical Ventilation teams with initiatives through the Solutions for Patient Safety (SPS),21 and our Antibiotic Stewardship team in the Vermont Oxford Network [18]*<br><br>*The group also is part of the New Jersey NICU collaborative, the focus of which is to foster QI in the care of NICU patients, including decreasing CLABSI rates in neonates. The study NICU also belongs to the national catheter-associated bloodstream infection (NCABSI) collaborative, which focuses on prevention of CLABSIs [24]*
**Promote/Lobby for interests*** *(added)*	Politically campaign or petition for change/awareness	*HCI petitioned MINSA to include alcohol gel for hand hygiene on its basic supplies list so that it would be distributed to all health facilities nationally. [25]*

Train and educate

ERIC strategy	Definition	Example
**Conduct ongoing training**	Plan for and conduct training in the clinical innovation in an ongoing way	
**Provide ongoing consultation**	Provide ongoing consultation with one or more experts in the strategies used to support implementing the innovation	*ICT members had continuous telephone or e-mail access to a support team at the INICC headquarters. [17]*
**Develop educational materials**	Develop and format manuals, toolkits, and other supporting materials in ways that make it easier for stakeholders to learn about the innovation and for clinicians to learn how to deliver the clinical innovation	*An oral care poster was created (...) [1]*<br><br>*The Trust sponsored a video on aseptic no touch technique (ANTT) [4]*
**Update educational materials** *(added)*	Update/change educational materials (including guidelines, manuals, and toolkits)	*Hospital-wide blood culture guidelines updated and re-emphasized [4]*
**Make training dynamic**	Vary the information delivery methods to cater to different learning styles and work contexts, and shape the training in the innovation to be interactive	*All the HCPs were educated about HH through posters, regular classes and one to one communication [5]*
**Distribute educational materials**	Distribute educational materials (including guidelines, manuals, and toolkits) in person, by mail, and/or electronically	*The slide show was sent to all NICU RN's through email, and was required as annual mandatory education for all RT's. [1]*
**Use train-the-trainer strategies**	Train designated clinicians or organizations to train others in the clinical innovation	*The Trust (...) initiated a train-the-trainer program. [4]*<br><br>*Recognizing the importance of rigorous QI methods, our goal was to first obtain formal QI training for the QI leadership team, and then use the leadership team to strategically build QI knowledge within the individual QI teams. Over a three-year period, all members of the QI leadership team completed either*

ERIC strategy	Definition	Example
		extra-mural or intramural mentored QI courses to gain expertise in performing rigorous QI work [18]
**Conduct educational meetings or provide trainings**	Hold meetings targeted toward (different) stakeholder groups (*e.g.*, providers, administrators, other organizational stakeholders, and community, patient/consumer, and family stakeholders) to teach them about the clinical innovation	*The centerpiece of the educational program consisted of a 1-hr mandatory slide presentation presented in June as part of annual mandatory education. [1]*<br><br>*Implementation was initiated with multiple staff meetings where NICU leadership explained the rationale for the new policy and answered questions [10]*<br><br>*The education of newborn service and visiting hospital staff now centered around hand hygiene and on the potential role of healthcare workers in the transmission of VRE-fm. [3]*
**Conduct educational outreach visits**	Have a trained person meet with providers in their practice settings to educate providers about the clinical innovation with the intent of changing the provider's practice	*The trainers' team included Infection control specialists from the Palestinian Ministry of Health. [20]*<br><br>*Clinical Practice Improvement Workshop, with a focus on Infection Reduction in Neonates, presented by Clinical Excellence Commission. Workshop attended by medical and nursing representatives from all participating hospitals. [26]_Supplement*
**Create a learning collaborative**	Facilitate the formation of groups of providers or provider organizations and foster a collaborative learning environment to improve implementation of the clinical innovation	*SPRING membership comprised at least one neonatologist and clinical nurse consultant from each NICU. SPRING meetings occurred four to six times per year and provided a forum to review best practice related to infection prevention, develop*

ERIC strategy	Definition	Example
		standard practices and teaching resources, and share ideas on local QI initiatives. Clinical practice improvement (CPI) skills of SPRING participants were also enhanced through participation in a CPI workshop. SPRING activities included development of a potentially better practices framework (box 1) with agreement by all NICUs to implement major components of the framework; implementation of a standardised approach to insertion and maintenance of peripherally inserted central catheters (PICC); production of an educational video for neonatal PICC insertion, with on-line access for all NICU staff; implementation of Hand Hygiene Australia: '5 Moments for Hand Hygiene' and exchange of practical ideas to reduce infection. [26]
**Shadow other experts**	Provide ways for key individuals to directly observe experienced people engage with or use the targeted practice change/innovation	*[S]end nurses to other hospital units to observe proper aseptic technique [22]*
**Work with educational institutions**	Encourage educational institutions to train clinicians in the innovation	

Support clinicians

ERIC strategy	Definition	Example
**Facilitate relay of clinical data to providers**	Provide as close to real-time data as possible about key measures of process/outcomes using integrated modes/channels of communication in a way that promotes use of the targeted innovation	*The central line care audits focused on insertion practices (number of central lines inserted by eligible Healthcare Personnel (HCP), checklist analysis) and maintenance practices (breaks in circuit, 2 HCPs handling the central line, scrubbing the hub for 15 seconds, 2% chlorhexidine used for scrub, use of single lumen central line and needleless connectors). This data was shared with all HCPs in the monthly meetings [5]*<br><br>*A central line card was used to note the number of times/day the circuit was breached. It also served as a constant reminder to remove the line. [5]*
**Remind clinicians or other involved groups**	Develop reminder systems designed to help clinicians to recall information and/or prompt them to use the clinical innovation	*An oral care poster was created and presented to both RT and NICU RN staff at department meetings in May, and then placed on the breast milk refrigerator as a reminder [1]*<br><br>*A central line card was used to note the number of times/day the circuit was breached. It also served as a constant reminder to remove the line. [5]*
**Develop resource sharing agreements**	Develop partnerships with organizations that have resources needed to implement the innovation	
**Revise professional roles**	Shift and revise roles among professionals who provide care, and redesign job characteristics	*Data collection responsibility was expanded to multiple trained nurses. However as it was voluntary and some considered this data collection 'not so important' this was abandoned as well [5]*

ERIC strategy	Definition	Example
		NICU infection control nurse was relieved of all the primary patient care duties with the sole responsibility to lead the NICU infection prevention and control. [23]
**Hire new or additional staff**	Bringing in and onboarding new or additional staff	*The essential goal of reestablishing a favorable patient-to-nurse ratio according to the guidelines of the German society for neonatology and pediatric intensive care (GNPI) was achieved by reemployment of qualified NICU nurses [27]*
**Create new clinical teams**	Change who serves on the clinical team, adding different disciplines and different skills to make it more likely that the clinical innovation is delivered (or is more successfully delivered)	*[A] multi-disciplinary task force was formed, consisting of Respiratory Therapy (RT) and Nursing (RN) educators, Lactation, and unit leaders, to design an educational program that ensure standardized education on VAP reduction and oral care to all staff [1]*

Engage involved individuals or groups

ERIC strategy	Definition	Example
**Involve patients/consumers and family members**	Engage or include patients/consumers and families in the implementation effort	*The consumer advisory group involved 10 mothers of premature infants who had been dis-charged from the WCH unit in the previous 12 months and were in the hospital attending follow-up appointments for their babies [21]*<br><br>*All parents were informed by the attending physicians in the NICU about the outbreak and provided with a written explanation. Information was provided to parents and visitors about the requirement of hand hygiene and the reasons for the enhanced infection control measures for VRE-fm colonized babies. [3]*
**Intervene with patients/consumers to enhance uptake and adherence**	Develop strategies with patients to encourage and problem solve around adherence	
**Prepare patients/consumers to be active participants**	Prepare patients/consumers to be active in their care, to ask questions, and specifically to inquire about care guidelines, the evidence behind clinical decisions, or about available evidence-supported treatments	
**Increase demand**	Attempt to influence the market for the clinical innovation to increase competition intensity and to increase the maturity of the market for the clinical innovation	
**Use mass media**	Use media to reach large numbers of people to spread the word about the clinical innovation	

Utilize financial/incentivizing strategies

ERIC strategy	Definition	Example
**Fund and contract for the clinical innovation**	Governments and other payers of services issue requests for proposals to deliver the innovation, use contracting processes to motivate providers to deliver the clinical innovation, and develop new funding formulas that make it more likely that providers will deliver the innovation	
**Access new funding**	Access new or existing money to facilitate the implementation	*The Trust sponsored a video on aseptic no touch technique (ANTT) (…) [4]*
**Place innovation on fee for service lists/formularies**	Work to place the clinical innovation on lists of actions for which providers can be reimbursed (*e.g.*, a drug is placed on a formulary, a procedure is now reimbursable)	*Next, the team met with representatives from the finance department to determine the most accurate and appropriate way to create a charge code for these medically indicated products. Revenue code 270, a supply code, was determined to be the best fit for the product which identifies the HMB-HMF as a biological supplement for high-risk infants. [7]*
**Alter incentive/allowance structures** *(updated definition)*	Work to (tangibly or intangibly) incentivize the adoption and implementation of the clinical innovation	*[C]hampion of the month award [23]*<br><br>*[B]uilding motivation through encouragement and appreciation (phase-III; 5 bedside nurses/residents every week were given a token gift and a certificate of appreciation on ward rounds every Monday) [28]*
**Make billing easier**	Make it easier to bill for the clinical innovation	
**Alter patient/consumer fees**	Create fee structures where patients/consumers pay less for preferred treatments (the clinical innovation) and more for less-preferred treatments	

ERIC strategy	Definition	Example
**Use other payment schemes**	Introduce payment approaches (in a catch-all category)	
**Develop disincentives**	Provide tangible or intangible disincentives for failure to implement or use the clinical innovation	
**Use capitated payments**	Pay providers or care systems a set amount per patient/consumer for delivering clinical care	

Change infrastructure

ERIC strategy	Definition	Example
**Mandate change**	Have leadership declare the priority of the innovation and their determination to have it implemented	*Involvement in QI and safety programs designed to improve clinical care is an expectation of all the staff members [29]*
**Change record systems**	Change records systems to allow better assessment of implementation or clinical outcomes	*The Trust introduced a venous infusion phlebitis score and required that the condition of cannula sites was recorded in the medical records each day, so that early signs of phlebitis were noted and appropriate action was taken. [4]*<br><br>*This charge code was linked to the nursing flow sheet. Upon taking 1 unit of HMB-HMF from the freezer and preparing the batch of fortified milk, the bedside nurse documented this in the electronic medical record (EMR) flow sheet for infant feeding. This process allowed the NICU to capture the actual cost assigned to the product with charge appropriated to the correct patient. Audits by nurse managers ensured accuracy [7]*
**Change physical structure and equipment**	Evaluate current configurations and adapt, as needed, the physical structure and/or equipment (*e.g.*, changing the layout of a room, adding equipment) to best accommodate the targeted innovation	*Small 3 ml sterile water vials were purchased for oral care use and staff taught to recognize the difference in vials from the saline vial. [1]*<br><br>*Systems wash fluids started to be mounted in a closed circuit, always ready to be used after drug administration, without further handling of the access point, which are now disinfected with vigorous friction with chlorhexidine before and after manipulation. [30]*

ERIC strategy	Definition	Example
**Create or change credentialing and/or licensure standards**	Create an organization that certifies clinicians in the innovation or encourage an existing organization to do so. Change governmental professional certification or licensure requirements to include delivering the innovation. Work to alter continuing education requirements to shape professional practice toward the innovation	*Finally, signed statement from each staff member acknowledging their understanding of the policy and the mandate to comply with it was taken, to ensure the connection between policy and practice. [31]*
**Change service sites**	Change the location of clinical service sites to increase access	*The move to new building resulted in increased space between cots, better air handling, improved water quality and surfaces that facilitated cleaning [4]*
**Change accreditation or membership requirements**	Strive to alter accreditation standards so that they require or encourage use of the clinical innovation. Work to alter membership organization requirements so that those who want to affiliate with the organization are encouraged or required to use the clinical innovation	*[O]ther staff had only informal QI experience or self-directed online training. Recognizing the im-portance of rigorous QI methods, our goal was to first obtain formal QI training for the QI leadership team, and then use the leadership team to strategically build QI knowledge within the individual QI teams. Over a three-year period, all members of the QI leadership team completed either extra-mural or intramural mentored QI courses to gain expertise in performing rigorous QI work. [18]*
**Start a dissemination organization**	Identify or start a separate organization that is responsible for disseminating the clinical innovation. It could be a for-profit or non-profit organization	
**Change liability laws**	Participate in liability reform efforts that make clinicians more willing to deliver the clinical innovation	

References

1. Lauderbaugh D, Holub P, Turner K, Popien T: **Reducing Ventilator Associated Pneumonia in the NICU through oral care education: A quality improvement project.** *Journal of Neonatal Nursing* 2019, **25**(3):127-129.
2. Somasekhara Aradhya A, Mercy L, Reddy V, Venkatagiri P: **Reducing the costs of floor cleaning in a level III NICU of Bangalore rural: a quality.** *BMJ Open Qual* 2022, **11**(Suppl 1).
3. Marom R, Mandel D, Haham A, Berger I, Ovental A, Raskind C, Grisaru-Soen G, Adler A, Lellouche J, Schwartz D *et al*: **A silent outbreak of vancomycin-resistant Enterococcus faecium in a neonatal intensive care unit.** *Antimicrobial Resistance & Infection Control* 2020, **9**(1):87.
4. Sinha AK, Murthy V, Nath P, Morris JK, Millar M: **Prevention of Late Onset Sepsis and Central-line Associated Blood Stream Infection in Preterm Infants.** *Pediatr Infect Dis J* 2016, **35**(4):401-406.
5. Balla KC, Rao SP, Arul C, Shashidhar A, Prashantha YN, Nagaraj S, Suresh G: **Decreasing Central Line-associated Bloodstream Infections Through Quality Improvement Initiative.** *Indian Pediatr* 2018, **55**(9):753-756.
6. Dramowski A, Aucamp M, Bekker A, Pillay S, Moloto K, Whitelaw AC, Cotton MF, Coffin S: **NeoCLEAN: a multimodal strategy to enhance environmental cleaning in a resource-limited neonatal unit.** *Antimicrobial Resistance & Infection Control* 2021, **10**(1):35.
7. Delaney Manthe E, Perks PH, Swanson JR: **Team-Based Implementation of an Exclusive Human Milk Diet.** *Adv Neonatal Care* 2019, **19**(6):460-467.
8. Caspari L, Epstein E, Blackman A, Jin L, Kaufman DA: **Human factors related to time-dependent infection control measures: "Scrub the hub" for venous catheters and feeding tubes.** *Am J Infect Control* 2017, **45**(6):648-651.
9. Parga JJ, Valadez M, Chang R-KR, Sarin-Gulian A, Holdbrooks H, Sklansky MS: **Handshake-free zone in a neonatal intensive care unit: Initial feasibility study.** *American Journal of Infection Control* 2017, **45**(7):787-792.
10. Barrett RE, Fleiss N, Hansen C, Campbell MM, Rychalsky M, Murdzek C, Krechevsky K, Abbott M, Allegra T, Blazevich B *et al*: **Reducing MRSA Infection in a New NICU During the COVID-19 Pandemic.** *Pediatrics* 2023, **151**(2).
11. Ramos Ferreira Curan G, Giovanini Rossetto E, Corrêa Castral T: **Using the knowledge translation framework to change practical care of central catheters in a Brazilian neonatal unit.** *J Infect Dev Ctries* 2017, **11**(6):445-452.
12. Batthula V, Somnath SH, Datta V: **Reducing late-onset neonatal sepsis in very low birthweight neonates with central lines in a low-and-middle-income country setting.** *BMJ Open Qual* 2021, **10**(Suppl 1).
13. Hayashi M, Grover TR, Small S, Staples T, Roosevelt G: **Improving timeliness of hepatitis B vaccine administration in an urban safety net level III NICU.** *BMJ Qual Saf* 2021, **30**(11):911-919.
14. Bharadwaj S, Ho SK, Khong KC, Seet A, Yeo KC, Chan XY, Wong LL, Karlin RB, Chan DK, Ling ML: **Eliminating MRSA transmission in a tertiary neonatal unit-A quality improvement initiative.** *Am J Infect Control* 2019, **47**(11):1329-1335.
15. Kallam B, Pettitt-Schieber C, Owen M, Agyare Asante R, Darko E, Ramaswamy R: **Implementation science in low-resource settings: using the interactive systems framework to improve hand hygiene in a tertiary hospital in Ghana.** *International Journal for Quality in Health Care* 2018, **30**(9):724-730.
16. Alimohammadzadeh K, Bahadori M, Jahangir T, Ravangard R: **Assessing Common Medical Errors in a Children's Hospital NICU Using Failure Mode and Effects Analysis (FMEA).** *Trauma Monthly* 2017, **22**(5):-.

17. Rosenthal VD, Pawar M, Leblebicioglu H, Navoa-Ng JA, Villamil-Gómez W, Armas-Ruiz A, Cuéllar LE, Medeiros EA, Mitrev Z, Gikas A *et al*: **Impact of the International Nosocomial Infection Control Consortium (INICC) Multidimensional Hand Hygiene Approach over 13 Years in 51 Cities of 19 Limited-Resource Countries from Latin America, Asia, the Middle East, and Europe**. *Infection Control & Hospital Epidemiology* 2013, **34**(4):415-423.
18. Dye ME, Pugh C, Sala C, Scott TA, Wallace T, Grubb PH, Hatch LD: **Developing a Unit-Based Quality Improvement Program in a Large Neonatal ICU**. *Jt Comm J Qual Patient Saf* 2021, **47**(10):654-662.
19. Ceballos K, Waterman K, Hulett T, Makic MB: **Nurse-driven quality improvement interventions to reduce hospital-acquired infection in the NICU**. *Adv Neonatal Care* 2013, **13**(3):154-163; quiz 164-155.
20. Alrumi N, Aghaalkurdi M, Habib H, Abed S, Böttcher B: **Infection control measures in neonatal units: implementation of change in the Gaza-Strip**. *The Journal of Maternal-Fetal & Neonatal Medicine* 2020, **33**(20):3490-3496.
21. Goodchild L, Hussey L, McPhee AJ, Lizarondo L, Gillis J, Collins CT: **Promoting early expression of breast milk in mothers of preterm infants in a neonatal unit: a best practice implementation project**. *JBI Database System Rev Implement Rep* 2018, **16**(10):2027-2037.
22. Gafirimbi N, Wong R, Adomako E, Kagwiza J: **Lessons learned in establishing a quality improvement project to reduce hospital acquired infections in the neonatology ward at a referral hospital in Rwanda**. *On the Horizon* 2016, **24**(4):341-348.
23. Hussain AS, Ahmed AM, Arbab S, Ariff S, Ali R, Demas S, Zeb J, Rizvi A, Saleem A, Farooqi J: **CLABSI reduction using evidence based interventions and nurse empowerment: a quality improvement initiative from a tertiary care NICU in Pakistan**. *Arch Dis Child* 2021, **106**(4):394-400.
24. Dumpa V, Adler B, Allen D, Bowman D, Gram A, Ford P, Sannoh S: **Reduction in Central Line-Associated Bloodstream Infection Rates After Implementations of Infection Control Measures at a Level 3 Neonatal Intensive Care Unit()**. *Am J Med Qual* 2019, **34**(5):488-493.
25. López S, Wong Y, Urbina L, Gómez I, Escobar F, Tinoco B, Parrales A: **Quality in practice: preventing and managing neonatal sepsis in Nicaragua**. *Int J Qual Health Care* 2013, **25**(5):599-605.
26. Bowen JR, Callander I, Richards R, Lindrea KB: **Decreasing infection in neonatal intensive care units through quality improvement**. *Archives of Disease in Childhood - Fetal and Neonatal Edition* 2017, **102**(1):F51-F57.
27. Hensel KO, van den Bruck R, Klare I, Heldmann M, Ghebremedhin B, Jenke AC: **Nursing staff fluctuation and pathogenic burden in the NICU - effective outbreak management and the underestimated relevance of non-resistant strains**. *Scientific Reports* 2017, **7**(1):45014.
28. Gopalakrishnan S, Chaurasia S, Sankar MJ, Paul VK, Deorari AK, Joshi M, Agarwal R: **Stepwise interventions for improving hand hygiene compliance in a level 3 academic neonatal intensive care unit in north India**. *Journal of Perinatology* 2021, **41**(12):2834-2839.
29. Chandonnet CJ, Kahlon PS, Rachh P, Degrazia M, Dewitt EC, Flaherty KA, Spigel N, Packard S, Casey D, Rachwal C *et al*: **Health care failure mode and effect analysis to reduce NICU line-associated bloodstream infections**. *Pediatrics* 2013, **131**(6):e1961-1969.
30. Almeida CC, Pissarra da Silva SMS, Flor de Lima Caldas de Oliveira FSD, Guimarães Pereira Areias MHF: **Nosocomial sepsis: evaluation of the efficacy of preventive measures in a level-III neonatal intensive care unit**. *J Matern Fetal Neonatal Med* 2017, **30**(17):2036-2041.
31. Azab SF, Sherbiny HS, Saleh SH, Elsaeed WF, Elshafiey MM, Siam AG, Arafa MA, Alghobashy AA, Bendary EA, Basset MA *et al*: **Reducing ventilator-associated pneumonia in neonatal intensive care unit using "VAP prevention Bundle": a cohort study**. *BMC Infect Dis* 2015, **15**:314.
