# Supplementary material for: Investigating the implementation of infection prevention and control practices in neonatal care across country income levels: a systematic review
Source: Antimicrob Resist Infect Control. 2025 Feb 7;14:8. doi: 10.1186/s13756-025-01516-7 (PMC11806577; doi:10.1186/s13756-025-01516-7)
Supplement: Supplementary file 5 — Additional File 5: Database Search Strategies. [file 13756_2025_1516_MOESM5_ESM.pdf]

Investigating the Implementation of Infection Prevention and Control Practices in Neonatal Care Across Country Income Levels: A Systematic Review

Emanuela Nyantakyi, Julia Baenziger, Laura Caci, Kathrin Blum, Aline Wolfensberger, Angela Dramowski, Bianca Albers, Marta Castro, Marie-Therese Schultes, Lauren Clack

SUPPLEMENTARY FILE

Database Search Strategies

Contents

Cumulative Index to Nursing and Allied Health Literature (CINAHL) 3
Cochrane Central Register of Controlled Trials (CENTRAL) 6
Excerpta Medica Database (Embase) 7
Medical Literature Analysis and Retrieval System Online (MEDLINE) 8
PsycINFO 11
Scopus 13
Web of Science 14

All searches were completed on January 19, 2023

Cumulative Index to Nursing and Allied Health Literature (CINAHL)

#	Query	Limiters/Expanders	Last Run Via	Results *n*
**S1**	(MH "Intensive Care, Neonatal+") OR (MH "Intensive Care Units, Neonatal") OR (MH "Nurseries, Hospital") OR (MH "Nursing Units") OR TI (nicu) OR AB (nicu) OR ((MH "Infant+") AND (TI ((intensive OR critical) N1 care) OR AB ((intensive OR critical) N1 care))) OR TI ((infant* OR newborn* OR neonat* OR baby OR babies) N3 (intensive OR critical) N1 care) OR AB ((infant* OR newborn* OR neonat* OR baby OR babies) N3 (intensive OR critical) N1 care) OR TI ((infant* OR newborn* OR neonat* OR matern*) N1 unit) OR AB ((infant* OR newborn* OR neonat* OR matern*) N1 unit) OR TI ((infant* OR newborn* OR neonat* OR baby OR babies) AND hospital*)	Expanders- Apply equivalent subjects Search modes- Find all my search terms	Interface- EBSCOhost Research Databases Search Screen- Advanced Search Database- CINAHL with Full Text	42,738
**S2**	(MH "Neonatal Sepsis") OR TI ((newborn OR neonatal) N3 (infection* OR sepsis OR septic*)) OR AB ((newborn OR neonatal) N3 (infection* OR sepsis OR septic*)) OR (((MH "Cross Infection+") OR (MH "Community- Acquired Infections+") OR (MH "Respiratory Tract Infections+") OR (MH "Bacterial Infections+") OR (MH "Virus Diseases+") OR (MH "Healthcare-Associated Pneumonia") OR (MH "Pneumonia+") OR (MH "Enterocolitis, Necrotizing") OR TI (infection* OR sepsis OR colonization OR colonisation OR carriage OR pneumonia) OR AB (infection* OR sepsis OR colonization OR colonization OR carriage OR pneumonia)) AND ((MH "Infant+") OR TI (infant* OR newborn* OR neonat* OR baby OR babies) OR AB (infant* OR newborn* OR neonat* OR baby OR babies)))	Expanders- Apply equivalent subjects Search modes- Find all my search terms	Interface- EBSCOhost Research Databases Search Screen- Advanced Search Database- CINAHL with Full Text	58,242
**S3**	(MH "Infection Control+") OR (MH "Neonatal Sepsis/PC") OR (MH "Cross Infection+/PC") OR (MH "Community-Acquired Infections+/PC") OR (MH "Respiratory Tract Infections+/PC") OR (MH "Bacterial Infections+/PC") OR (MH "Virus Diseases+/PC") OR (MH "Healthcare-Associated Pneumonia/PC") OR (MH "Pneumonia+/PC") OR (MH "Enterocolitis, Necrotizing/PC") OR (MH "Hygiene+") OR (MH "Hygiene") OR (MH "Handwashing+") OR TI ((infection OR sepsis OR outbreak) N9 (prevention OR prophylaxis OR control OR reduc*)) OR AB ((infection OR sepsis OR outbreak) N9 (prevention OR prophylaxis OR control OR reduc*)) OR TI ((hygiene OR desinfect* OR disinfect* OR cleaning OR sterili?ation) N3 (environment* OR equipement* OR device* OR material*)) OR AB ((hygiene OR desinfect* OR disinfect* OR cleaning OR sterili?ation) N3 (environment* OR equipement* OR device* OR material*)) OR TI ((hand OR skin) N3 (desinfect* OR disinfect* OR hygiene OR wash* OR scrub* OR saniti?ation OR sanitation OR cleansing)) OR AB ((hand OR skin) N3 (desinfect* OR disinfect* OR hygiene OR wash* OR scrub* OR saniti?ation OR sanitation OR cleansing)) OR TI handwashing OR AB handwashing OR TI ((infection	Expanders- Apply equivalent subjects Search modes- Find all my search terms	Interface- EBSCOhost Research Databases Search Screen- Advanced Search Database- CINAHL with Full Text	199,023

#	Query	Limiters/Expanders	Last Run Via	Results *n*
	OR sepsis OR outbreak) N3 (intervention* OR protocol* OR guideline* OR consensus OR practice*)) OR AB ((infection OR sepsis OR outbreak) N3 (intervention* OR protocol* OR guideline* OR consensus OR practice*))			
**S4**	(MH "Kangaroo Care") OR (MH "Probiotics") OR (MH "Breast Feeding") OR (MH "Emollients") OR TI (("skin to skin") N3 (care OR contact OR method OR position OR program* OR holding OR style)) OR AB (("skin to skin") N3 (care OR contact OR method OR position OR program* OR holding OR style)) OR TI (synbiotic* OR probiotic* OR breastfeeding OR breast-feeding OR emollient OR antisepsis OR "whole body bathing" OR "chlorhexidine gluconate") OR AB (synbiotic* OR probiotic* OR breastfeeding OR breast-feeding OR emollient OR antisepsis OR "whole body bathing" OR "chlorhexidine gluconate") OR TI ((breast OR human) N1 milk) OR AB ((breast OR human) N1 milk)	Expanders- Apply equivalent subjects Search modes- Find all my search terms	Interface- EBSCOhost Research Databases Search Screen- Advanced Search Database- CINAHL with Full Text	54,292
**S5**	S3 OR S4	Expanders- Apply equivalent subjects Search modes- Find all my search terms	Interface- EBSCOhost Research Databases Search Screen- Advanced Search Database- CINAHL with Full Text	248,994
**S6**	(MH "Program Implementation") OR (MH "Implementation Science") OR (MH "Quality Improvement") OR (MH "Program Evaluation") OR (MH "Guideline Adherence") OR TI (implement* OR launch* OR instituted OR embed* OR insert* OR facilitator* OR barrier* OR determinants OR factor OR factors OR approach*) OR AB (implement* OR launch* OR instituted OR embed* OR insert* OR facilitator* OR barrier* OR determinants OR factor OR factors OR approach*) OR TI (knowledge N3 (translation OR transfer)) OR AB (knowledge N3 (translation OR transfer)) OR TI ((process OR formative*) N3 (evaluat* OR assess*)) OR AB ((process OR formative*) N3 (evaluat* OR assess*)) OR TI (quality N2 improv*) OR AB (quality N2 improv*) OR TI ((change OR quality)	Expanders- Apply equivalent subjects Search modes- Find all my search terms	Interface- EBSCOhost Research Databases Search Screen- Advanced Search Database- CINAHL with Full Text	2,648,959
**S7**	S1 AND S2 AND S5 AND S6	Expanders- Apply equivalent subjects Search modes- Find all my search terms	Interface- EBSCOhost Research Databases Search Screen- Advanced Search Database- CINAHL with Full Text	1,477
**S8**	S1 AND S2 AND S5 AND S6	Limiters - Language: Danish, English, French, German,	Interface- EBSCOhost Research Databases	1,434

#	Query	Limiters/Expanders	Last Run Via	Results *n*
		Italian, Norwegian, Spanish<br>Expanders- Apply equivalent subjects<br>Search modes- Find all my search terms	Search Screen- Advanced Search Database- CINAHL with Full Text	

Cochrane Central Register of Controlled Trials (CENTRAL)

#	Search	Results
#1	nicu:ti,ab,kw OR ((infant* OR newborn* OR neonat* OR baby OR babies) NEAR/3 (intensive OR critical) NEAR/1 care):ti,ab,kw OR ((infant* OR newborn* OR neonat* O matern*) NEAR/1 unit):ti,ab,kw OR ((infant* OR newborn* OR neonat* OR baby OR babies) AND hospital*):ti	7,844
#2	((newborn OR neonatal) NEAR/3 (infection* OR sepsis OR septic*)):ti,ab,kw OR ((infection* OR sepsis OR colonization OR colonisation OR carriage OR pneumonia):ti,ab,kw AND (infant* OR newborn* OR neonat* OR baby OR babies):ti,ab,kw)	15,525
#3	((infection OR sepsis OR outbreak) NEAR/9 (prevention OR prophylaxis OR control OR reduc*)):ti,ab,kw OR ((hygiene OR desinfect* OR disinfect* OR cleaning OR sterili?ation) NEAR/3 (environment* OR equipement* OR device* OR material*)):ti,ab,kw OR ((hand OR skin) NEAR/3 (desinfect* OR disinfect* OR hygiene OR wash* OR scrub* OR saniti?ation OR sanitation OR cleansing)):ti,ab,kw OR handwashing:ti,ab,kw OR ((infection OR sepsis OR outbreak) NEAR/3 (intervention* OR protocol* OR guideline* OR consensus OR practice*)):ti,ab,kw	24,670
#4	(("skin to skin") NEAR/3 (care OR contact OR method OR position OR program* OR holding OR style)):ti,ab,kw OR (synbiotic* OR probiotic* OR breastfeeding OR breastfeeding OR emollient OR antisepsis OR "whole body bathing" OR "chlorhexidine gluconate"):ti,ab,kw OR ((breast OR human) NEAR/1 milk):ti,ab,kw	23,329
#5	(implement* OR launch* OR instituted OR embed* OR insert* OR facilitator* OR barrier* OR determinants OR factor OR factors OR approach*):ti,ab,kw OR (knowledge NEAR/3 (translation OR transfer)):ti,ab,kw OR ((process OR formative*) NEAR/3 (evaluat* OR assess*)):ti,ab,kw OR (quality NEAR/2 improv*):ti,ab,kw OR ((change OR quality) NEAR/1 management):ti,ab,kw OR (innovation* OR program* OR strategy OR strategies OR initiative* OR improvement* OR investigation* OR measures OR bundle* OR project* OR plan*):ti,ab,kw	877,586
#6	#1 AND #2 AND (#3 OR #4) AND #5	318

Excerpta Medica Database (Embase)

#	Search	Results
#1	'newborn intensive care'/exp OR 'neonatal intensive care unit'/exp OR 'nursery'/exp OR nicu:ti,ab OR ('infant'/exp AND (((intensive OR critical) NEAR/1 care):ti,ab)) OR (((infant* OR newborn* OR neonat* OR baby OR babies) NEAR/3 (intensive OR critical) NEAR/1 care):ti,ab) OR (((infant* OR newborn* OR neonat* OR matern*) NEAR/1 unit):ti,ab) OR ((infant*:ti OR newborn*:ti OR neonat*:ti OR baby:ti OR babies:ti) AND hospital*:ti)	94,947
#2	'newborn sepsis'/exp OR 'newborn infection'/exp OR (((newborn OR neonatal) NEAR/3 (infection* OR sepsis OR septic*)):ti,ab) OR (('communicable disease'/exp OR 'cross infection'/exp OR 'device infection'/exp OR 'healthcare associated infection'/exp OR 'hospital infection'/exp OR 'respiratory tract infection'/exp OR 'bacterial infection'/exp OR 'virus infection'/exp OR 'asymptomatic infection'/exp OR 'pneumonia'/exp OR 'necrotizing enterocolitis'/exp OR infection*:ti,ab OR sepsis:ti,ab OR colonization:ti,ab OR colonisation:ti,ab OR carriage:ti,ab OR pneumonia:ti,ab) AND ('infant'/exp OR infant*:ti,ab OR newborn*:ti,ab OR neonat*:ti,ab OR baby:ti,ab OR babies:ti,ab))	315,543
#3	infection prevention'/exp OR 'infection control'/exp OR 'hygiene'/exp OR 'disinfection'/exp OR 'hand washing'/exp OR 'hand sanitizer'/exp OR (((infection OR sepsis OR outbreak) NEAR/9 (prevention OR prophylaxis OR control OR reduc*)):ti,ab) OR (((hygiene OR desinfect* OR disinfect* OR cleaning OR sterili?ation) NEAR/3 (environment* OR equipement* OR device* OR material*)):ti,ab) OR (((hand OR skin) NEAR/3 (desinfect* OR disinfect* OR hygiene OR wash* OR scrub* OR saniti?ation OR sanitation OR cleansing)):ti,ab) OR handwashing:ti,ab OR (((infection OR sepsis OR outbreak) NEAR/3 (intervention* OR protocol* OR guideline* OR consensus OR practice*)):ti,ab)	462,349
#4	'kangaroo care'/exp OR 'probiotic agent'/exp OR 'breast feeding'/exp OR 'emollient agent'/exp OR 'antisepsis'/exp OR 'chlorhexidine gluconate'/exp OR (('skin to skin' NEAR/3 (care OR contact OR method OR position OR program* OR holding OR style)):ti,ab) OR synbiotic*:ti,ab OR probiotic*:ti,ab OR breastfeeding:ti,ab OR 'breast feeding':ti,ab OR emollient:ti,ab OR antisepsis:ti,ab OR 'whole body bathing':ti,ab OR 'chlorhexidine gluconate':ti,ab OR (((breast OR human) NEAR/1 milk):ti,ab)	177,337
#5	#3 OR #4	628,138
#6	'implementation science'/exp OR 'process evaluation'/exp OR 'knowledge translation'/exp OR 'knowledge transfer'/exp OR 'formative assessment'/exp OR 'protocol compliance'/exp OR 'conceptual framework'/exp OR 'total quality management'/exp OR 'change management'/exp OR implement*:ti,ab OR launch*:ti,ab OR instituted:ti,ab OR embed*:ti,ab OR insert*:ti,ab OR facilitator*:ti,ab OR barrier*:ti,ab OR determinants:ti,ab OR factor:ti,ab OR factors:ti,ab OR approach*:ti,ab OR ((knowledge NEAR/3 (translation OR transfer)):ti,ab) OR (((process OR formative*) NEAR/3 (evaluat* OR assess*)):ti,ab) OR ((quality NEAR/2 improv*):ti,ab) OR (((change OR quality) NEAR/1 management):ti,ab) OR innovation*:ti,ab OR program*:ti,ab OR strategy:ti,ab OR strategies:ti,ab OR initiative*:ti,ab OR improvement*:ti,ab OR investigation*:ti,ab OR measures:ti,ab OR bundle*:ti,ab OR project*:ti,ab OR plan*:ti,a	14,285,308
#7	#1 AND #2 AND #5 AND #6	3,607
#8	#1 AND #2 AND #5 AND #6 NOT [conference abstract]/lim AND ([danish]/lim OR [english]/lim OR [french]/lim OR [german]/lim OR [italian]/lim OR [norwegian]/lim OR [spanish]/lim)	2,628

Medical Literature Analysis and Retrieval System Online (MEDLINE)

#	Query	Limiters/Expanders	Last Run Via	Results *n*
**S1**	(MH "Intensive Care, Neonatal") OR (MH "Intensive Care Units, Neonatal") OR (MH "Nurseries, Infant") OR (MH "Nurseries, Hospital") OR TI (nicu) OR AB (nicu) OR ((MH "Infant+") AND (TI ((intensive OR critical) N1 care) OR AB ((intensive OR critical) N1 care))) OR TI ((infant* OR newborn* OR neonat* OR baby OR babies) N3 (intensive OR critical) N1 care) OR AB ((infant* OR newborn* OR neonat* OR baby OR babies) N3 (intensive OR critical) N1 care) OR TI ((infant* OR newborn* OR neonat* OR matern*) N1 unit) OR AB ((infant* OR newborn* OR neonat* OR matern*) N1 unit) OR TI ((infant* OR newborn* OR neonat* OR baby OR babies) AND hospital*)	Expanders- Apply equivalent subjects Search modes - Find all my search terms	Interface- EBSCOhost Research Databases Search Screen- Advanced Search Database - MEDLINE	68,106
**S2**	(MH "Neonatal Sepsis") OR TI ((newborn OR neonatal) N3 (infection* OR sepsis OR septic*)) OR AB ((newborn OR neonatal) N3 (infection* OR sepsis OR septic*)) OR (((MH "Cross Infection+") OR (MH "Communicable Diseases+") OR (MH "Bacterial Infections+") OR (MH "Virus Diseases+") OR (MH "Healthcare-Associated Pneumonia+") OR (MH "Respiratory Tract Infections+") OR (MH "Asymptomatic Infections") OR (MH "Pneumonia+") OR (MH "Enterocolitis, Necrotizing") OR TI (infection* OR sepsis OR colonization OR colonization OR carriage OR pneumonia) OR AB (infection* OR sepsis OR colonization OR colonization OR carriage OR pneumonia)) AND ((MH "Infant+") OR TI (infant* OR newborn* OR neonat* OR baby OR babies) OR AB (infant* OR newborn* OR neonat* OR baby OR babies)))	Expanders- Apply equivalent subjects Search modes - Find all my search terms	Interface- EBSCOhost Research Databases Search Screen- Advanced Search Database - MEDLINE	300,736
**S3**	(MH "Infection Control+") OR (MH "Neonatal Sepsis/PC") OR (MH "Cross Infection+/PC") OR (MH "Communicable Diseases+/PC ") OR (MH "Bacterial Infections+/PC ") OR (MH "Virus Diseases+/PC ") OR (MH "Healthcare-Associated Pneumonia+/PC ") OR (MH "Respiratory Tract Infections+/PC ") OR (MH "Asymptomatic Infections/PC ") OR (MH "Pneumonia+/PC ") OR (MH "Enterocolitis, Necrotizing/PC ") OR (MH "Hygiene+") OR (MH "Hand Hygiene") OR (MH "Hand Disinfection") OR (MH "Hand Sanitizers") OR TI ((infection OR sepsis OR outbreak) N9 (prevention OR prophylaxis OR control OR reduc*)) OR AB ((infection OR sepsis OR outbreak) N9 (prevention OR prophylaxis OR control OR reduc*)) OR TI ((hygiene OR desinfect* OR disinfect* OR cleaning OR sterili?ation) N3 (environment* OR equipement* OR device* OR material*)) OR AB ((hygiene OR desinfect* OR disinfect* OR cleaning OR sterili?ation) N3 (environment* OR equipement* OR device* OR material*)) OR TI ((hand	Expanders- Apply equivalent subjects Search modes - Find all my search terms	Interface- EBSCOhost Research Databases Search Screen- Advanced Search Database - MEDLINE	586,272

#	Query	Limiters/Expanders	Last Run Via	Results *n*
**S4**	MH "Kangaroo-Mother Care Method") OR (MH "Probiotics+") OR (MH "Breast Feeding+") OR (MH "Emollients") OR (MH "Antisepsis+") OR OR TI (("skin to skin") N3 (care OR contact OR method OR position OR program* OR holding OR style)) OR AB (("skin to skin") N3 (care OR contact OR method OR position OR program* OR holding OR style)) OR TI (synbiotic* OR probiotic* OR breastfeeding OR breastfeeding OR emollient OR antisepsis OR "whole body bathing" OR "chlorhexidine gluconate") OR AB (synbiotic* OR breastfeeding OR probiotic* OR breast-feeding OR emollient OR antisepsis OR "whole body bathing" OR "chlorhexidine gluconate") OR TI ((breast OR human) N1 milk) OR AB ((breast OR human) N1 milk)	Expanders- Apply equivalent subjects Search modes - Find all my search terms	Interface- EBSCOhost Research Databases Search Screen- Advanced Search Database - MEDLINE	121,930
**S5**	S3 OR S4	Expanders- Apply equivalent subjects Search modes - Find all my search terms	Interface- EBSCOhost Research Databases Search Screen- Advanced Search Database - MEDLINE	698,730
**S6**	(MH "Implementation Science") OR (MH "Health Plan Implementation") OR (MH "Program Evaluation+") (MH "Translational Science, Biomedical") OR (MH "Guideline Adherence") OR (MH "Total Quality Management") OR (MH "Change Management") OR TI (implement* OR launch* OR instituted OR embed* OR insert* OR facilitator* OR barrier* OR determinants OR factor OR factors OR approach*) OR AB (implement* OR launch* OR instituted OR embed* OR insert* OR facilitator* OR barrier* OR determinants OR factor OR factors OR approach*) OR TI (knowledge N3 (translation OR transfer)) OR AB (knowledge N3 (translation OR transfer)) OR TI ((process OR formative*) N3 (evaluat* OR assess*)) OR AB ((process OR formative*) N3 (evaluat* OR assess*)) OR TI (quality N2 improv*) OR AB (qualityN2 improv*) OR TI ((change OR quality) N1 management) OR AB ((change OR quality) N1 management) OR TI (innovation* OR program* OR strategy OR strategies OR initiative* OR improvement* OR investigation* OR measures OR bundle* OR project* OR plan*) OR AB (innovation* OR program* OR strategy OR strategies OR initiative* OR improvement* OR investigation* OR measures OR bundle* OR project* OR plan*)	Expanders- Apply equivalent subjects Search modes - Find all my search terms	Interface- EBSCOhost Research Databases Search Screen- Advanced Search Database - MEDLINE	11,351,686
**S7**	S1 AND S2 AND S5 AND S6	Expanders- Apply equivalent subjects Search modes - Find all my search terms	Interface- EBSCOhost Research Databases Search Screen- Advanced Search Database - MEDLINE	3,169
**S8**	S1 AND S2 AND S5 AND S6	Limiters - Language: Danish, English, French, German,	Interface- EBSCOhost Research Databases Search Screen- Advanced	3,081

#	Query	Limiters/Expanders	Last Run Via	Results *n*
		Italian, Norwegian, Spanish<br>Expanders – Apply equivalent subjects<br>Search modes - Find all my search terms	Search Database- MEDLINE	

PsycINFO

#	Query	Limiters/Expanders	Last Run Via	Results *n*
**S1**	DE "Neonatal Intensive Care" OR TX (nicu) OR (AG (infancy OR neonatal) AND TX ((intensive OR critical) N1 care)) OR TX ((infant* OR newborn* OR neonat* OR baby OR babies) N3 (intensive OR critical) N1 care) OR TX ((infant* OR newborn* OR neonat* OR matern*) N1 unit) OR TI ((infant* OR newborn* O neonat* OR baby OR babies) AND hospital*)	Expanders- Apply equivalent subjects Search modes- Find all my search terms	Interface- EBSCOhost Research Databases Search Screen- Advanced Search Database - APA PsycInfo	5,933
**S2**	TX ((newborn OR neonatal) N3 (infection* OR sepsis OR septic*)) OR ((DE "Bacterial Infections" OR DE "Bacterial Meningitis" OR DE "Pneumonia" OR DE "Tuberculosis" OR DE "Viral Infections" OR DE "Coronavirus" OR DE "Encephalitis" OR DE "Epstein Barr Viral Disorder" OR DE "Herpes Genitalis" OR DE "Herpes Simplex" OR DE "Human Papillomavirus" OR DE "Influenza" OR DE "Measles" OR DE "Poliomyelitis" OR DE "Retrovirus Infections" OR DE "Rubella" OR DE "Viral Variants" OR TX (infection* OR sepsis OR colonization OR colonisation OR carriage OR pneumonia)) AND (AG (infancy OR neonatal) OR TX (infant* OR newborn* OR neonat* OR baby OR babies)))	Expanders- Apply equivalent subjects Search modes- Find all my search terms	Interface- EBSCOhost Research Databases Search Screen- Advanced Search Database - APA PsycInfo	4,877
**S3**	TX ((infection OR sepsis OR outbreak) N9 (prevention OR prophylaxis OR control OR reduc*)) OR TX ((hygiene OR desinfect* OR disinfect* OR cleaning OR sterili?ation) N3 (environment* OR equipement* OR device* OR material*)) OR TX ((hand OR skin) N3 (desinfect* OR disinfect* OR hygiene OR wash* OR scrub* OR saniti?ation OR sanitation OR cleansing)) OR TX handwashing OR TX ((infection OR sepsis OR outbreak) N3 (intervention* OR protocol* OR guideline* OR consensus OR practice*))	Expanders- Apply equivalent subjects Search modes- Find all my search terms	Interface- EBSCOhost Research Databases Search Screen- Advanced Search Database - APA PsycInfo	8,596
**S4**	DE "Breast Feeding" OR TX (("skin to skin") N3 (care OR contact OR method OR position OR program* OR holding OR style)) OR TX (synbiotic* OR probiotic* OR breastfeeding OR breastfeeding OR emollient OR antisepsis OR "whole body bathing" OR "chlorhexidine gluconate") OR TX ((breast OR human) N1 milk)	Expanders- Apply equivalent subjects Search modes- Find all my search terms	Interface- EBSCOhost Research Databases Search Screen- Advanced Search Database - APA PsycInfo	7,781
**S5**	S3 OR S4	Expanders- Apply equivalent subjects Search modes- Find all my search terms	Interface- EBSCOhost Research Databases Search Screen- Advanced Search Database - APA PsycInfo	16,276

#	Query	Limiters/Expanders	Last Run Via	Results *n*
**S6**	(DE "Program Evaluation") OR (DE "Quality Control") OR TX (implement* OR launch* OR instituted OR embed* OR insert* OR facilitator* OR barrier* OR determinants OR factor OR factors OR approach*) OR TX (knowledge N3 (translation OR transfer)) OR TX ((process OR formative*) N3 (evaluat* OR assess*)) OR TX (quality N2 improv*) OR TX ((change OR quality) N1 management) OR TX (innovation* OR program* OR strategy OR strategies OR initiative* OR improvement* OR investigation* OR measures OR bundle* OR project* OR plan*)	Expanders- Apply equivalent subjects Search modes- Find all my search terms	Interface- EBSCOhost Research Databases Search Screen- Advanced Search Database - APA PsycInfo	3,052,982
**S7**	S1 AND S2 AND S5 AND S6	Expanders- Apply equivalent subjects Search modes- Find all my search terms	Interface- EBSCOhost Research Databases Search Screen- Advanced Search Database - APA PsycInfo	50
**S8**	S1 AND S2 AND S5 AND S6	Limiters - Language: Danish, English, French, German, Italian, Norwegian, Spanish Expanders- Apply equivalent subjects Search modes - Find all my search terms	Interface- EBSCOhost Research Databases Search Screen- Advanced Search Database - APA PsycInfo	50

Scopus

	Results n
**Query String**<br>TITLE-ABS-KEY(innovation* OR program* OR strategy OR strategies OR initiative* OR improvement* OR investigation* OR measures OR bundle* OR project* OR plan*)	
**Search**<br>(((TITLE-ABS-KEY (nicu) OR TITLE-ABS-KEY ((infant* OR newborn* OR neonat* OR baby OR babies) W/3 ("intensive care" OR "critical care")) OR TITLE-ABSKEY ((infant* OR newborn* OR neonat* OR matern*) W/1 unit) OR (TITLE-ABSKEY (infant* OR newborn* OR neonat* OR baby OR babies) AND TITLE (hospital*))) AND (TITLE-ABS-KEY ((newborn OR neonatal) W/3 (infection* OR sepsis OR septic*)) OR TITLE-ABS-KEY ((infection* OR sepsis OR colonization OR colonisation OR carriage OR pneumonia) AND (infant* OR newborn* OR neonat* OR baby OR babies)))) AND ((TITLE-ABSKEY ((infection OR sepsis OR outbreak) W/9 (prevention OR prophylaxis OR control OR reduc*)) OR TITLE-ABS-KEY ((hygiene OR desinfect* OR disinfect* OR cleaning OR sterili?ation) W/3 (environment* OR equipement* OR device* OR material*)) OR TITLE-ABS-KEY ((hand OR skin) W/3 (desinfect* OR disinfect* OR hygiene OR wash* OR scrub* OR saniti?ation OR sanitation OR cleansing)) OR TITLE-ABS-KEY (handwashing) OR TITLE-ABS-KEY ((infection OR sepsis OR outbreak) W/3 (intervention* OR protocol* OR guideline* OR consensus OR practice*))) OR (TITLE-ABS-KEY (("skin to skin") W/3 (care OR contact OR method OR position OR program* OR holding OR style)) OR TITLEABS- KEY (synbiotic* OR probiotic* OR breastfeeding OR breast-feeding OR emollient OR antisepsis OR "whole body bathing" OR "chlorhexidine gluconate") OR TITLE-ABS-KEY ((breast OR human) W/1 milk)))) AND ((TITLE-ABSKEY (implement* OR launch* OR instituted OR embed* OR insert* OR facilitator* OR barrier* OR determinants OR factor OR factors OR approach*)) OR (TITLEABS- KEY (knowledge W/3 (translation OR transfer))) OR (TITLE-ABS-	4,339

Web of Science

#	Search	Results n
#1	TS=nicu OR TS=((infant* OR newborn* OR neonat* OR baby OR babies) NEAR/3 ("intensive care" OR "critical care")) OR TS=((infant* OR newborn* OR neonat* OR matern*) NEAR/1 unit) OR (TS=(infant* OR newborn* OR neonat* OR baby OR babies) AND TI=(hospital*))	49,100
#2	TS=((newborn OR neonatal) NEAR/3 (infection* OR sepsis OR septic*)) OR TS=((infection* OR sepsis OR colonization OR colonisation OR carriage OR pneumonia) AND (infant* OR newborn* OR neonat* OR baby OR babies))	108,435
#3	TS=((infection OR sepsis OR outbreak) NEAR/9 (prevention OR prophylaxis OR control OR reduc*)) OR TS=((hygiene OR desinfect* OR disinfect* OR cleaning OR sterili?ation) NEAR/3 (environment* OR equipement* OR device* OR material*)) OR TS=((hand OR skin) NEAR/3 (desinfect* OR disinfect* OR hygiene OR wash* OR scrub* OR saniti?ation OR sanitation OR cleansing)) OR TS=handwashing OR TS=((infection OR sepsis OR outbreak) NEAR/3 (interven□on* OR protocol* OR guideline* OR consensus OR prac□ce*))	240,400
#4	TS=(("skin to skin") NEAR/3 (care OR contact OR method OR position OR program* OR holding OR style)) OR TS=(synbiotic* OR probiotic* OR breastfeeding OR breast-feeding OR emollient OR antisepsis OR "whole body bathing" OR "chlorhexidine gluconate") OR TS=((breast OR human) NEAR/1 milk)	134,081
#5	TS=(implement* OR launch* OR instituted OR embed* OR insert* OR facilitator* OR barrier* OR determinants OR factor OR factors OR approach*) OR TS=(knowledge NEAR/3 (translation OR transfer)) OR TS=((process OR formative*) NEAR/3 (evaluat* OR assess*)) OR TS=(quality NEAR/2 improv*) OR TS=((change OR quality) NEAR/1 management) OR TS=(innovation* OR program* OR strategy OR strategies OR initiative* OR improvement* OR inves□ga□on* OR measures OR bundle* OR project* OR plan*)	2,676,1334
#6	#1 AND #2 AND (#3 OR #4) AND #5	2,269
#7	#1 AND #2 AND (#3 OR #4) AND #5 and English or Spanish or French or German or Italian (Languages)	2,246
